# Extreme summers impact cropland and grassland soil microbiomes

**DOI:** 10.1038/s41396-023-01470-5

**Published:** 2023-07-07

**Authors:** Qicheng Bei, Thomas Reitz, Beatrix Schnabel, Nico Eisenhauer, Martin Schädler, François Buscot, Anna Heintz-Buschart

**Affiliations:** 1 German Centre for Integrative Biodiversity Research (iDiv) Halle-Jena-Leipzig, Leipzig, Germany; 2 Department of Soil Ecology, Helmholtz Centre for Environmental Research - UFZ, Halle (Saale), Germany; 3 Institute of Biology, Leipzig University, Leipzig, Germany; 4 Department of Community Ecology, Helmholtz Centre for Environmental Research - UFZ, Halle (Saale), Germany; 5 Swammerdam Institute for Life Sciences, University of Amsterdam, Amsterdam, The Netherlands

**Keywords:** Climate-change impacts, Soil microbiology

## Abstract

The increasing frequency of extreme weather events highlights the need to understand how soil microbiomes respond to such disturbances. Here, metagenomics was used to investigate the effects of future climate scenarios (+0.6 °C warming and altered precipitation) on soil microbiomes during the summers of 2014–2019. Unexpectedly, Central Europe experienced extreme heatwaves and droughts during 2018–2019, causing significant impacts on the structure, assembly, and function of soil microbiomes. Specifically, the relative abundance of Actinobacteria (bacteria), Eurotiales (fungi), and Vilmaviridae (viruses) was significantly increased in both cropland and grassland. The contribution of homogeneous selection to bacterial community assembly increased significantly from 40.0% in normal summers to 51.9% in extreme summers. Moreover, genes associated with microbial antioxidant (Ni-SOD), cell wall biosynthesis (*glmSMU*, *murABCDEF*), heat shock proteins (GroES/GroEL, Hsp40), and sporulation (*spoIID*, *spoVK*) were identified as potential contributors to drought-enriched taxa, and their expressions were confirmed by metatranscriptomics in 2022. The impact of extreme summers was further evident in the taxonomic profiles of 721 recovered metagenome-assembled genomes (MAGs). Annotation of contigs and MAGs suggested that Actinobacteria may have a competitive advantage in extreme summers due to the biosynthesis of geosmin and 2-methylisoborneol. Future climate scenarios caused a similar pattern of changes in microbial communities as extreme summers, but to a much lesser extent. Soil microbiomes in grassland showed greater resilience to climate change than those in cropland. Overall, this study provides a comprehensive framework for understanding the response of soil microbiomes to extreme summers.

## Introduction

In the context of global warming, extreme weather events (EWEs), such as heatwaves and droughts, have become more frequent and intense [1, 2]. In Europe, extreme summers have occurred more frequently in recent years (e.g., 2003, 2010, 2018), leading to extreme droughts and reduced crop yields [3]. For example, in 2018, the air temperature exceeded the long-term average by more than 3.3 °C from April to October, precipitation was <40% of normal [4], and crop yield was reduced by up to 50% in Central Europe [5]. Soil microorganisms play vital roles in ecosystem functioning, including supporting plant growth, nutrient cycling, and organic matter decomposition [6, 7]. Many studies conducted in the past decades have investigated the effects of climate manipulation on soil microbes [8, 9]. Artificially increasing the ecosystem temperature by 2–3 °C through infrared radiation has been shown to significantly impact the soil microbial community structure in cropland [10, 11] and grassland [12, 13]. However, our current understanding of how soil microbial communities and functions respond to naturally occurring EWEs is still limited [14], despite their crucial role in predicting global changes.

Warming and drought have long been observed to increase the abundance of Gram-positive bacteria, particularly Actinobacteria [8, 9]. Recent advancements in metagenomics have allowed the recovery of metagenome-assembled genomes (MAGs) from complex soil microbial communities, providing insights into microbial functional traits associated with permafrost thawing [15, 16] and drought [17]. For example, MAG analysis has revealed a connection between actinobacterial phenotypes and iron metabolism in drought-stressed sorghum rhizosphere [17]. Regarding soil fungi, studies have indicated an increase in the relative abundance of soil-borne fungal plant pathogens under future climate conditions [18, 19]. In addition, the ecological roles of soil viruses are increasingly recognized through omics-based methods. Direct evidence of plant-derived carbon transfer to bacteria and phages has been obtained by combined ^13^CO_2_-stable isotope probing and metagenomics [20]. Long-term soil warming has been found to stimulate virus-host interactions in a prairie in Oklahoma, USA [21]. With increasing access to metagenomic datasets, further investigations are needed to enhance our understanding of how climate and land-use changes impact hosts and associated viruses [22].

In this study, we used metagenomics to investigate the impact of climate change on soil microbiomes from 2014 to 2019, and confirmed their activities by metatranscriptomics performed in 2022. The study was conducted at the Global Change Experimental Facility (GCEF) research station in Central Germany [23], where climate manipulation was implemented to simulate projected climatic scenarios for 2070–2100. A total of 180 soil samples were collected between July and September from three different land-use types during 2014–2019 (Figs. S1–S2). Unexpectedly, Central Europe experienced an extreme drought that began in spring 2018 and persisted until fall 2019. The consecutive drought event was unprecedented in Europe over the last 250 years [24], presenting a unique opportunity to investigate the impact of extreme summers on soil microbiomes. In our previous study, we found a significant decrease in soil microbial activity and biomass during the period of 2018–2019, in contrast to the preceding years of 2015–2017 at the GCEF [25]. In the current study, our main questions are as follows: (1) how do extreme summers affect the community structure of soil microbiomes, including archaea, bacteria, fungi, and viruses; (2) how do extreme summers affect the biochemical processes involved in C, N, S, and P cycling, as well as stress responses; and (3) whether and how climate and land-use changes shape the soil microbial community structure, assembly, and function.

## Materials and methods

### Study site and experimental design

The Global Change Experimental Facility (GCEF) is located at the UFZ field research station in Bad Lauchstädt, Saxony-Anhalt, Germany (51°23’30"N, 11°52’49"E, 116 m a.s.l.). The region has a subcontinental climate with an average annual precipitation of 516 mm and a mean temperature of 9.8 °C (1995–2014). The soil type at this site is Haplic Chernozem (around 70% silt and 20% clay content), with a high organic carbon content and water holding capacity [23]. Established in 2013, the GCEF serves as a large-scale field research platform to investigate the interactive effects of climate and land-use changes on terrestrial ecosystems. The facility consists of 10 blocks with 5 plots each, resulting in a total of 50 plots (each 16 m × 24 m). Half of the plots are exposed to future climate consensus scenarios for the years 2070–2100, based on three climate models (COSMO-CLM, REMO, and RCAO) [23], while the other half are subjected to ambient climate conditions. The simulated climate manipulation is achieved by automatically closing the roof and side panels from calendrical sundown to sunrise, resulting in an average air temperature increase of ~0.6 °C. An automatic irrigation system is utilized to reduce rainfall by 20% in summer and increase it by 10% in spring and fall [23].

Soil samples were taken from three land-use types: conventional farming (CF), organic farming (OF), and intensive grassland (IG). The CF crop rotation includes rapeseed, wheat, and barley, while the OF treatment involves sowing N-fixing legumes. The IG treatment is characterized by a selection of five grass cultivars, with the plots being mowed up to four times annually. Mineral fertilizers (N, P, K) are applied to CF and IG plots, while OF plots solely receive P and K fertilizers, without the use of N fertilizer. See Schädler et al. [23] and Table S1 for detailed descriptions of GCEF station and fertilization practices.

### Soil sampling and measurement of soil properties

From 2014 to 2019, soil samples were collected annually between July and September using a steel core sampler (2 cm diameter, 15 cm deep). In each plot, 20 soil cores were collected and combined to form a composite sample. A total of 180 soil samples (2 climates × 3 land-use types × 5 replicates × 6 summers) were collected. The soil samples were stored at −80 °C after removing roots and litter debris, and sieving to 2 mm. Soil pH and the content of mineral nitrogen forms, total nitrogen, and carbon were determined in our laboratory. A detailed analysis of soil properties can be found in the Supplementary Methods.

### DNA extraction and metagenome sequencing

Soil DNA extraction was performed using the DNeasy PowerSoil kit (Qiagen, Valencia, CA, USA) following the manufacturer’s instructions. Subsequently, metagenomic library preparation, and DNA sequencing (2 × 150 bp) were carried out using the NextSeq 500 system (Illumina) by the NGS Competence Center Tübingen (NCCT).

### Metagenomic assembly, binning, and annotation

The 180 metagenome libraries were subjected to quality control with the Read_QC module using metaWRAP v1.3 [26]. The processed reads from each sample were assembled individually using MEGAHIT [27], and short contigs (<1000 bp) were removed from the assemblies. Metagenome binning was performed using MetaBAT2 [28] within the metaWRAP. The quality of MAGs was evaluated using CheckM v1.2.0 [29], and the taxonomic classification of MAGs was determined using GTDB-Tk v2.0.0 [30] with the R07-RS207 database. Then, all MAGs were combined and dereplicated at 95% average nucleotide identity (ANI) using dRep v3.4.1 [31].

The contigs and MAGs were analyzed for open reading frames (ORFs) using Prodigal v2.6.3 [32]. Potential functions of the ORFs in each metagenome dataset were identified by searching against carbohydrate-active enzymes (CAZymes) and Kyoto Encyclopedia of Genes and Genomes (KEGG) orthologs (KOs) using DRAM v1.3 [33]. For KEGG annotation, DRAM searches were performed with HMMER on the KOfam database using gene specific e-value and percent coverage cutoffs. We also carried out a secondary metabolite biosynthetic gene clusters (BGCs) search based on contigs > 5 kb and all MAGs using antiSMASH v6.1 [34] (>50% sequence identity). Taxonomic assignments of KEGG KOs, CAZymes, and BGCs were obtained by aligning the NCBI NR database using DIAMOND v2.0.15 [35] (-sensitive, -e 1e-5), and the outputs were summarized using MEGAN v6.24.1 [36] with default parameters. To calculate the relative abundances of MAGs, KOs, CAZymes, and BGCs, each metagenome dataset was mapped against the reference sequences (bwa-men; identity 0.95, aligned percent 0.90), and read counts were normalized to per kilobase per million mapped reads (RPKM) using CoverM v0.4.0 (https://github.com/wwood/CoverM).

### Analysis of microbial community composition and diversity

Taxonomic classification of the metagenomic data was performed based on various datasets, including FASTQ reads, contigs, SSU rRNA genes, and MAGs (Fig. S4). For the FASTQ reads and contigs, taxonomy was determined by Kaiju v1.9.0 [37]. The SSU rRNA genes were extracted from metagenomic data using SortMeRNA v2.1 [38] and searched against the SILVA SSU database v138.1 [39]. The alpha-diversity of metagenomics was calculated using SingleM v0.13.2 [40], and the Shannon index was calculated using the phyloseq v1.34.0 package [41]. Detailed taxonomic analysis can be found in the Supplementary Methods.

### Analysis of soil DNA viruses

To identify potential DNA virus sequences, VirSorter2 v2.2.3 [42] and CheckV v1.0.1 [43] were applied to contigs >5 kb. Only contigs with a VirSorter2 score ≥ 0.9, containing at least one hallmark gene, and at least one viral gene from CheckV were retained and clustered. The viral operational taxonomic units (vOTUs) and MAGs were further linked via clustered regularly interspaced short palindromic repeats (CRISPR) spacers (BLASTn, query coverage ≥ 95% and ≤1 mismatch) and shared genomic content (ANI ≥ 90% with 5000 bp). Detailed viral analysis can be found in the Supplementary Methods.

### Amplicon sequencing

The effects of climate change on soil microbial community structure were corroborated by amplicon sequencing of 16 S/18 S rRNA genes and ITS1 regions using the same DNA as for metagenomics, from the IG treatment in 2015 and 2018 summers (20 samples, 2 climates × 5 replicates × 2 summers). Detailed amplicon analysis can be found in the Supplementary Methods.

### Soil metatranscriptomics in 2022

To investigate the expression levels of marker genes and MAGs, a total of 24 soil samples (2 climates × 6 replicates × 2 months) were taken from CF plots in May and July 2022. The soil samples were immediately frozen in liquid nitrogen in the field. After quality control and filtering of rRNA reads, the potential mRNA reads were mapped to the representative MAGs and non-redundant genes using CoverM, and the expression profiles were then normalized to RPKM. The RNA/DNA ratio was calculated by dividing the RPKM of metagenomic genes (CF average 2014–2019) by the RPKM of metatranscriptomic genes (CF 2022). Furthermore, the soil RNA virome in 2022 was identified based on the viral hallmark gene RNA dependent RNA polymerase (RdRP) [44]. Detailed metatranscriptomic analysis can be found in the Supplementary Methods.

### Statistical analysis

To assess the impact of climate, land-use, year, and their interactions on soil properties and alpha-diversity, we applied linear mixed-effects models (LMMs) with random intercepts and nested sampling time points [25] using the lme4 v1.1 [45] and lmerTest v3.1 [46] packages in R. The emmeans v1.7.2 [47] package was used to extract contrasts and interactions, followed by Tukey’s HSD test. We conducted a permutational analysis of variance (PERMANOVA) using the ‘adonis’ function from the vegan package to examine differences in community structure and function between treatments. The correlations between Bray–Curtis dissimilarity of microbial traits (taxonomy and function) and Euclidean distances of environmental variables were assessed using the Mantel test with the ggcor package in R.

To assess the relative importance of ecological processes in microbial community assembly, the phylogenetic bin-based null model was applied using the iCAMP v1.5.12 package [48]. Specifically, we extracted the V3-V4 regions of the 16 S rRNA gene from metagenomic contigs and clustered them into OTUs. These OTUs were then categorized into different ‘bins’ based on their phylogenetic relationships using the RAxML. The significance of difference in the relative contributions of ecological process was determined by computing the standardized effect size (Cohen’s *d*) and tested by bootstrapping with 1000 repetitions. Cohen’s *d* was calculated as the difference of means between treatments divided by the combined standard deviation. Soft clustering of genes was performed using the Mfuzz v2.50.0 package [49]. Detailed information on the iCAMP and Mfuzz analyses can be found in the Supplementary Methods. To identify significant changes in taxonomic and functional matrices, we used DESeq2 v1.30.1 with the formula (design = ~Climate + Land-use + Year) and default fitType [50]. The resulting *p* values were adjusted for multiple testing using the Benjamini and Hochberg (BH) method.

## Results

### Impact of simulated climate and extreme summers on soil properties

**Fig. 1:**
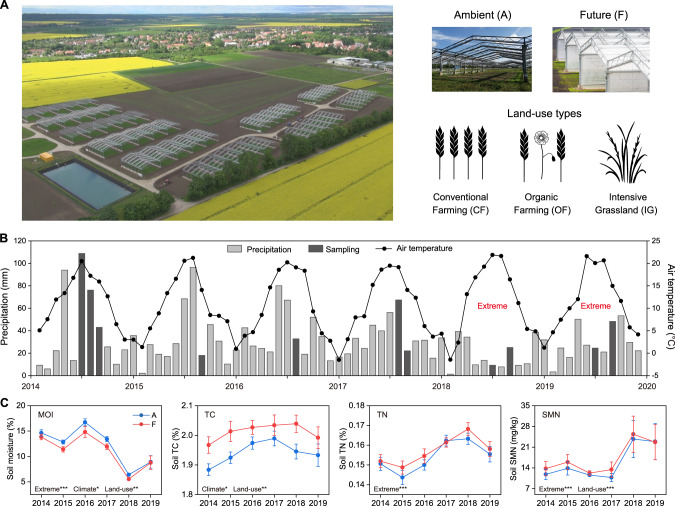
GCEF, weather, and soil conditions during the summers of 2014–2019. **A** Aerial photo of the GCEF experimental site in Bad Lauchstädt, Germany. Experimental design: two climate treatments (A and F) and three land-use types (CF, OF, and IG). **B** Monthly ambient precipitation and air temperature during the experiment. **C** Physicochemical properties of surface soil samples. Error bars represent the standard error of the mean. Significant effects of extreme summers, climate, and land-use changes on the soil properties were determined using linear mixed-effects models, significance levels: *p < 0.05; **p < 0.01; ***p < 0.001. GCEF Global Change Experimental Facility, CF conventional farming, OF organic farming, IG intensive grassland, MOI soil moisture, TC total carbon, TN total nitrogen, SMN soil mineral nitrogen. Photo: Tricklabor/Service Drohne.

During the 2018 growing season (April–September), the average temperature was 2.2 °C higher than the mean of 2014–2017. In addition, there was a notable decrease in rainfall of about 65% at the study site (Fig. 1). In the following summer of 2019, the area experienced another extreme drought with a 45% reduction in rainfall. As a result, the average water content of soil samples collected from 2014 to 2019 was 14.2%, 12.1%, 15.8%, 12.7%, 6.0%, and 8.9% (Table S2). The LMMs testing showed that future climate significantly increased total soil C content and decreased soil moisture (Fig. S3, Table S3). In extreme summers (2018–2019), soil total N content was significantly increased compared to normal summers (2014–2017) (Fig. S3, Table S3).

### Impact of simulated climate and extreme summers on soil microbial community structure and assembly

**Fig. 2:**
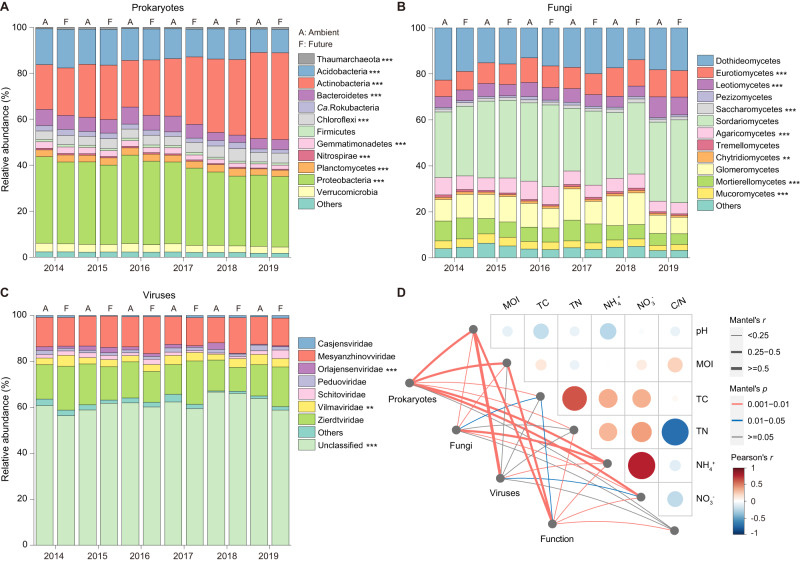
Impact of climate change on the composition of soil microbiomes during the summers of 2014–2019. **A**, **B** Bar plots depict the proportion of major phyla in prokaryotic and fungal communities based on metagenomic reads, respectively. **C** Bar plot showing the proportion of major viral families in soil samples. Taxonomic groups with a relative abundance <0.5% were combined into “Others”. Asterisks indicate significant differences between normal and extreme summers based on DESeq2 (*p < 0.05; **p < 0.01; ***p < 0.001). **D** Mantel’s correlation analysis between soil properties and soil microbial communities. The Mantel test was performed at genus level (archaea, bacteria, and fungi), vOTU (DNA viruses), and KEGG KO (function) level, respectively. Pearson’s correlations were calculated between soil properties.

A total of 8.64 Tb of FASTQ reads were generated from 180 metagenomes, with an average of 48 Gb per sample (Table S4). Taxonomic analysis based on the FASTQ reads (Fig. 2), contigs, and SSU rRNA genes (Fig. S4) revealed similar patterns in the major phyla affected by simulated climate and extreme summers. Extreme summers significantly impacted the composition of soil microbial communities, resulting in an increased relative abundance of Actinobacteria and a decreased abundance of Acidobacteria, Bacteroidetes, and Proteobacteria (DESeq2, *p* < 0.001) (Fig. 2A, Figs. S5–S8). The microbial Shannon index was strongly reduced in extreme summers (Fig. S9, Table S3). PCoA analysis demonstrated a separation of prokaryotic communities between normal and extreme summers (Fig. S10, Table S5). In contrast, future climate scenarios caused similar shifts in community composition, but to a lesser extent compared to extreme summers. Thaumarchaeota consistently exhibited a higher relative abundance under future climate across different analysis methods (Figs. S4, S11). Furthermore, Actinobacteria showed a significantly higher relative abundance (~ + 21%) in grassland compared to cropland (*p* < 0.001) (Fig. S6).

The taxonomic analysis of SSU rRNA genes obtained from metagenomics revealed that the fungi accounted for ~2.9% of classified sequences (Fig. S4). At order level, extreme summers resulted in a significant increase in the relative abundance of Eurotiales (class *Eurotiomycetes*), while future climate scenarios led to an increased abundance of Hypocreales (class *Sordariomycetes*) (Fig. 2B, Figs. S12–S15). Furthermore, the relative abundance of arbuscular mycorrhizal fungi (AMF) (class *Glomeromycetes*) was significantly higher in grassland than in cropland. The trends observed in bacterial and fungal community compositions caused by simulated climate and extreme summers were supported by amplicon sequencing of 16 S/18 S rRNA genes and ITS1 sequences from the IG treatment (2015 vs. 2018) (Figs. S16–S17).

**Fig. 3:**
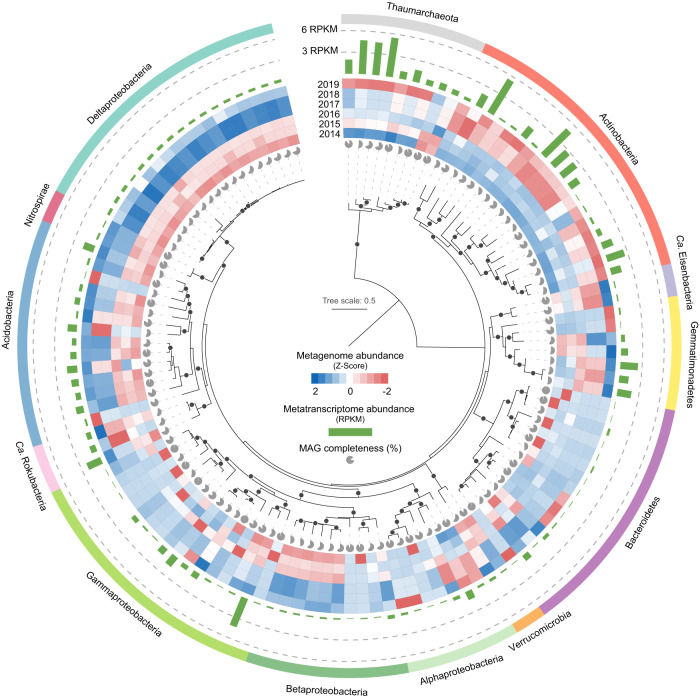
Phylogenetic placement of dereplicated MAGs from this study. The maximum likelihood tree of MAGs was constructed using RAxML based on conserved marker genes in CheckM. Bootstrap values > 90% are shown. Taxonomic classification of the MAGs was inferred using GTDB-tk. The heatmap indicates the abundance of MAGs in metagenomics (RPKM) during the summers of 2014–2019. The bar chart shows the abundance of MAGs in metatranscriptomics (RPKM) in July 2022. The completeness of MAGs is represented by the pies at the branch tips.

The assembly and binning of metagenomic data yielded 751 MAGs with completeness >50% and contamination <10% (Table S6). After dereplication, 122 dereplicated MAGs (113 bacteria and 9 archaea) were obtained, and less than half of these MAGs were enriched in extreme summers, mainly belonging to Actinobacteria and Thaumarchaeota (Thermoproteota in GTDB taxonomy) (Fig. 3). Furthermore, based on metatranscriptomic mapping, Actinobacteria and Thaumarchaeota MAGs were highly active during the summer of 2022.

We identified 3,735 putative viral DNA contigs >5 kb (including 1767 contigs > 10 kb) and clustered them into 2524 vOTUs. Taxonomic analysis showed that the *Siphoviridae* family was the most dominant (on average 65.0%) in all soils (Fig. S18A). According to the International Committee on Taxonomy of Viruses (ICTV) classification, extreme summers significantly increased the relative abundance of the *Vilmaviridae* family (*p* < 0.01) (Fig. 2C, Fig. S18B). We characterized 115 unique host-virus linkages between 78 vOTUs and 61 MAGs, and certain vOTUs exhibited strong correlations with linked MAGs, such as Nitrososphaerales (Pearson’s *r* = 0.67), Acidobacteriales (*r* = 0.91), Pyrinomonadales (*r* = 0.75), and Propionibacteriales (*r* = 0.62) (Figs. S19–S20). Furthermore, we identified 4139 putative RNA viral contigs (>300 bp) and clustered them into 2281 RNA vOTUs. The taxonomic assignment of RNA vOTUs showed that *Fiersviridae*, *Mitoviridae*, and *Endornaviridae* families were dominant groups (relative abundance > 5%) (Fig. S18C). Statistical analysis indicated that both the sampling season and soil pH significantly impacted the community structure of RNA viruses (Fig. S18D).

**Fig. 4:**
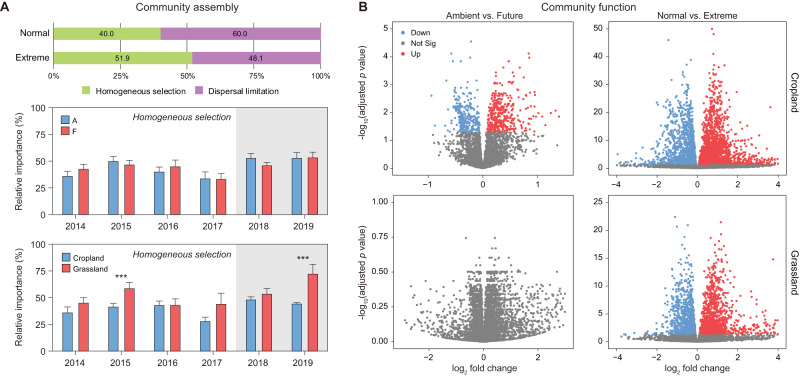
Impact of climate and land-use changes on the microbial community assembly and function in cropland and grassland. **A** Changes of homogeneous selection to bacterial community assembly were estimated by iCAMP. Data are presented as mean values ± SD. Error bars represented standard deviations (*n* = 15, 20, and 10 biologically independent samples for A/F, cropland, and grassland treatment, respectively). One-side significance based on bootstrapping test is expressed as ***p < 0.01. **B** Volcano plots depicting the abundances of genes (KEGG KO) that exhibit significant differences between the compared groups. Significantly altered genes are marked in red (up) and blue (down) based on DESeq2 *p* < 0.05.

The correlation analysis revealed that soil pH significantly impacted the community structure of prokaryotes and viruses, whereas soil ammonium content significantly impacted the fungal community (Fig. 2D). The findings of iCAMP showed that homogeneous selection and dispersal limitation were the major drivers of bacterial community assembly. In extreme summers, homogeneous selection played a more important role compared to normal summers (Fig. 4A). The contribution of homogeneous selection to bacterial community assembly increased significantly (*p* < 0.001) from 40.0% in normal summers to 51.9% in extreme summers. Furthermore, the homogeneous selection differed significantly between cropland and grassland (Cohen’s *d* = −5.87, *p* < 0.001), but not between ambient and future climate (Cohen’s *d* = 0.04, *p* = 0.49).

### Impact of simulated climate and extreme summers on soil microbial pathways

In extreme summers, approximately half of the major KEGG pathways, including amino acid, carbohydrate, cofactors and vitamins, and nucleotide metabolisms, showed enrichment (Figs. S21–S22). Differential abundance analysis identified 3052 and 690 significantly (DESeq2, *p* < 0.05) affected KOs in cropland due to extreme summers and simulated climate, respectively (Fig. 4B). However, no significant impact of simulated climate on microbial functional profiles was observed in grassland (PERMANOVA, *R*^*2*^ = 0.025, *p* = 0.060). The PCoA plot indicated a separation of microbial functional profiles between normal and extreme summers (Fig. S23).

**Fig. 5:**
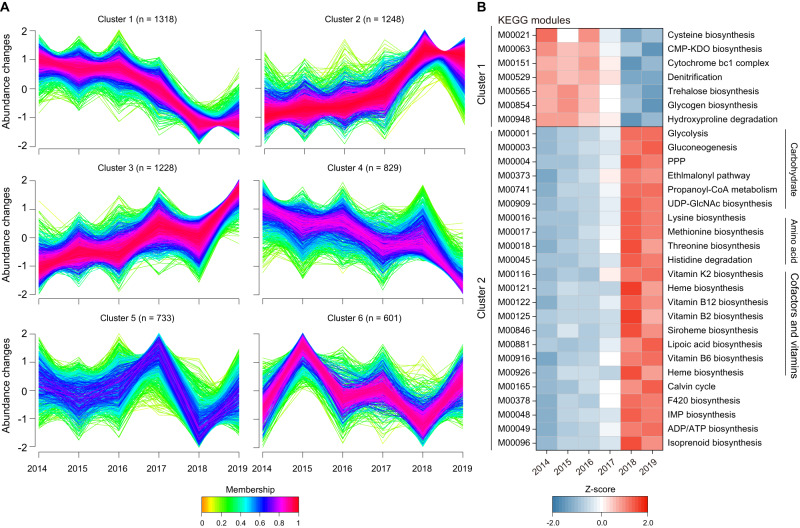
Cluster analysis of gene abundance profiles during the summers of 2014–2019. **A** Six clusters show the abundance patterns of KEGG KOs across 6 years and the number of KOs in each cluster is displayed. **B** Heatmap showing KEGG modules (completeness > 60%) based on KOs in Cluster 1 and 2 (membership > 0.5). The RPKM value of each module was log_10_ transformed before row scaling. CMP-KDO cytidine 5′-monophospho-3-deoxy-d-manno-2-octulosonic acid, PPP pentose phosphate pathway, UDP-GlcNAc uridine diphosphate N-acetylglucosamine, IMP inosine monophosphate, ADP adenosine diphosphate, ATP adenosine triphosphate.

Cluster analysis of KEGG KOs identified six distinct clusters. Cluster 1 showed a negative impact, while Cluster 2 exhibited a positive impact of extreme summers (Fig. 5A, Table S7). Cluster 1 was associated with KEGG modules related to trehalose biosynthesis and denitrification pathways, whereas Cluster 2 was enriched with KEGG modules associated with amino acid, carbohydrate, cofactors and vitamins metabolisms. Taxonomic analysis of Cluster 2 modules revealed an increased abundance of Actinobacteria and Chloroflexi in extreme summers (Fig. S24).

### Impact of simulated climate and extreme summers on microbial functions

**Fig. 6:**
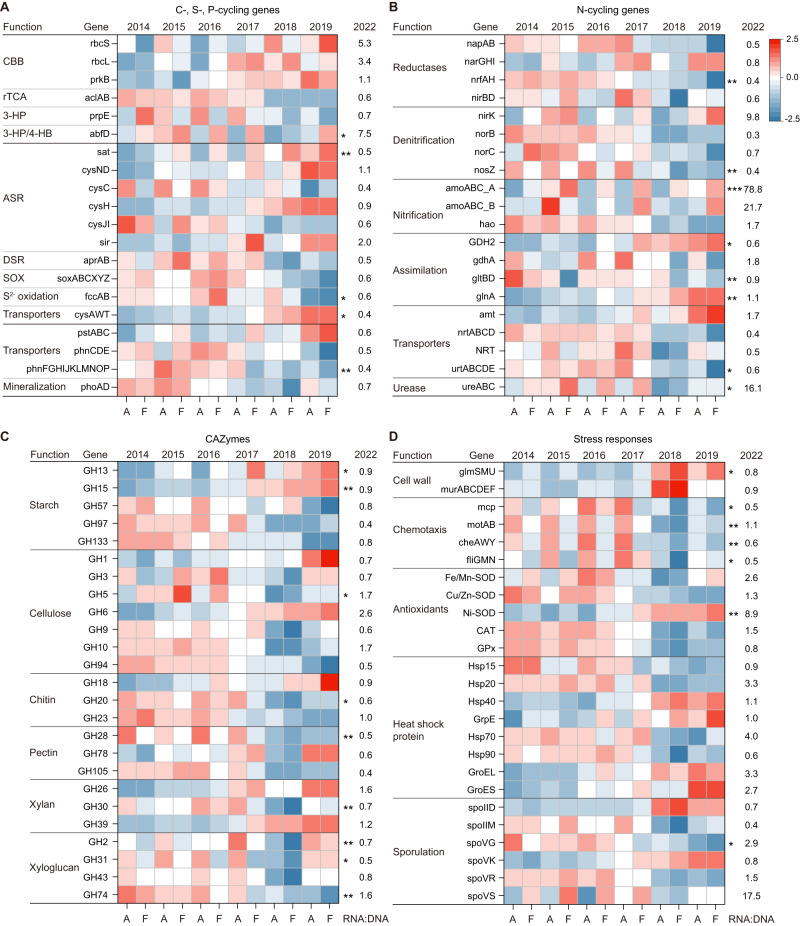
Impact of climate change on genes involved in microbial biogeochemical cycles and stress response. Heatmaps displaying abundances of genes related to C, S, and P cycles (**A**), N cycle (**B**), CAZymes (**C**), and stress response (**D**) that were affected by climate change. Asterisks indicate significant differences between A and F treatments over 6 years based on DESeq2 (*p < 0.05; **p < 0.01; ***p < 0.001). The RPKM value of genes was log_10_ transformed before row scaling. The RNA/DNA ratio of selected genes was calculated using metagenomics and metatranscriptomics. CBB Calvin–Benson–Bassham, rTCA reductive tricarboxylic acid, 3-HP 3-hydroxypropionate, 3-HP/4-HB 3-hydroxypropionate/4-hydroxybutyrate, ASR assimilatory sulfate reduction, DSR dissimilatory sulfate reduction, SOX sulfur oxidizing, SOD superoxide dismutase, CAT catalase, Hsp heat shock protein, GPx glutathione peroxidase.

We additionally examined microbial functional traits related to C, N, S, P cycling, and stress resistance using marker genes in all soils (Fig. 6, Appendix 1 and 2) and across different land-use types (Figs. S25–S28). Our results showed that extreme summers significantly impacted the relative abundances of surveyed genes (DESeq2, *p* < 0.05). Specifically, for C cycling, the relative abundances of genes involved in CO_2_ fixation (CBB cycle) and starch degradation (GH13 and GH15) were significantly increased in extreme summers. For N cycling, the abundances of genes encoding glutamine synthetase (*glnA*) and glutamate dehydrogenase (*GDH2*) were significantly higher in extreme summers. For S cycling, genes related to ASR showed a significant increase in abundance, while genes related to DSR and SOX displayed strong reductions. For P cycling, phosphonate uptake and mineralization of organic phosphorus were reduced in extreme summers. The simulated climate also affected the abundance of *nosZ*, *amoABC*, and *glnA* genes. Furthermore, metatranscriptomic mapping revealed relatively high expression levels of archaeal *amoABC* genes in 2022 summer.

In stress response systems, the abundances of peptidoglycan biogenesis genes *glmSMU* and *murABCDEF* were significantly higher in extreme summers (Fig. 6D). Conversely, the abundances of key genes involved in chemotaxis were significantly decreased. The relative abundances of antioxidant genes were drastically reduced, except for the nickel (Ni)-dependent SOD. The abundance of GroES/GroEL and Hsp40 chaperonin complex genes, as well as two sporulation-related genes (*spoIID* and *spoVK*), were significantly increased in extreme summers. Taxonomic analysis showed that the enriched genes were mainly encoded by Acidobacteria, Actinobacteria, and Proteobacteria (Fig. S29). However, the Ni-SOD genes were predominantly contributed by Actinobacteria and Chloroflexi (~97%) (Fig. S30), and their transcriptional activities were relatively high in 2022 summer.

### Impact of simulated climate and extreme summers on microbial secondary metabolism

**Fig. 7:**
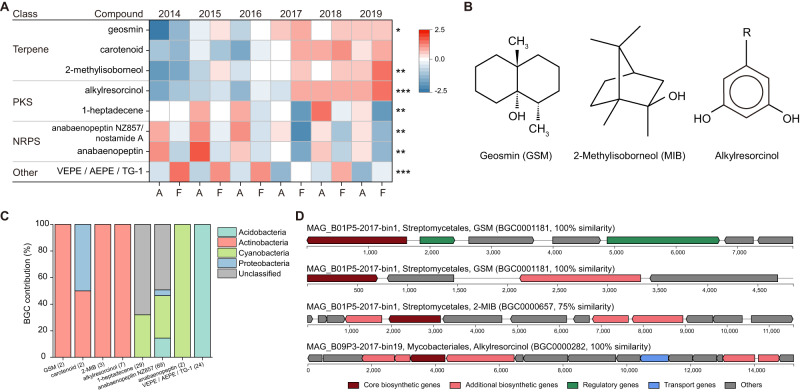
Impact of climate change on microbial BGCs during the summers of 2014–2019. **A** Heatmaps showing abundances (>0.5%) of BGCs in metagenomic contigs significantly affected by extreme summers. The RPKM value of BGCs was log_10_ transformed before row scaling. Asterisks indicate significant differences between A and F treatments based on the DESeq2 (*p < 0.05; **p < 0.01; ***p < 0.001). **B** Chemical structure of geosmin (GSM), 2-methylisoborneo (MIB), and alkylresorcinols. R represents alkyl side chains. **C** Phylum-level assignment of BGC-containing contigs. Numbers in brackets represent the number of contigs assigned to the same BGC. **D** BGCs in assembled MAGs as predicted by antiSMASH. The sequence similarities between query and reference BGCs in the MIBiG database are shown in brackets. PKS polyketide synthase, NRPS nonribosomal peptide synthetase.

We identified a total of 226 contigs (including 152 contigs with 100% similarity) that contained BGCs from the MIBiG database, with biosynthetic products belonging to NRP (127), NRP-PKS (19), PKS (42), terpene synthesis (11), and other (27) classes (Fig. 7A, Figs. S31–S32). The relative abundances of GSM, 2-MIB, and alkylresorcinol BGCs were significantly increased in extreme summers and were exclusively contributed by Actinobacteria (Fig. 7C). Further analysis of the MAGs revealed that 7.5% (56/751) of them encoded at least one BGC (Table S8). Among these MAGs, three terpene clusters were found in one MAG classified as *Streptomycetales* and one PKS in one MAG classified as *Mycobacteriales* (Fig. 7D).

## Discussion

During the extreme summer of 2018, the soil water content was only around 6%, indicating substantial changes in soil microbiomes to adapt to such conditions. Our previous study reported a significant reduction in soil microbial activity and biomass during the years 2018–2019 compared to 2015–2017 [25]. In addition, a decrease in soil moisture by ~45% has been shown to reduce the activity of carbon cycling enzymes in GCEF soils [51]. Our multi-omics study supports these findings, revealing a significant increase in the abundance of Actinobacteria and Thaumarchaeota (mainly *Nitrososphaera*) under warming and drought conditions [52–55]. This aligns with prior research showing a greater relative abundance of Actinobacteria and Thaumarchaeota in Austrian grasslands subjected to repeated droughts over a decade [56]. The metatranscriptomic mapping of *amoABC* genes and MAGs provided additional evidence of the activity of Thaumarchaeota. Thaumarchaeota are recognized for their ability to oxidize ammonia and are widely distributed in terrestrial and aquatic ecosystems [57, 58]. Their enrichment during frequent droughts may reduce soil ammonium availability [56], potentially impacting ecosystem productivity and plant growth in the future.

Compared to prokaryotes, our understanding of soil viruses and their response to climate change is relatively limited. However, recent studies have provided insights into the important roles of soil viruses in organic matter decomposition [59, 60], methane oxidation [61], and pesticide degradation [62]. We found that extreme summers significantly affected the community structure of soil DNA viruses, and observed a strong correlation between virus abundance and their hosts, particularly Actinobacteria and Acidobacteria. This finding is in line with previous studies highlighting soil moisture as a key factor influencing soil viral abundance and composition [63, 64]. In addition, soil pH and land-use types were significant factors shaping the community structure of soil DNA viruses [65]. Soil pH is a critical determinant of global soil bacterial communities [66], and may directly influence virus-host dynamics.

Recent metatranscriptomic studies have revealed the presence of a diverse and abundant RNA viruses in grassland soils [67, 68]. Consistent with previous studies [67, 69, 70], the most abundant RNA viruses in GCEF soils were affiliated with the family *Fiersviridae* (formerly named *Leviviridae*), which primarily infect prokaryotes. Several eukaryotic RNA viruses (e.g., *Mitoviridae*, *Endornaviridae*) were also detected, with fungi, protists and plants serving as their natural hosts [9]. Our findings indicate that the community structure of prokaryotic RNA viruses was significantly affected by soil pH, while eukaryotic RNA viruses were significantly affected by sampling season. These findings suggest that both soil DNA and RNA viruses are sensitive to changes in soil pH and climate change, which may affect ecosystem function through host-mediated mechanisms. Viruses have been shown to impact C cycling by harboring and expressing auxiliary metabolic genes (AMGs) [59, 60]. Further virus enrichment and annotation of AMGs would greatly enhance our understanding of their ecological consequences [22].

The impact of climate change on soil fungi can vary depending on the level of soil moisture and vegetation at different study sites [9]. Our study revealed that the fungal community was less affected by simulated climate change compared to prokaryotes. However, we did observe significant increases in the abundance of Hypocreales and Eurotiales under simulated climate and extreme summer conditions, respectively. These groups are known to harbor many soil-borne plant pathogens [71]. Previous GCEF studies showed that simulated climate significantly altered the community structure of pathogenic fungi in plant residues and introduced new fungal pathogens into the ecosystem [72, 73]. Climate warming may also lead to an increase in soil-borne fungal pathogens [18, 19], which can have negative implications for crop yield and quality. At the GCEF, the biomass of AMF was significantly influenced by land-use types but not by future climate scenarios [25]. Our results showed a relatively high abundance of AMF in 2018 across land-use types, suggesting that AMF symbiosis may confer drought tolerance [74]. However, additional research is required to determine whether AMF colonization provides protection against infections by pathogenic fungi and viruses under climate change scenarios [75].

In extreme summers, we observed increased gene abundance in key metabolic pathways, including amino acid metabolism, cofactors and vitamins metabolism, and glycolysis. CAZymes related to easily degradable starch were also significantly increased, indicating a high energy demand. The enhanced biosynthesis of amino acids, particularly lysine and methionine, may contribute to microbial stress tolerance [76, 77]. The increase in genes associated with sulfur-containing methionine biosynthesis corresponded to the higher abundance of assimilatory sulfate reduction. Moreover, we found that genes related to N assimilation were significantly enriched in extreme summers. Glutamine and glutamate, important N intermediates, are likely to serve as compatible solutes for microbes under drought stress [78]. Our analysis of Actinobacteria MAGs and reference genomes confirmed the presence of *GDH2* genes and multiple *glnA* homologs (Table S9), highlighting the potential role of nitrogen assimilation in the drought tolerance of Actinobacteria (For further discussion, see Supplementary information).

Under stressful conditions, microbiomes have developed diverse stress response systems to maintain their functionality. Previous studies have indicated that a thick peptidoglycan cell wall and sporulation can assist bacteria, especially Gram-positive species, in tolerating drought [79, 80]. In our study, we observed a significant increase in the relative abundance of genes involved in cell wall peptidoglycan biosynthesis (*glmSMU* and *murABCDEF*) in extreme summers [81]. However, only a few sporulation genes (*spoIID* and *spoVK*) showed enrichment, suggesting that sporulation may not be a universal strategy for microbes to cope with drought. HSPs, such as GroES/GroEL and Hsp40 chaperonins, known for their role in cellular protection under stress [82], were specifically enriched in extreme summers. Despite chemotaxis enabling bacteria to move toward nutrients and sense environmental changes [83], we observed a significant reduction in gene abundance under drought conditions.

Extreme drought can induce oxidative stress in microbial cells by producing reactive oxygen species, which can cause irreversible damage or cell death [84]. Antioxidants, such as SOD, CAT, and GPx, play a crucial role in microbial defense mechanisms and survival strategies [85]. However, in extreme summers, the relative abundances of these antioxidant genes were significantly reduced, except for Ni-SOD. The majority of Ni-SOD genes were found in Actinobacteria and Chloroflexi. Previous research has shown that Ni-SOD is present in a limited range of Actinobacteria and marine Cyanobacteria strains [86]. Moreover, the transcriptional activity of Ni-SOD was relatively high in 2022. Further research is needed to explore the relationship between soil nickel availability and the drought tolerance of Actinobacteria.

Actinobacteria are well-known for their capacity to produce antibiotics and emit an “earthy” smell due to the biosynthesis of terpenoids such as geosmin and 2-MIB [87]. The enrichment of geosmin and 2-MIB related BGCs in extreme summers suggests that these volatiles may confer selective advantages for Actinobacteria. Geosmin can serve as a warning signal indicating the unpalatability of its producers and discouraging predation by bacteriophagous nematodes [88]. Furthermore, geosmin and 2-MIB production has been shown to facilitate the dispersal of *Streptomyces* spores by springtails in soil [89]. In addition, there was a significant increase in the abundance of alkylresorcinol-related BGCs in extreme summers. Consistent with our analysis of MAGs, the biosynthesis of alkylresorcinols has been confirmed in pathogenic *Mycobacterium* [90]. Alkylresorcinols have demonstrated antimicrobial properties by disrupting cellular membranes [91]. Therefore, it is of particular interest to investigate the roles of these volatile compounds and antibiotics produced by Actinobacteria under climate change.

The iCAMP analysis of metagenomics revealed that dispersal limitation and homogeneous selection were the dominant processes underlying bacterial community assembly. Particularly, homogeneous selection played a larger role during naturally occurring extreme summers. Similarly, a previous study conducted in a grassland in the USA showed that a 5-year soil warming of 2–3 °C enhanced homogeneous selection and decreased drift in bacterial community assembly [48]. In normal summers, the community experienced less selection pressure, and stochastic processes governed the assembly. The impact of land-use types on microbial community assembly has been recognized [92, 93]. However, no significant difference was found in the bacterial community assembly processes between ambient and future climate conditions, which is in line with previous observations at the GCEF [25, 94].

Our results suggest that grassland microbiomes are more resistant to future climate scenarios compared to cropland microbiomes. While fungal-based food webs in grassland are generally considered more adapted to drought than bacterial-based food webs in cropland [95], our findings suggest that the higher abundance of Actinobacteria (+21%) in grassland may contribute to its enhanced drought tolerance. Previous research conducted at the GCEF has shown a significantly higher biomass (+40%) of Gram-positive bacteria in grasslands compared to croplands, with no significant difference observed for AMF [25]. The conversion of grassland to cropland over the past decades has resulted in a decline in soil carbon pools [96], which can impact soil functioning and its resilience to climate change. In addition, it is important to consider the potential influences of fertilizer application and plant rotation practices on the observed changes in soil microbiomes, although our primarily focus was on the effects of extreme summers.

In conclusion, our study highlights the significant impact of extreme summers in Central Europe during 2018–2019 on the community structure, assembly, and function of soil microbiomes in cropland and grassland. Land-use type proved to be a more influential factor than future climate scenarios in shaping soil microbial communities. Our findings emphasize the importance of further investigating the responses of ammonia-oxidizing archaea and soil DNA and RNA viruses to climate changes. We also identified microbial anti-stress mechanisms, such as cell wall biosynthesis, HSPs, and Ni-SOD antioxidants, which may play a significant role in mitigating the effects of extreme summers. In addition, our analysis of biosynthetic gene clusters revealed significant effects of extreme summers on the production of secondary metabolites, including geosmin and 2-MIB, with potential implications for water quality [97]. Overall, our study provides a comprehensive assessment of soil microbiomes in response to extreme summers, offering valuable insights into their dynamics and functioning.

## Supplementary information

Supplemental_Figures

Supplementary-Tables

## Data Availability

All data supporting the findings of this study are available within the paper and its Supplementary Information. The sequences reported in this paper have been deposited in the National Center for Biotechnology Information Sequence Read Archive (PRJNA838942, metagenomics; PRJNA903142, metatranscriptomics; PRJNA902961, amplicon sequencing). All MAGs generated from the soil metagenomics can be accessed at FigShare (DOI: 10.6084/m9.figshare.20260221).
