# Supplementary material for: Extreme summers impact cropland and grassland soil microbiomes
Source: ISME J. 2023 Jul 7;17(10):1589–600. doi: 10.1038/s41396-023-01470-5 (PMC10504347; doi:10.1038/s41396-023-01470-5)

**Supplementary Information for**

**Extreme summers impact cropland and grassland soil microbiomes**

Qicheng Bei^1, 2, *^, Thomas Reitz^1,2^, Beatrix Schnabel^2^, Nico Eisenhauer^1,3^, Martin Schädler^1,4^, François Buscot^1,2^, Anna Heintz-Buschart^5, *^

**This pdf file includes:**

Supplementary Material and Methods

Supplementary Discussion

Supplementary Fig. S1 to S34

Supplementary Table. S1, S3 and S5

Appendix 1: Function of selected KEGG Orthologs (KOs)

Appendix 2: Activity of selected glycoside hydrolases (GHs)

Appendix 3: An overview of the bioinformatics pipeline

**Supplementary Material and Methods**

### *GCEF* *experimental design*

### The GCEF research station comprises 50 plots, each spanning an area of 400 m^2^, arranged in a split-plot design. The climate regime (ambient vs. future) was the main-plot factor, and the subplot factor was five land-use types (Fig. S1). The subplots were randomly assigned to one of the following land-use regimes: (1) conventional farming (CF), (2) organic farming (OF), (3) intensively-used grassland (IG), (4) extensively-used meadow (EM), and (5) extensively-used pasture (EP). The future climate regime represents a consensus scenario across three models (COSMO-CLM [1], REMO [2], and RCAO [3]) of climate change in Central Germany for the year between 2070 and 2100. To simulate the future climate, the future climate plots were equipped with mobile shelters, side panels, and an irrigation system, with the roofs controlled by a rain sensor. The shelters and panels automatically close from sunset to sunrise, resulting in an increase of the mean daily air temperature by 0.55 ℃ at a height of 5 cm and the mean daily soil temperature by 0.62 ℃ at a depth of 1 cm and 0.50 ℃ at a depth of 15 cm [4]. The ambient climate plots are equipped with the same steel constructions but without shelters, panels, and irrigation systems, to mimic possible microclimate effects of the experimental setup. The use of mobile roofs and irrigation systems reduces the rainfall by ~20% in summer and increases the rainfall by ~10% in spring and autumn [5].

***Soil property analysis***

Soil pH was determined by suspending 12 g of air-dried soil in 30 ml of 0.01 M CaCl_2_ and measuring it using a pH electrode (InLab Expert Pro-ISM, Mettler-Toledo) after incubation for 1 hour. Soil mineral nitrogen was extracted from 5 g fresh soil using 20 ml of 1 M KCl and determined by flow injection analysis (FlAstar 5000, Foss GmbH). Total organic carbon and total nitrogen content were measured by dry combustion with a Vario EL III C/H/N analyzer (Elementar).

### *Analysis of microbial community composition*

Taxonomic classification of metagenomics was assessed based on various datasets, including FASTQ reads, contigs, SSU rRNA genes, and MAGs (Fig. S4). For the FASTQ reads and contigs, taxonomy was determined by Kaiju v1.8.2 [6] using the NCBI nr_euk database (https://kaiju.binf.ku.dk/server). The SSU rRNA genes were extracted from metagenomics using SortMeRNA v2.1 [7] and searched against the SILVA SSU database v138.1 [8] using BLASTn (-e 1e-5 -qcov_hsp_perc 80). The alpha diversity of metagenomics was calculated using operational taxonomy units (OTUs) defined with SingleM v0.13.2 based on the *rplB* gene, clustered at 97% identity [9]. The principal coordinate analysis (PCoA) and Shannon index were calculated using the phyloseq v1.34.0 package [10] in R v4.0.5. For fungi, the taxonomic assignments based on the FASTQ reads were preferred due to their higher information content (27,430 to 236,331 hits per sample) at class and order levels.

### *Analysis of soil DNA viruses*

Viral contigs from metagenomics were filtered from the metagenomic assemblies using VirSorter2 v2.2.3 [11] and CheckV v1.0.1 [12] based on the previously described protocols [13-15]. Contigs that were greater than 5 kb in length and met the following criteria were retained: (1) a VirSorter2 score ≥ 0.9; (2) containing at least one hallmark gene from VirSorter2; and (3) containing at least one viral gene from CheckV. The identified contigs from each assembly were then compiled and clustered at 95% nucleotide identity using CD-HIT v4.8.1 [16] (-c 0.95 -n 10 -aS 0.9). To assess the relative abundance of virus operational taxonomic units (vOTUs) in 180 samples, metagenomic reads were mapped to the vOTUs using CoverM v0.6.1, with a minimum sequence identity of 95% and query coverage of 90% [17].

To identify the taxonomic affiliations of vOTUs, Prodigal-predicted ORFs were aligned against the NCBI Viral RefSeq database (August 2022) using DIAMOND v2.0.15 [18] (BLASTp, -e 1e-5 --id 30). The resulting output was then imported into MEGAN v6.24.1 [19] with default parameters for taxonomic analysis. Taxonomy was assigned to vOTUs if more than 50% of the proteins within the vOTU aligned to the same family with a bitscore ≥ 50 [20]. Furthermore, the vOTUs were annotated using PhaGCN [21] in accordance with the viral classification system of the International Committee on Taxonomy of Viruses (ICTV) [22].

The vOTUs and MAGs were further linked through clustered regularly interspaced short palindromic repeats (CRISPR) spacers and shared genomic content using previously described methods [23, 24]. CRISPR spacers were detected in the MAGs using MinCED v0.4.2 (-minNR 2 -spacers) (https://github.com/ctSkennerton/minced) and linked to the vOTUs with BLASTn v2.9.0 (-dust no -word_size 7 -e 1e-5). Only BLASTn hits with a query coverage ≥ 95% and ≤ 1 mismatch were retained. In addition, FastANI v.1.33 [25] was used to link vOTUs and MAGs with average nucleotide identity (ANI) ≥ 90% based on 5000 bp fragment [24]. To perform linear regression analysis [17, 23], the virus-host abundance ratio was calculated based on the RPKM values of vOTUs and MAGs.

### *Amplicon sequencing*

The microbial community composition was determined by sequencing 16S/18S rRNA gene and ITS1 amplicons using soil DNA samples from the intensive grassland (IG) treatment in the 2015 and 2018 summers, resulting in a total of 20 soil samples. The 16S rRNA gene was amplified using the primer set 515F/806R [26, 27], the fungal ITS1 region was amplified using the primer set ITS1F/ITS2 [28], and the V9 region of the 18S rRNA gene was amplified using the primer set 1391F/1510R [29, 30]. The PCR amplifications of bacterial and fungal amplicons were performed using protocols suggested by the Earth Microbiome Project (EMP) [31]. The paired-end sequencing of 2 × 300 bp was performed on the MiSeq platform (Illumina, San Diego, CA, United States) at the NGS Competence Center Tübingen (NCCT).

After quality control, we obtained a total of 1,666,126 16S rRNA gene sequences, 1,708,127 18S rRNA gene sequences, and 1,543,374 fungal ITS sequences from the 20 soil samples. The paired-end reads were merged and clustered into operational taxonomic units (OTUs) at a 97% identity threshold using USEARCH v11 [32]. To obtain taxonomic information of the OTUs, representative 16S (> 95% identity) and 18S (> 90% identity) rRNA genes were assigned using the SILVA database v138 (<https://www.arb-silva.de/aligner/>) (May 2023). The ITS2 sequences were aligned against the UNITE databases using the RDP classifier v2.13, respectively (<http://rdp.cme.msu.edu>). OTUs assigned to the unknown, chloroplast or mitochondria were excluded from the downstream analysis.

### *Soil* *metatranscriptomics in 2022*

Soil samples were collected from conventional farming (CF) treatment in May and July 2022, at a depth of 0-10 cm. A total of 24 soil samples were collected from ambient and future plots in the two sampling campaigns. Visible plant residues and stones were removed, and the homogenized soil was immediately frozen in liquid nitrogen in the field. Total soil RNA was extracted using the RNeasy PowerSoil total RNA kit (Qiagen). Metatranscriptomic library preparation was performed in our laboratory as previously described [33]. Genomic DNA was removed from the total RNA using DNase (Turbo DNase, Ambion) and the RNA was then purified using the RNA Clean & Concentrator-25 Kit (Zymo Research). The cDNA libraries were generated using the NEB Next Ultra Directional RNA Library Prep Kit for Illumina (New England BioLabs). The quality of cDNA library was assessed using the Agilent 2100 Bioanalyzer System with High Sensitivity DNA reagents and DNA chips (Agilent Technologies). The metatranscriptomic sequencing was performed by Novogene (UK) using the Illumina NovaSeq platform (2 × 150 bp). The raw sequence data were quality filtered using Trimmomatic v3.6 [34] and rRNA reads were removed using SortMeRNA v.2.1 [7]. In total, approximately 12.0 and 15.0 Gb mRNA FASTQ reads were generated for the May (~38.5 million reads) and July (~48.4 million reads) soil samples, respectively.

To determine the abundance of transcripts from genes annotated in metagenomic assemblies (KEGG and CAZymes), we utilized CoverM in contig mode and normalized read counts to RPKM (bwa-men, --min-read-percent-identity 0.95 --min-read-aligned percent 0.90). The genes predicted from metagenomic assemblies were clustered at 95% sequence identity to produce a non-redundant gene catalog using CD-HIT v4.8.1 [35]. To identify the taxonomic origin of mRNA reads, we assembled the paired-end reads and then performed a BLAST search against the NCBI nr database using DIAMOND v2.0.12 [18] (-sensitive, -e 1e-5). The resulting outputs were then analyzed using MEGAN v6.24.1 [19] with the default parameters.

### *Analysis of soil RNA viruses*

The potential mRNA reads in each library were assembled using the MEGAHIT with default parameters [36] in the metaWRAP v1.3 [37]. We downloaded all viral RNA-dependent RNA polymerase (RdRp) sequences from the NCBI database (March 2023), as the RdRp gene is the most conserved protein in RNA viruses [38]. The assembled contigs (> 300bp) were compared to the RdRp database using DIAMOND [18] (BLASTx, -e 1e-5). The identified RNA viral contigs were then compared to the NCBI nr database using DIAMOND (BLASTx, -max-target-seqs 25, -e 1e-5), and taxonomic assignments were obtained using MEGAN v6.24.1 [19]. We extracted all contigs with hits matching RNA viruses and clustered them at 95% nucleotide identity using CD-HIT (-c 0.95 -n 10 -aS 0.9). The abundances of the RNA viruses were estimated by mapping the mRNA reads to vOTUs using CoverM (bwa-men; identity 0.95, aligned percent 0.90), and read counts were normalized to RPKM.

***Statistical analysis***

Community assembly processes were determined using a quantitative framework based on phylogenetic bin-based null model using “iCAMP” package in R [39]. To apply this model, we first extracted the V3-V4 regions of the 16S rRNA genes from all contigs from 180 metagenomes using the BLASTn (-e 1e-5, -perc_identity 90) and curated SILVA 138 databases. These regions were then filtered (> 400 bp) and clustered into operational taxonomic units (OTUs) based on 97% sequence similarity using CD-HIT. The taxonomic assignment and maximum likelihood-based phylogenetic tree of OTUs were performed on the SILVA website. The script and curated SILVA 138 database (V3-V4 region) for iCAMP analysis were available from GitHub (<https://github.com/beiqicheng/GCEF>). The community was first divided into different groups (‘bins’) based on their phylogenetic relationships with ds = 0.2 and nmin = 24.

Five assembly mechanisms of microbial community including homogeneous selection (HoS), heterogeneous selection (HeS), dispersal limitation (DL), homogenizing dispersal (HD), and drift (DR) were identified in the iCAMP [40]. The beta Net Relatedness Index (βNRI), and taxonomic beta-diversities using modified Raup-Crick metric (RC) were calculated via null model analysis of the phylogenetic diversity to identify the process governing each bin [41]. For each bin, the percentage of pairwise comparisons with βNRI < −1.96 was considered the contribution percentage of HoS, whereas that with βNRI > 1.96 as the contribution percentage of HeS. To assess the effects of treatment on ecological processes, the standardized effect size (Cohen’s *d*) was calculated as the difference of means between treatments divided by the combined standard deviation [40]. The significance of difference in the relative abundance of stochasticity was calculated by bootstrapping for 1,000 repetitions (one-side test).

The clustering analysis was performed using the Mfuzz v2.50.0 [42] package based on the relative abundance of KEGG KOs. This approach is based on soft clustering of time series gene profiles, allowing for KOs to be assigned to multiple clusters with varying levels of membership strength. The average RPKM values of KOs in A and F treatments for each summer were used as input values for clustering. The number of clusters was determined to be 6 based on the Dmin() function and cluster structure in Mfuzz.

**Supplementary Discussion**

We used genome-resolved metagenomics to gain insights into the drought-enriched species and genes as indicated by the community-wide analysis, particularly focusing on N cycling genes (Supplementary Fig. 33). A total of 43 Actinobacteria MAGs were recovered (> 70% completeness and < 10% contamination), whose relative abundance was increased during extreme summers. These MAGs belonged to the orders *Corynebacteriales* (*Mycobacteriales*: GTDB classification), *Propionibacteriales*, *Solirubrobacterales*, *Streptomycetales*, and UBA4738. The *glnA* and *GDH2* genes were present in almost all (> 95%) of the Actinobacteria MAGs. In particular, multiple *glnA* gene copies were encoded in these MAGs, such as the *Mycobacteriales* (3 copies), *Solirubrobacterales* (4 copies), and UBA4738 (6 copies).

To validate our annotation results, we downloaded 625 complete Actinobacteria genomes from the NCBI RefSeq databases (June 2022) and performed functional annotation (see Supplementary Table 9). We found that Actinobacteria with high GC content (usually ≥ 65%) generally contained more copies of the *glnA* gene, e.g., *Mycobacterium* (1~8 copies), *Mycolicibacterium* (1~14 copies), and *Streptomyces* (3~8 copies) species. Moreover, the *GDH2* gene was found to be encoded by high GC content Actinobacteria (average 68%) (Supplementary Table 9). We also identified 8 *GDH2* genes with 1624~1689 amino acids from our Actinobacteria MAGs. A phylogenetic analysis revealed that *GDH2* genes from UBA4738 showed 95.3% amino acid identity to Actinobacteria from agricultural soils in Colorado, USA [43] (see Supplementary Fig. 34). Furthermore, a UBA4738-like MAG has been assembled from sediments in Costa Rica [44], suggesting a potential global distribution.

An early study demonstrated that although *Mycobacterium smegmatis* contains four homologous *glnA* genes, only *gln*A1 is essential for growth [45]. Similar observations have been observed for *Mycobacterium smegmatis* [46] and *Corynebacterium glutamicum* [47]. A recent study revealed that the *gln*A2 gene of *M*. *smegmatis* is essential at high temperatures (42 ℃) [48]. Additionally, the *gln*A3 and *gln*A4 genes in *Streptomyces coelicolor* have been shown to be involved in the degradation of organic compounds [49, 50]. The GDH pathway has a low affinity to ammonium, which often acts in N assimilation when ammonia is in excess (> 1 mM) [51]. However, it was found that the activity of *GDH2* genes in *M*. *smegmatis* was significantly higher during N starvation [52]. Although the exact roles of *glnA* homologs and *GDH2* remain unclear, we speculate that these genes may provide Actinobacteria with a competitive advantage for N assimilation under drought stress.


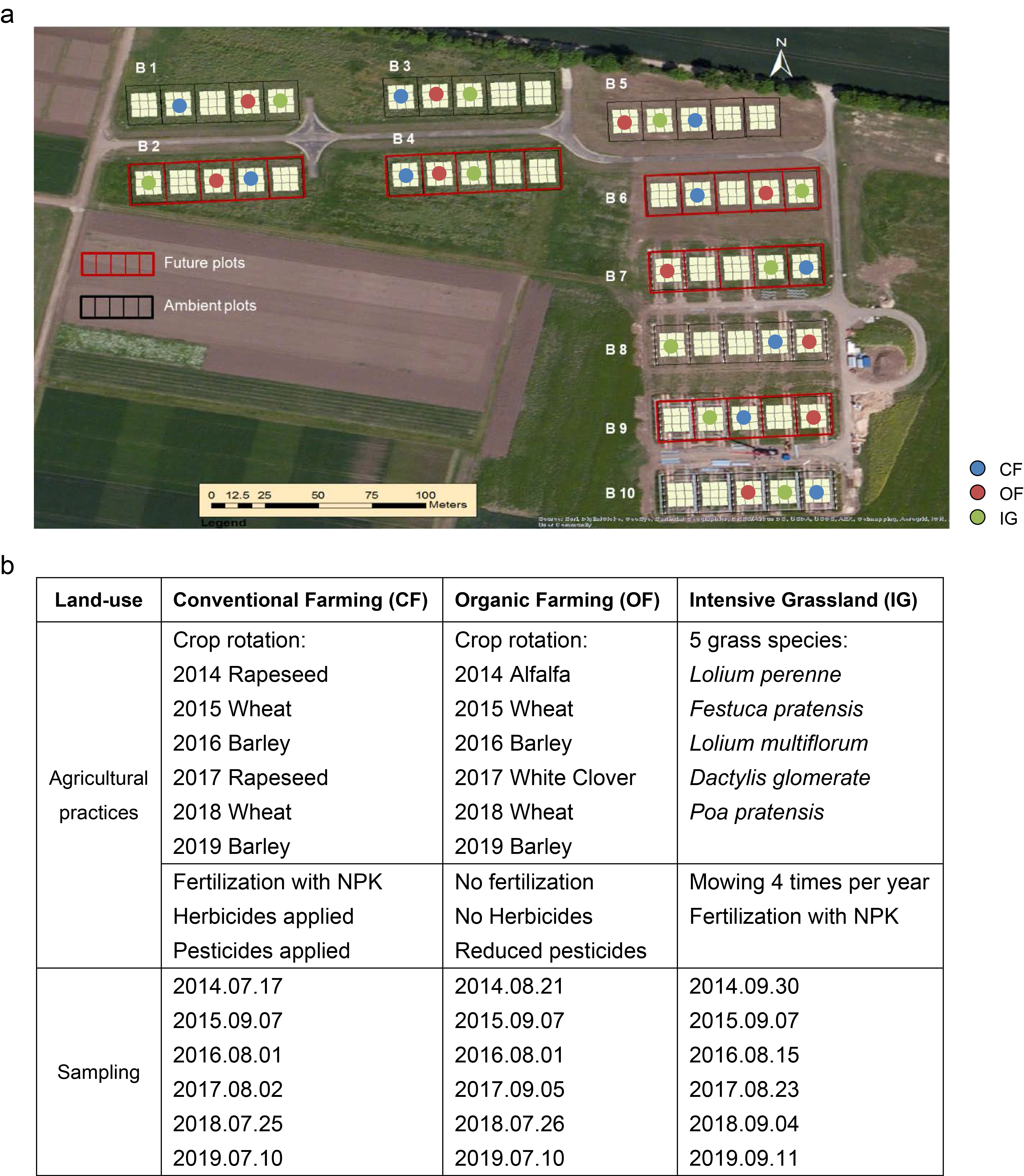


**Fig. S1:** Layout of the GCEF research station (a), agricultural practices, and sampling dates for different land-use types (b). For more details please see Schädler et al. [5].


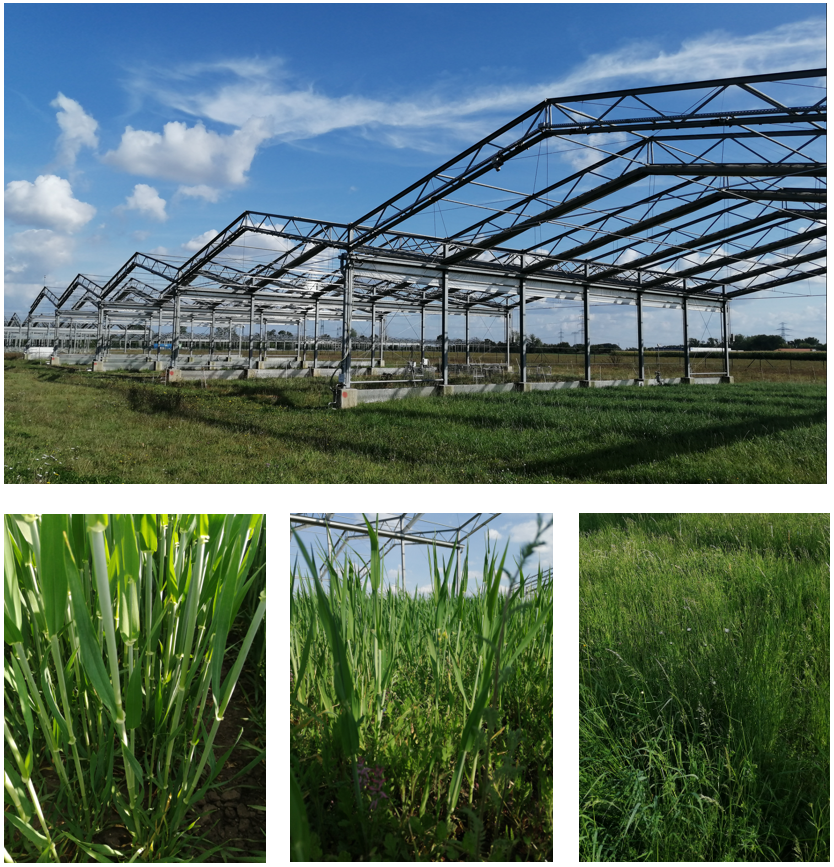


­­ CF: conventional farming OF: organic farming IG: intensive grassland

**Fig. S2:** Photos of the GCEF mainplot consisting of different land-use types. Photos of three land-use types were taken in Spring 2022.


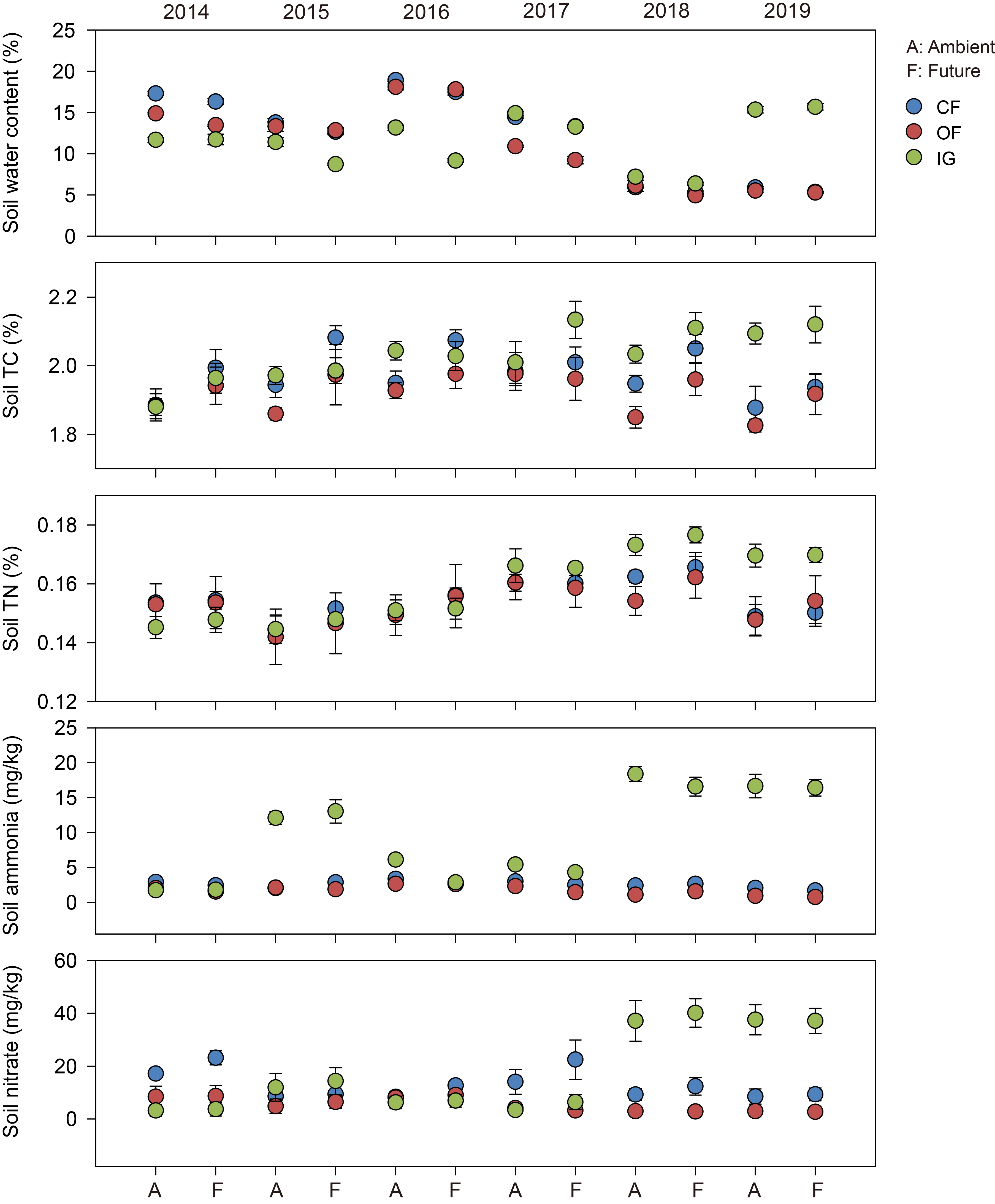


**Fig. S3:** Physicochemical properties (moisture, total carbon, total nitrogen, ammonium, and nitrate concentrations) of soil samples during the summers of 2014–2019. CF: conventional farming; OF: organic farming; IG: intensive grassland.


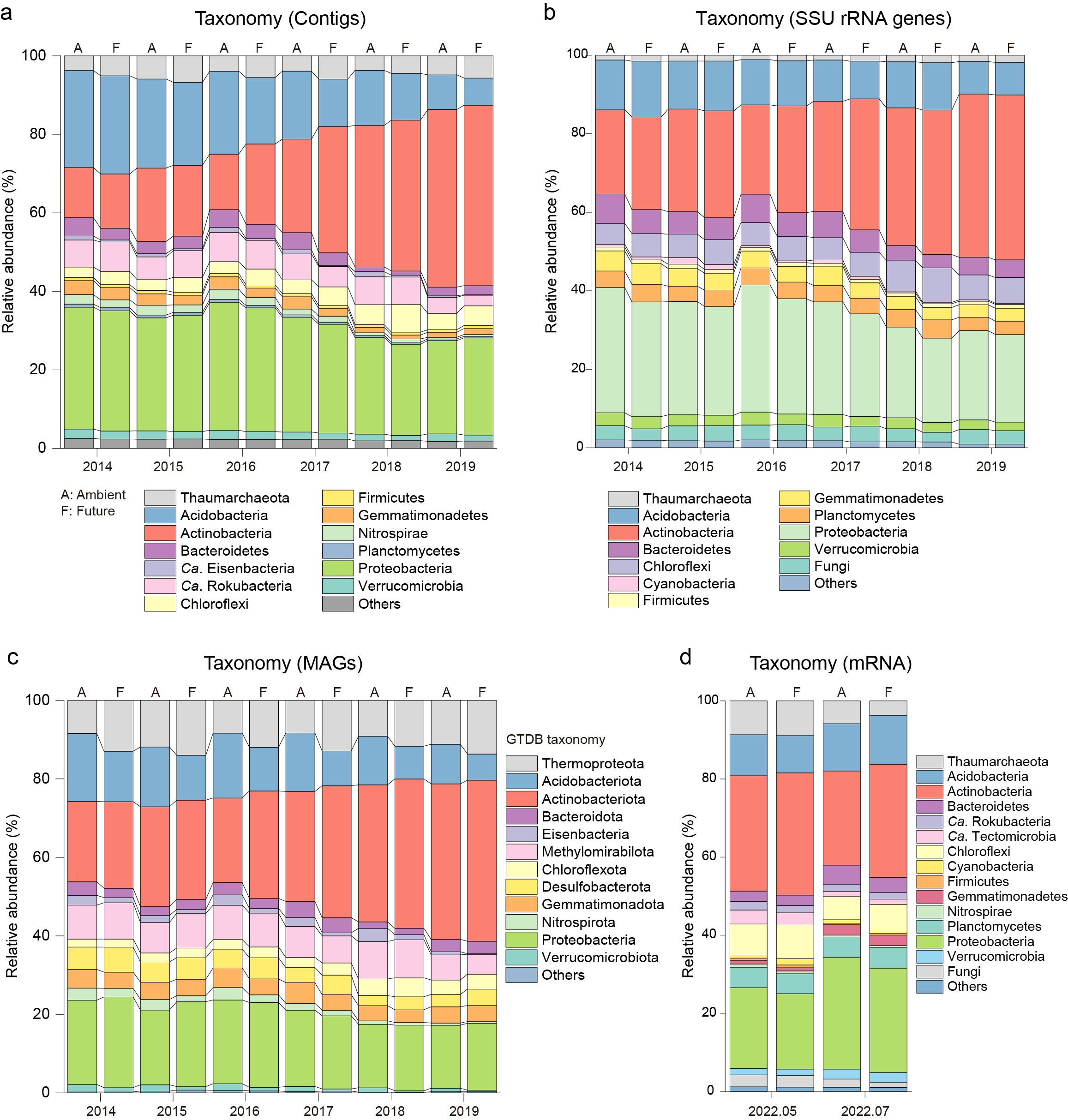


**Fig. S4:** Community composition based on soil metagenomics and metatranscriptomics. Stacked bar charts showing phylum-level community composition based on contigs (a), SSU rRNA genes (b) and recovered metagenome-assembled genomes (MAGs) (c) from metagenomics, and mRNA (d) from metatranscriptomics. The metagenomic reads were mapped back to classified contigs and MAGs and normalized to RPKM. Taxonomic groups with a relative abundance of under 0.5% were combined into the “Others”.


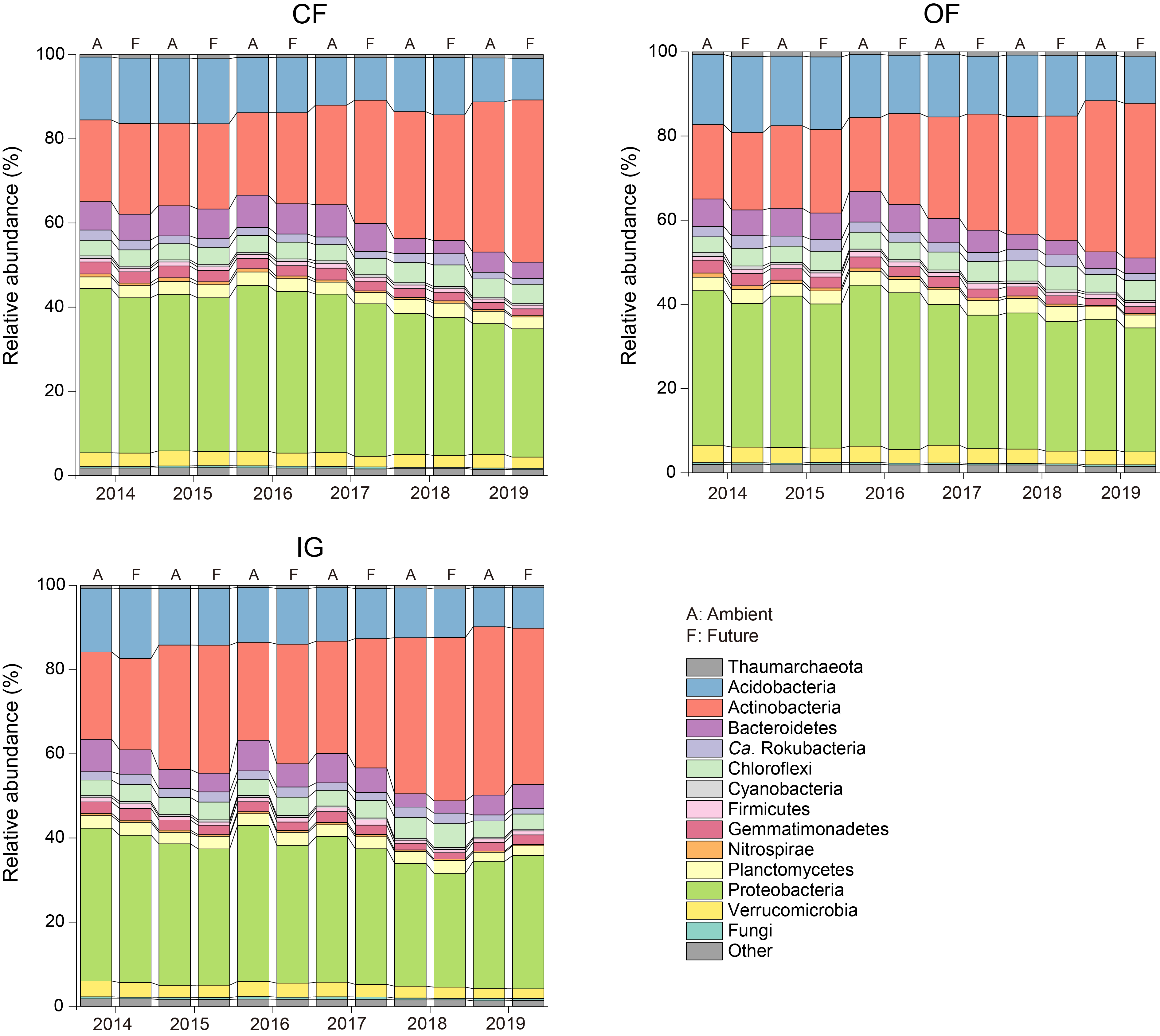


**Fig. S5:** Stacked bar charts showing the relative abundances of dominant phyla under different land-use types during the summers of 2014–2019. The community composition was based on the metagenomic FASTQ reads using Kaiju with NCBI nr database. Taxonomic groups with a relative abundance of under 0.5% were combined into the “Other” group. CF: conventional farming; OF: organic farming; IG: intensive grassland.


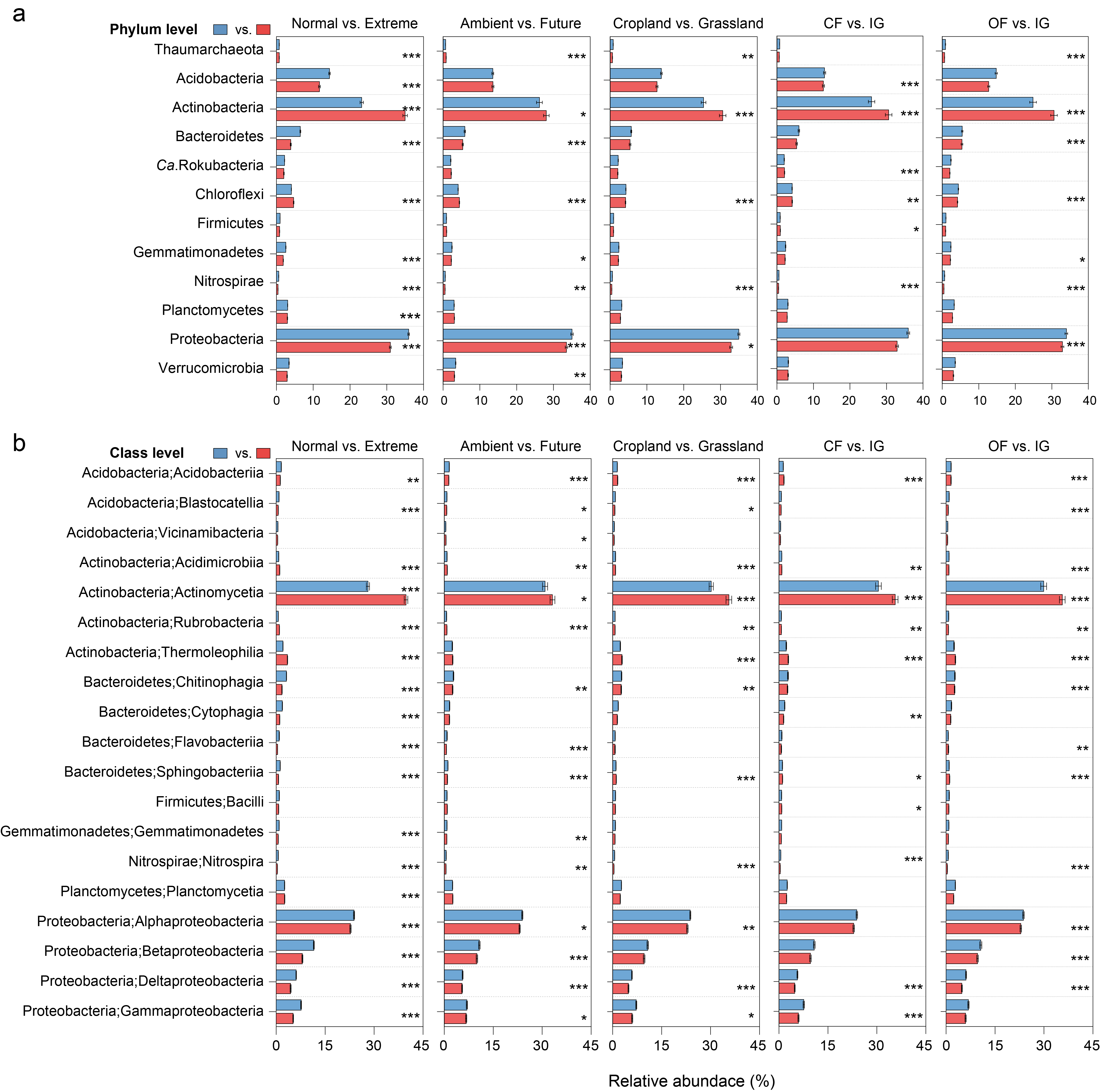


**Fig. S6:** Impact of climate and land-use changes on soil microbial communities at phylum (a) and class (b) level during the summers of 2014–2019. The taxonomic profiles were generated from the metagenomic FASTQ reads using Kaiju with NCBI nr database. The error bars show the standard error in each condition. Significantly altered phyla are marked with asterisks based on the DESeq2 Benjamini and Hochberg (BH)-adjusted *p* value, significance levels: **p* < 0.05; ***p* < 0.01; ****p* < 0.001. CF: conventional farming; OF: organic farming; IG: intensive grassland.


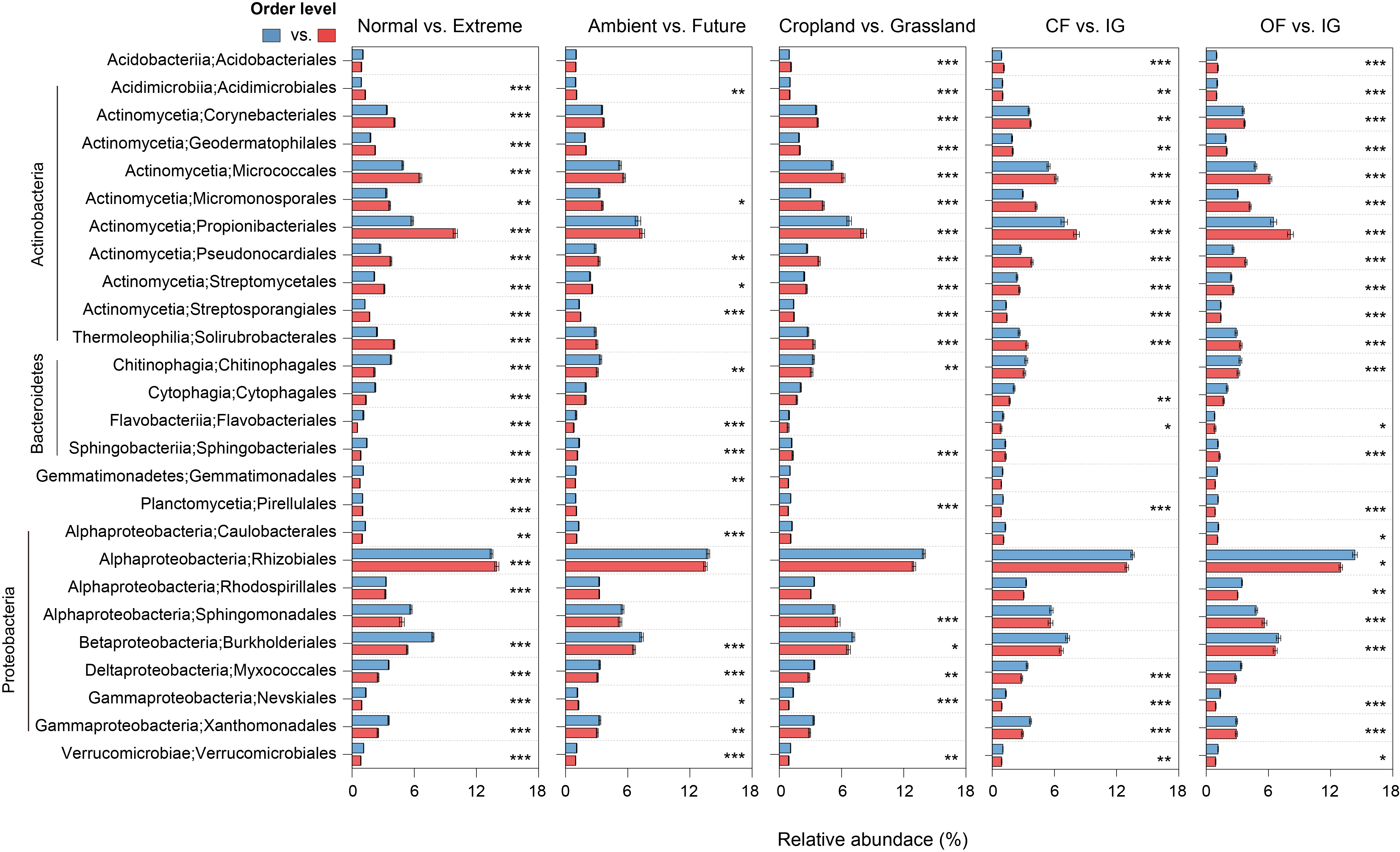


**Fig. S7:** Impact of climate and land-use changes on soil microbial communities at order level during the summers of 2014–2019. The taxonomic profiles were generated from the metagenomic FASTQ reads (with relative abundance > 0.5%). The error bars show the standard error in each condition. Significantly altered orders are marked with asterisks based on DESeq2 BH-adjusted *p* value, significance levels: **p* < 0.05; ***p* < 0.01; ****p* < 0.001. CF: conventional farming; OF: organic farming; IG: intensive grassland.


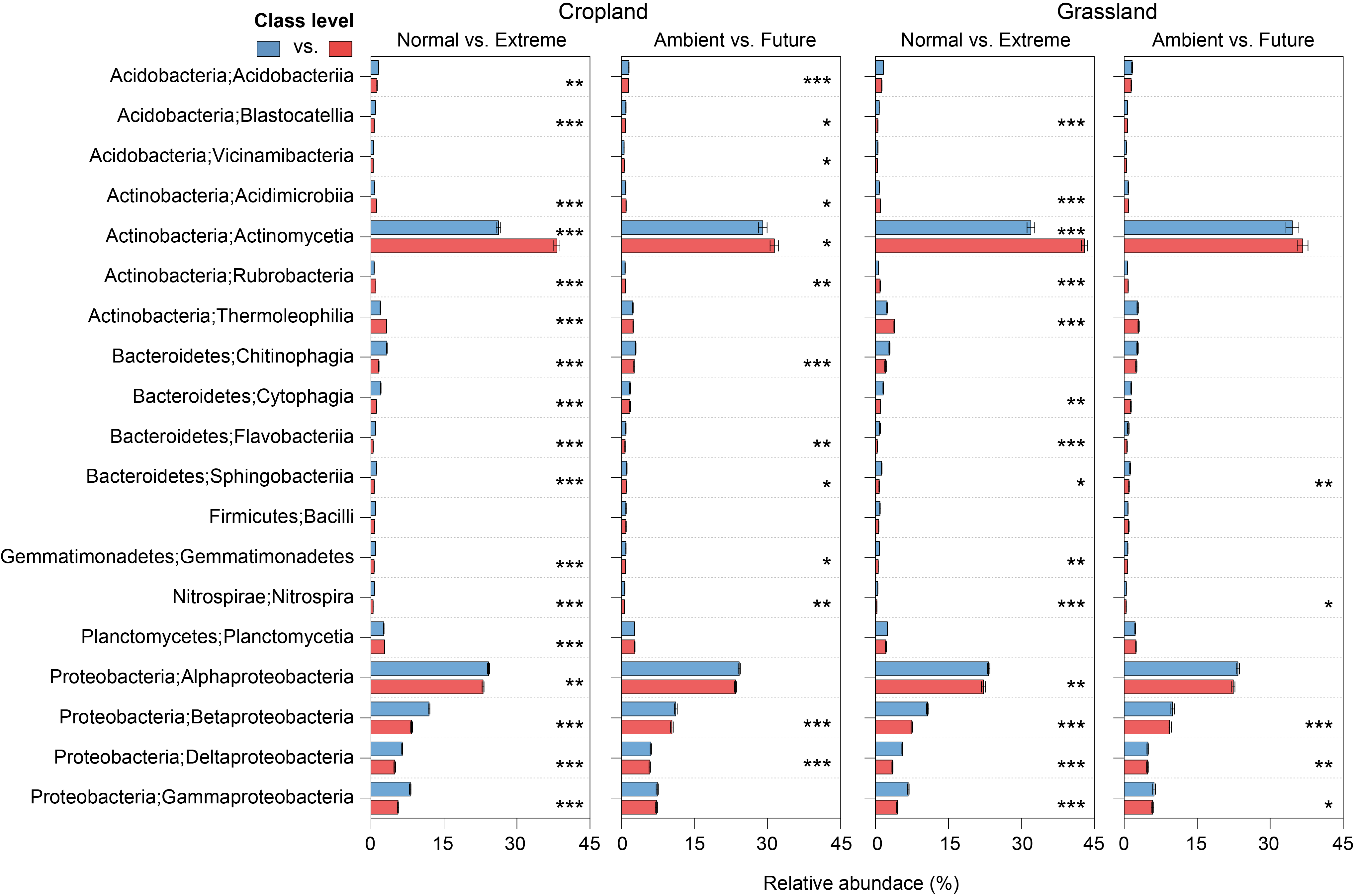


**Fig. S8:** Impact of climate change on soil microbial community structure at class level in cropland and grassland. The taxonomic profiles were generated from the metagenomic FASTQ reads (with relative abundance > 0.5%). The error bars show the standard error in each condition. Significantly altered classes are marked with asterisks based on the DESeq2 BH-adjusted *p* value, significance levels: **p* < 0.05; ***p* < 0.01; ****p* < 0.001.


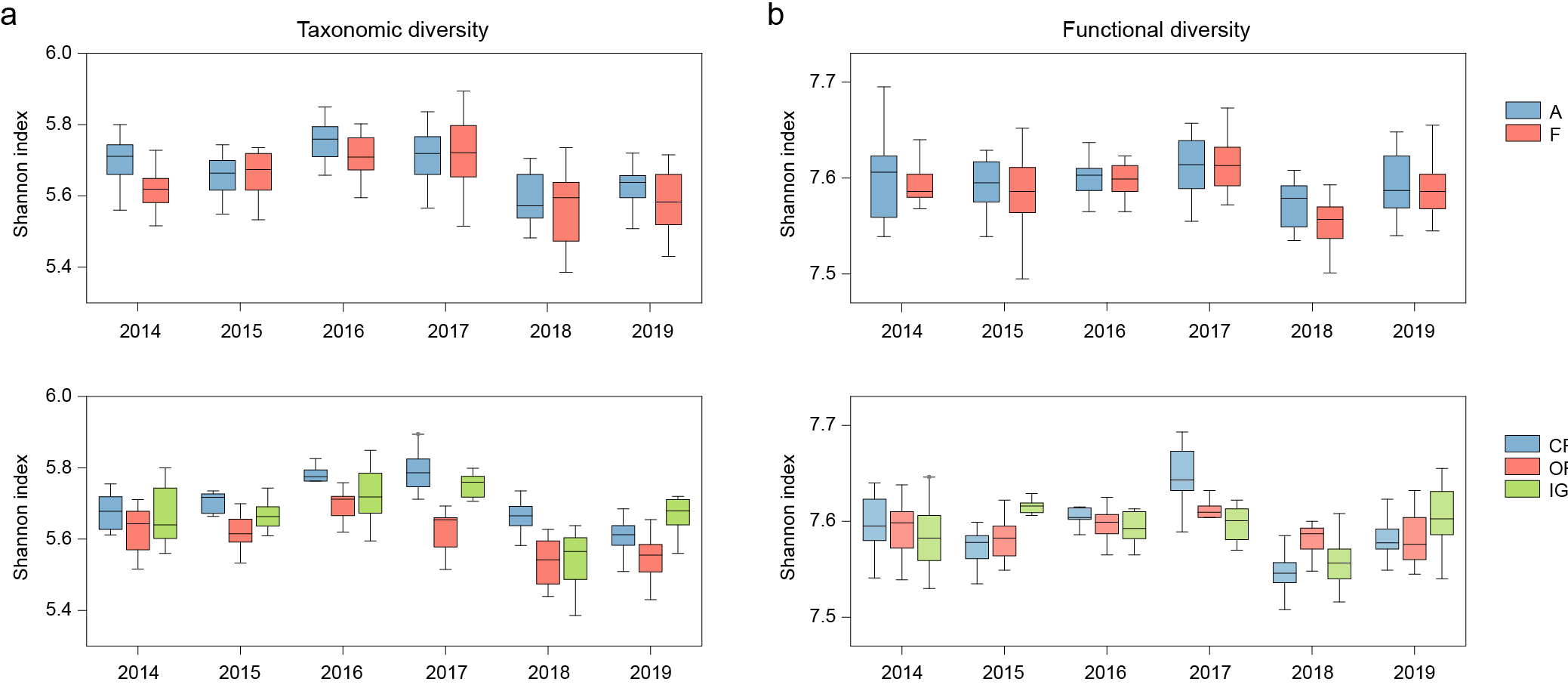


**Fig. S9:** Boxplots of the Shannon index of soil microbial communities at genus (a) and KEGG KO (b) level during the summers of 2014–2019. The Shannon index was calculated using SingleM based on the *rplB* gene. Boxplots show median, upper and lower quartile, and minimum and maximum values. Effects of extreme summers, climate change, and land-use on the Shannon index were calculated based on linear mixed-effects models (see Supplementary Table S3 for statistical results). CF: conventional farming; OF: organic farming; IG: intensive grassland.


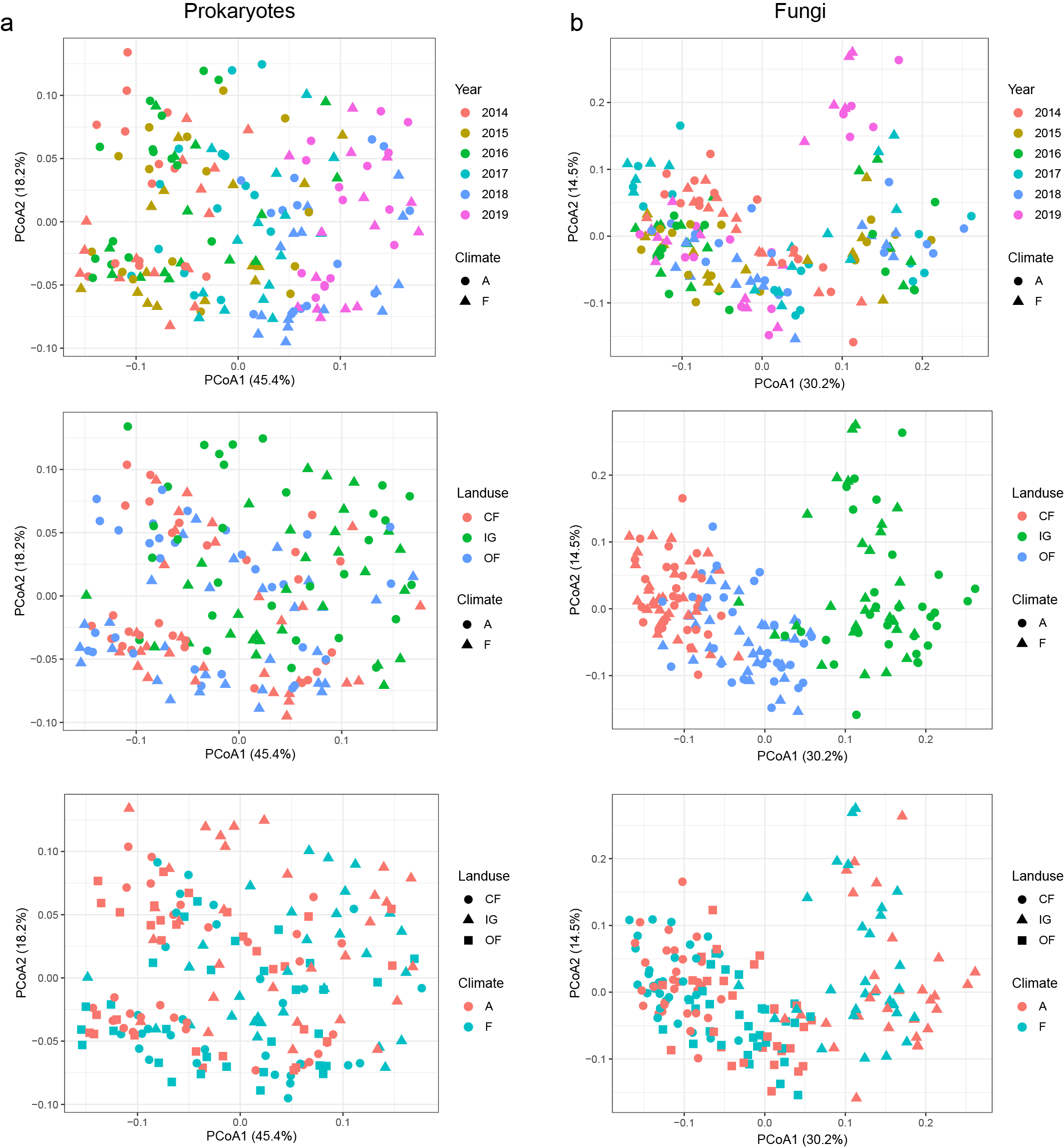


**Fig. S10:** PCoA plots based on the Bray–Curtis distances of soil prokaryotic (a) and fungal (b) communities at genus level. The taxonomic profiles were generated from the metagenomic FASTQ reads. CF: conventional farming; OF: organic farming; IG: intensive grassland.


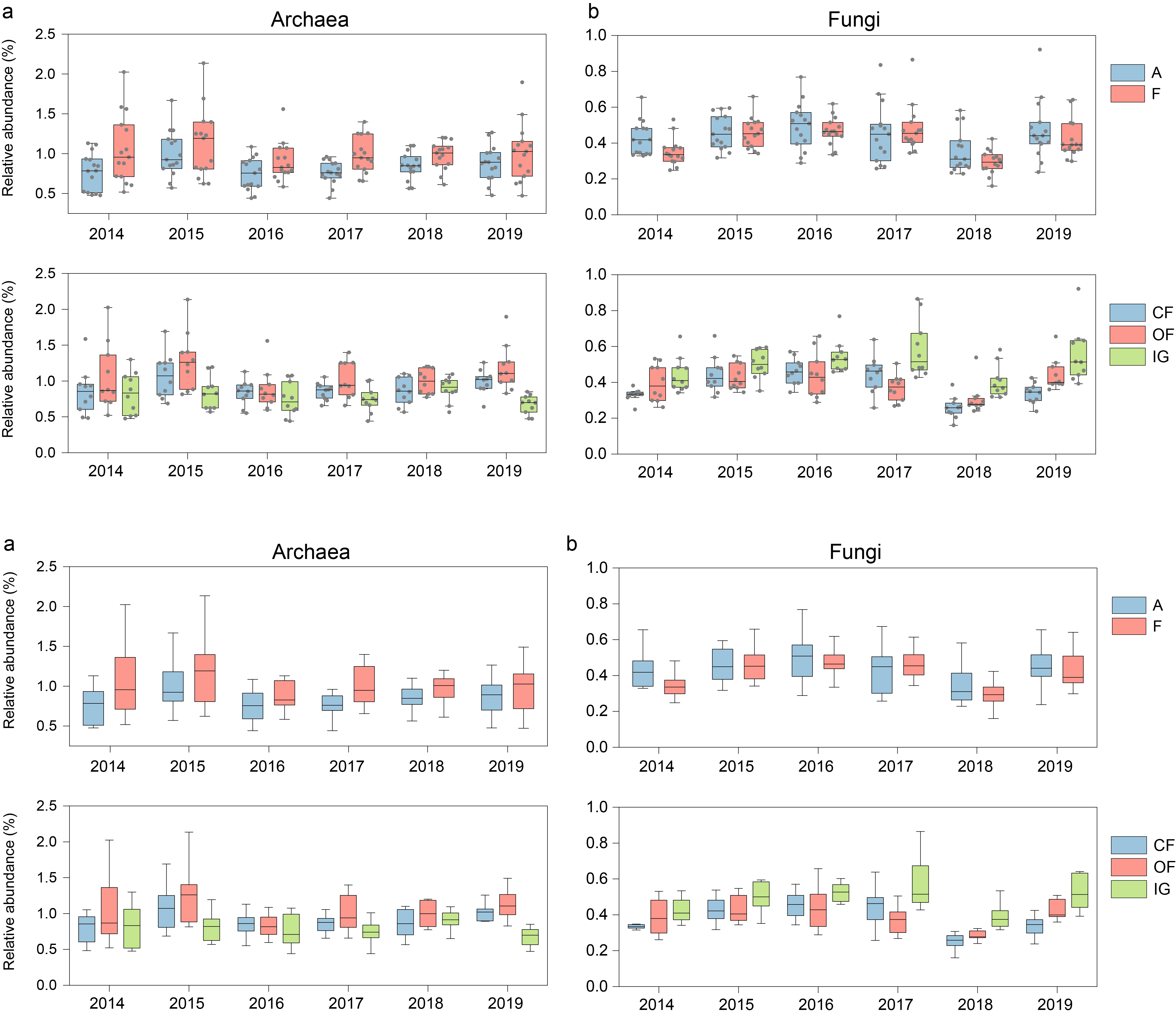


**Fig. S11:** The relative abundance of archaea (a) and fungi (b) in metagenomics during the summers of 2014–2019. Boxplots show median, upper and lower quartile, and minimum and maximum values. The community composition was based on the metagenomic FASTQ reads using Kaiju. Effects of extreme summers, climate change, and land-use on the relative abundance were calculated based on linear mixed-effects models.


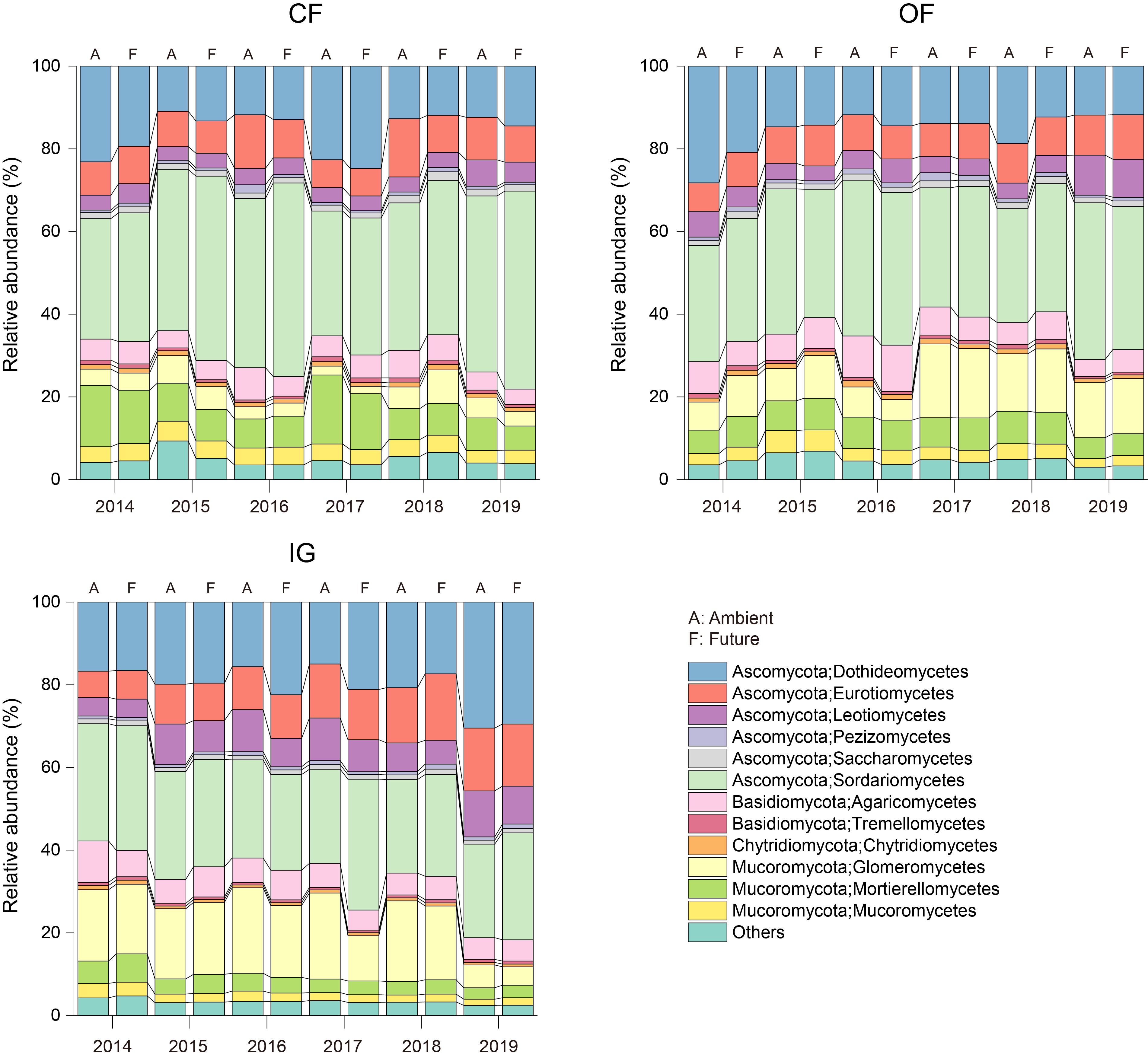


**Fig. S12:** Stacked bar charts showing the relative abundances of fungal classes under different land-use types during the summers of 2014–2019. The community composition was based on the metagenomic FASTQ reads using Kaiju with NCBI nr database. Taxonomic groups with a relative abundance of under 0.5% were combined into the “Other” group.


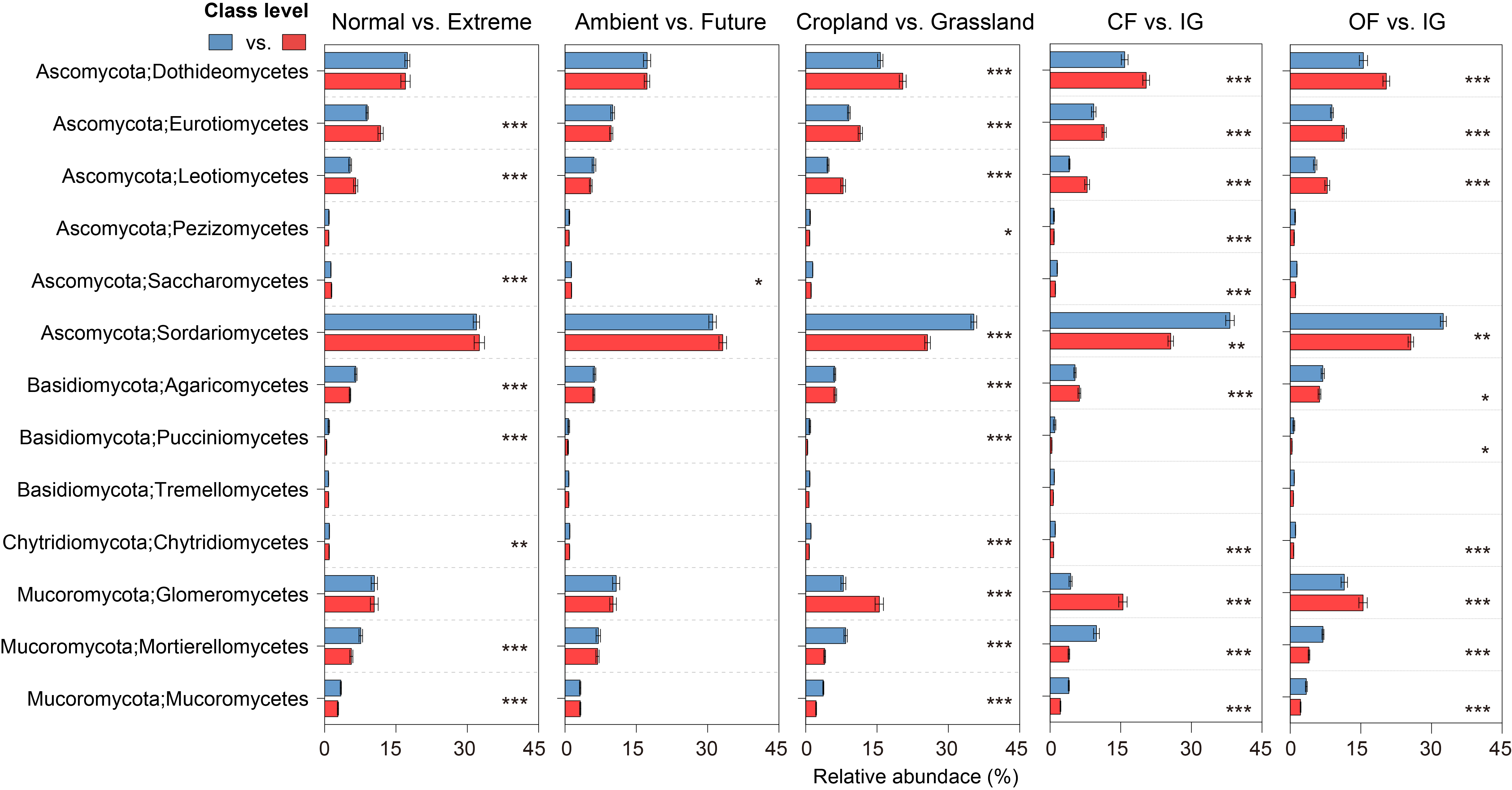


**Fig. S13:** Impact of climate and land-use changes on soil fungal communities at class level during the summers of 2014–2019. The taxonomic profiles were generated from the metagenomic FASTQ reads (with relative abundance > 0.5%). The error bars show the standard error in each condition. Significantly altered fungal classes are marked with asterisks based on DESeq2 BH-adjusted *p* value, significance levels: **p* < 0.05; ***p* < 0.01; ****p* < 0.001. CF: conventional farming; OF: organic farming; IG: intensive grassland.


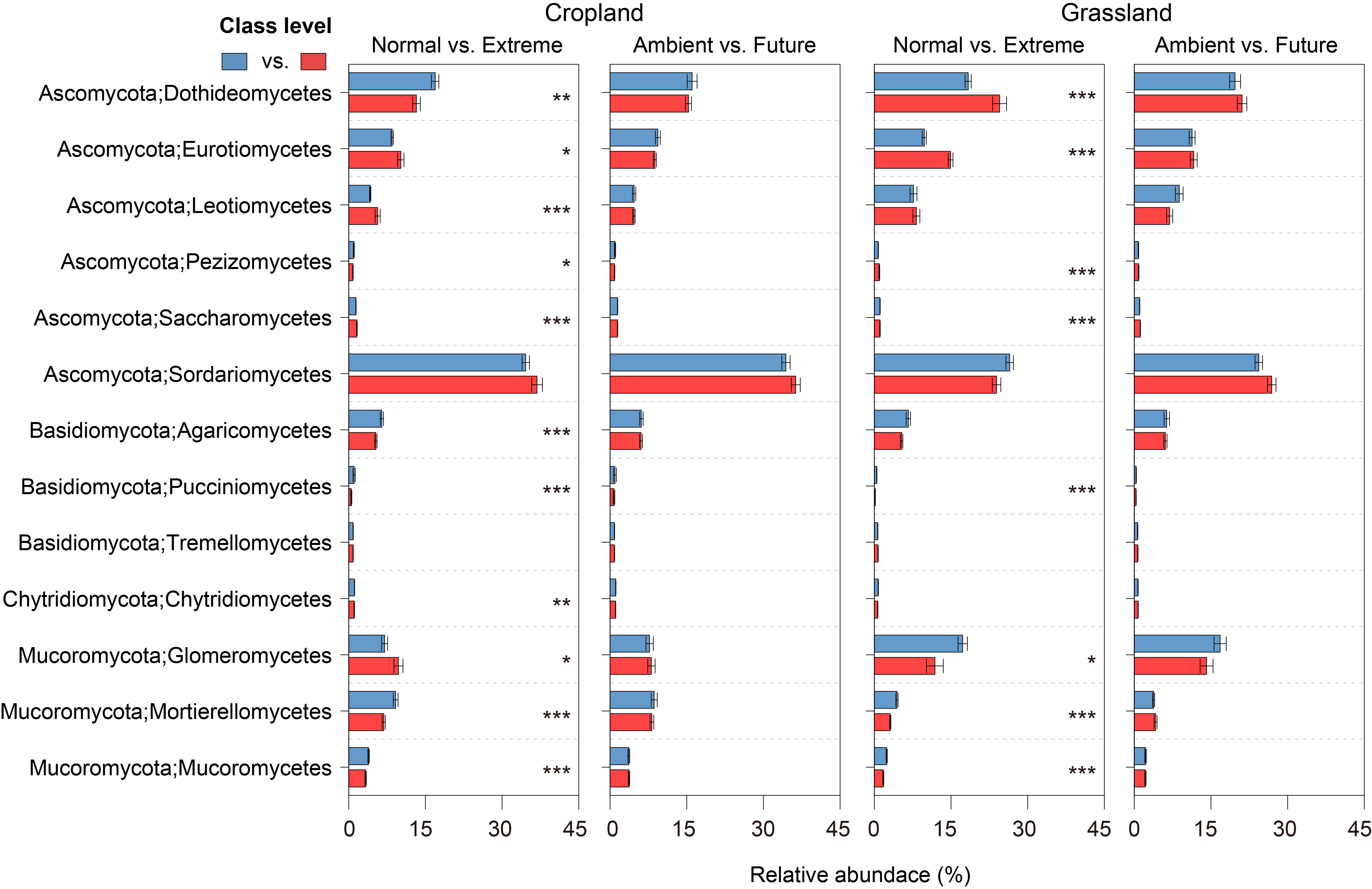


**Fig. S14:** Impact of climate change on soil fungal communities at class level in cropland and grassland. The taxonomic profiles were generated from the metagenomic FASTQ reads using Kaiju (with relative abundance > 0.5%). The error bars show the standard error in each condition. Significantly altered classes are marked with asterisks based on DESeq2 BH-adjusted *p* value, significance levels: **p* < 0.05; ***p* < 0.01; ****p* < 0.001.


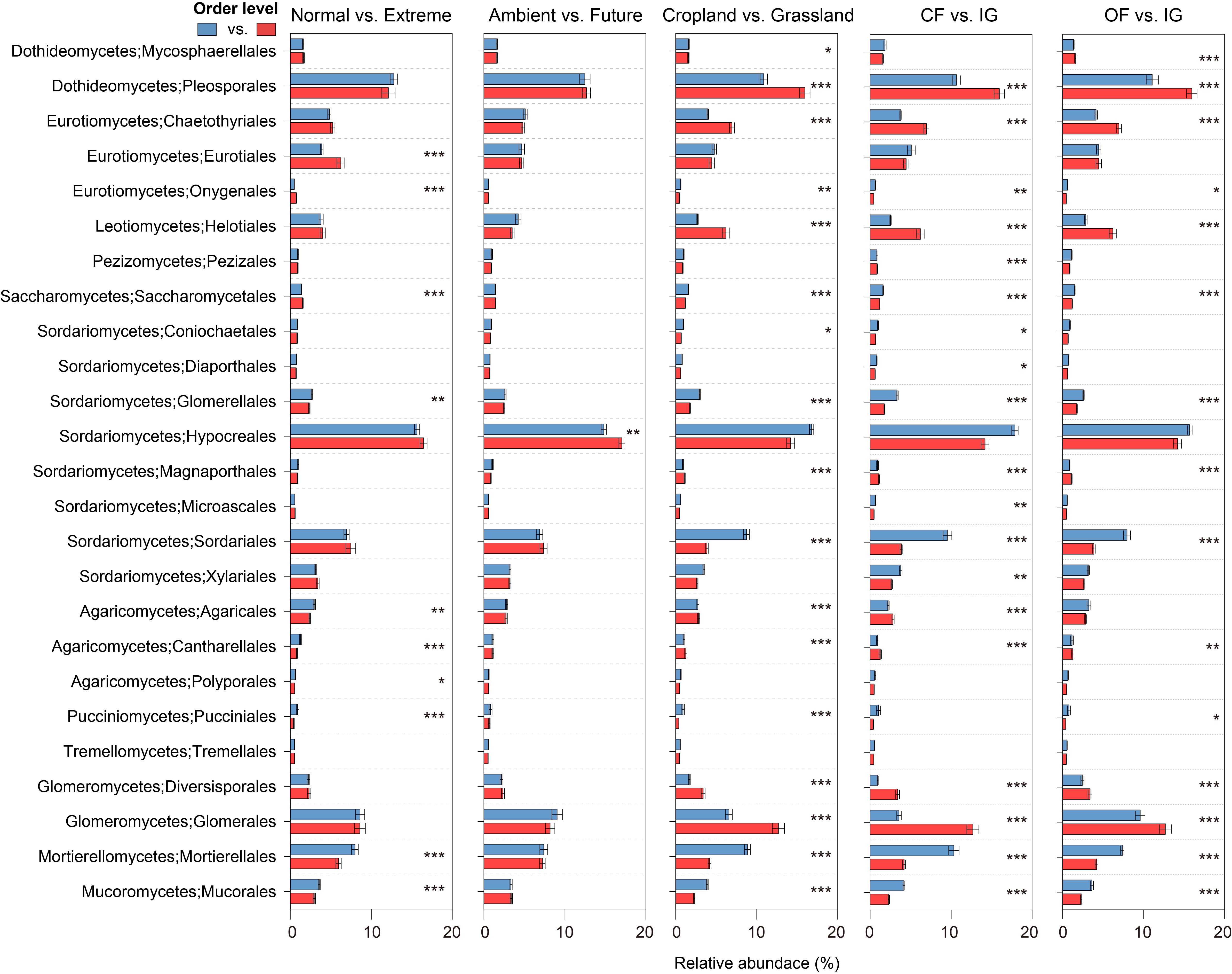


**Fig. S15:** Impact of climate and land-use changes on soil fungal communities at order level during the summers of 2014–2019. The taxonomic profiles were generated from the metagenomic FASTQ reads using Kaiju (with relative abundance > 0.5%). The error bars show the standard error in each condition. Significantly altered fungal orders are marked with asterisks based on DESeq2 BH-adjusted *p* value, significance levels: **p* < 0.05; ***p* < 0.01; ****p* < 0.001. CF: conventional farming; OF: organic farming; IG: intensive grassland.


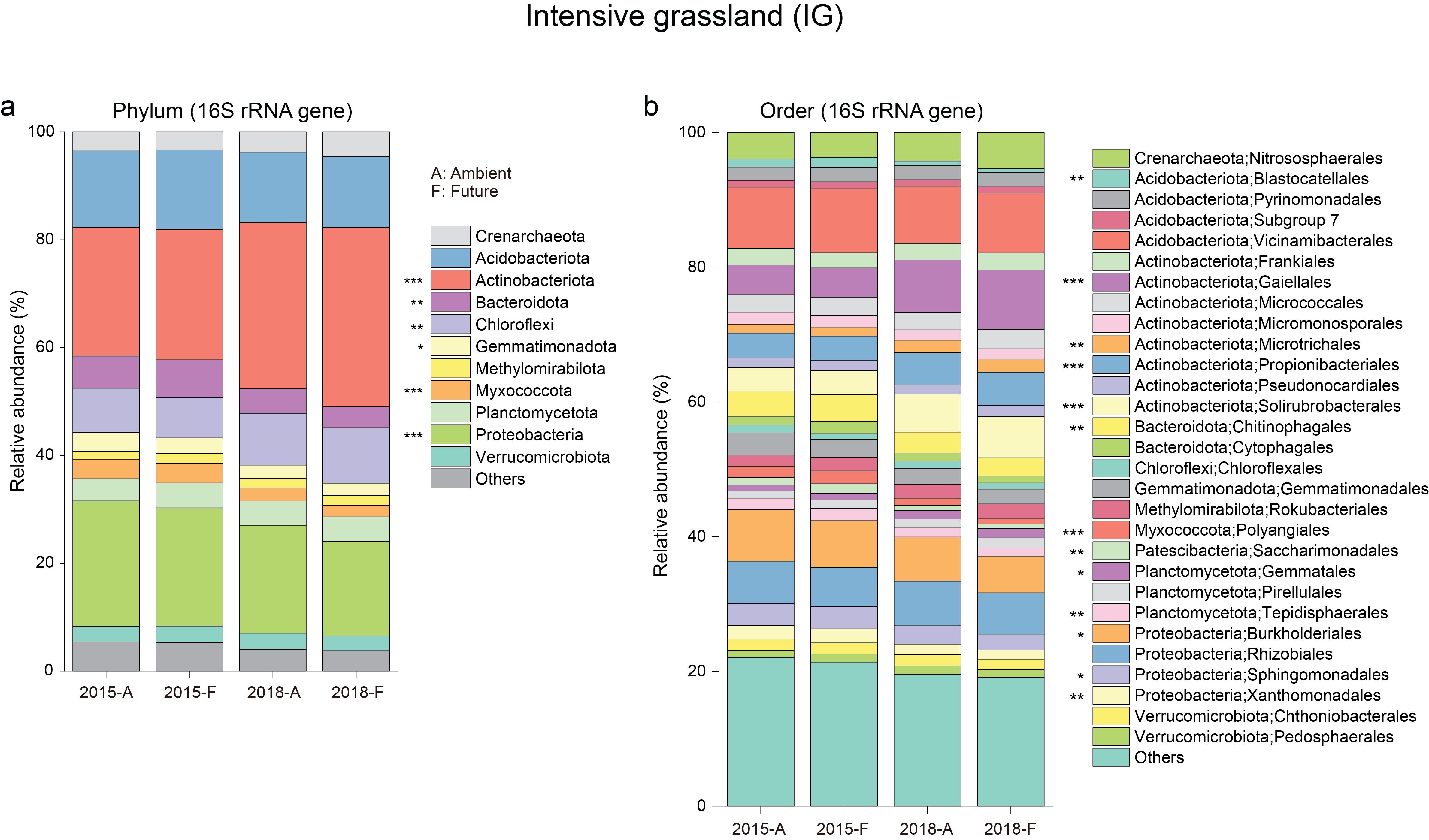


**Fig. S16:** Amplicon analysis of climate change effects on soil bacterial communities in 2015 and 2018 summers. The community composition was based on the 16S rRNA genes using SILVA 138 database. Taxonomic groups with a relative abundance < 1 % were combined into the “Other” group. Bacterial phyla and orders that significantly affected by extreme summers are marked with asterisks based on the *t*-test and adjusted by Benjamini–Hochberg method (adjusted *p* value, **p* < 0.05; ***p* < 0.01; ****p* < 0.001).


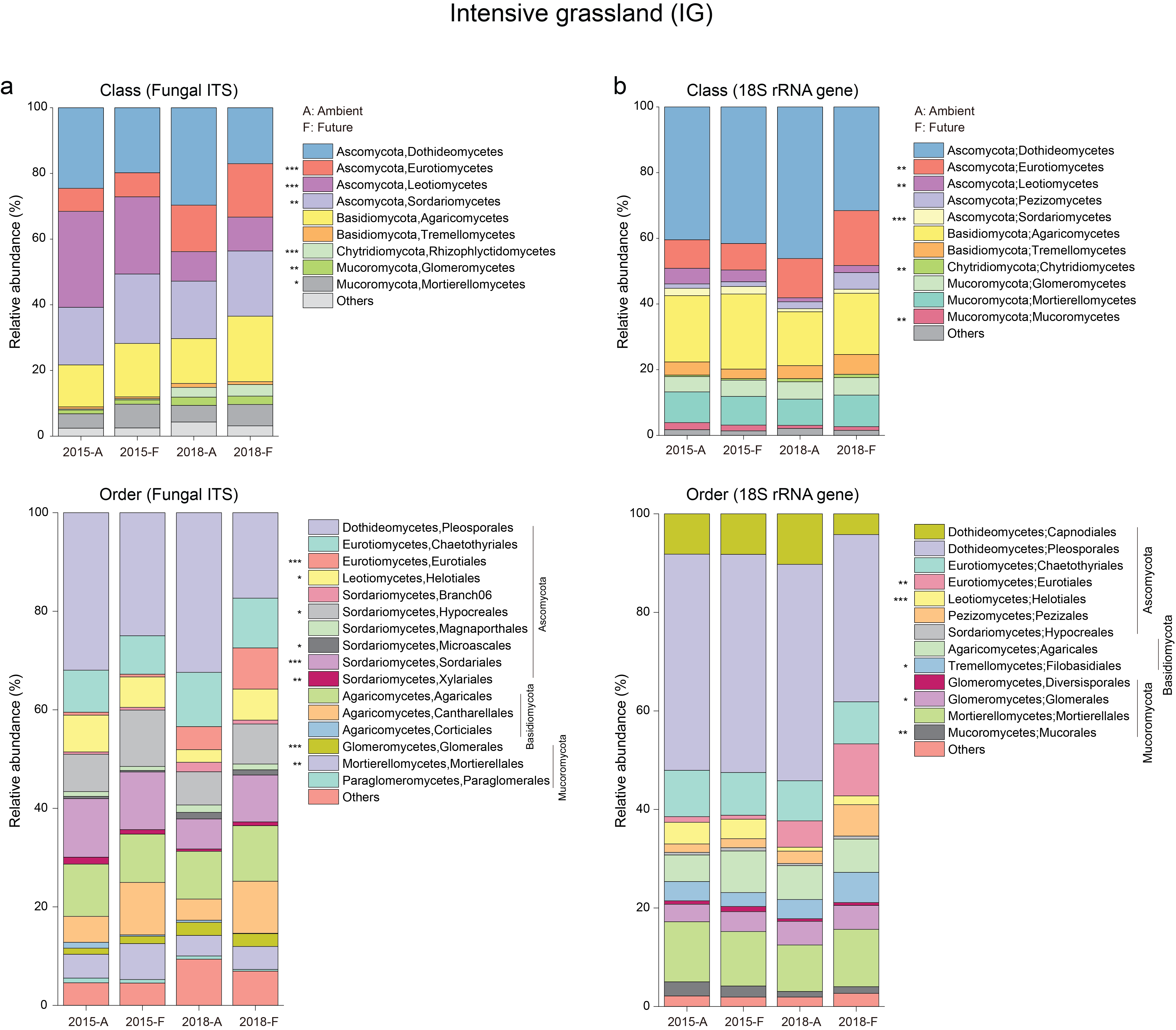


**Fig. S17:** Amplicon analysis of climate change effects on soil fungal communities in 2015 and 2018 summers. The community composition was based on the fungal ITS (a) and 18S rRNA genes (b). Taxonomic groups with a relative abundance of under 0.5% were combined into the “Other” group. Fungal classes and orders that significantly affected by extreme summers are marked with asterisks based on the *t*-test and adjusted by Benjamini–Hochberg method (adjusted *p* value, **p* < 0.05; ***p* < 0.01; ****p* < 0.001).


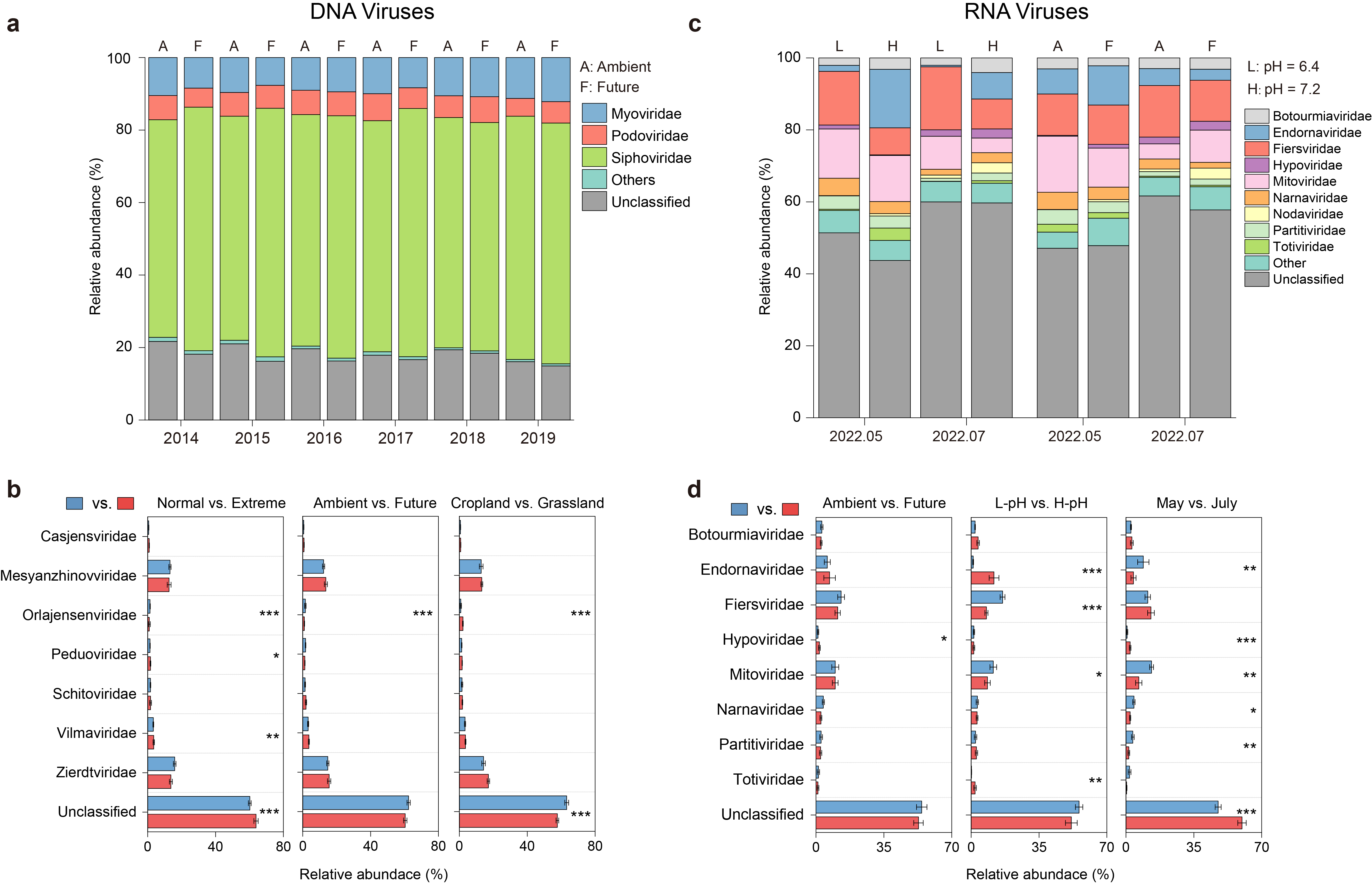


**Fig. S18:** Taxonomy of soil DNA and RNA viruses. Stacked bar chart showing family-level community composition of DNA viruses based on the BLAST method (a). Effects of climate and land-use changes on DNA viral communities at family level during the summers of 2014–2019 (b). Stacked bar chart showing family-level community composition of RNA viruses (c). Effects of climate and soil pH change on RNA viral communities at family level in 2022 (d). The error bars show the standard error in each treatment. Significantly altered families are marked with asterisks based on DESeq2 BH-adjusted *p* value, significance levels: **p* < 0.05; ***p* < 0.01; ****p* < 0.001.


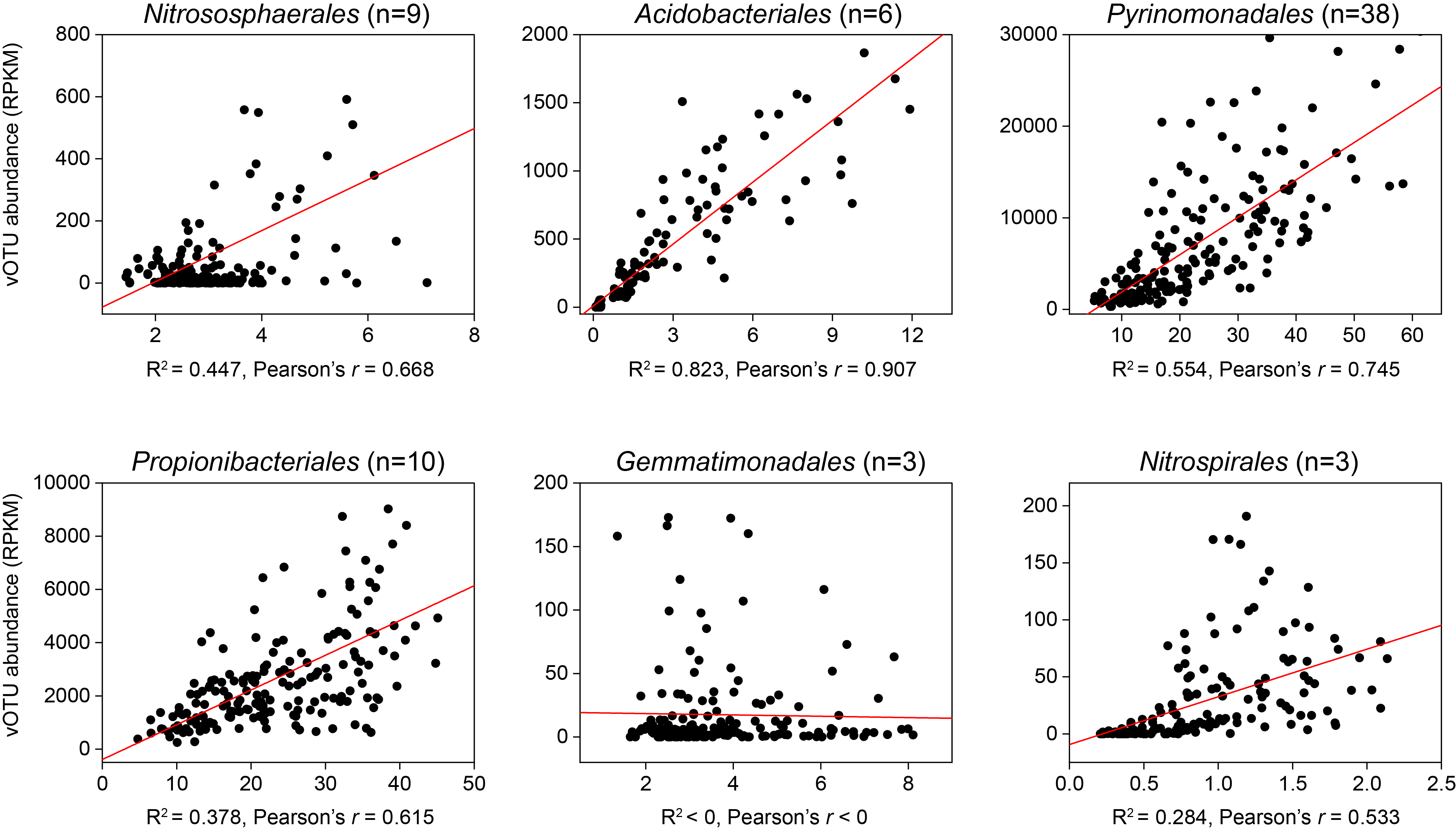


**Fig. S19:** Viral-host abundance patterns based on the lineage-specific vOTU–MAG. Host abundance and the abundance of DNA viruses for that host (both calculated as RPKM values) are shown at order level. The number (n) of linked vOTU-MAG is indicated at the top of each plot. Only vOTUs present in more than 20% of all samples were selected. Based on linear regression analysis, the best-fit lines (red), R^2^, and Pearson’s *r* values for each order are presented.


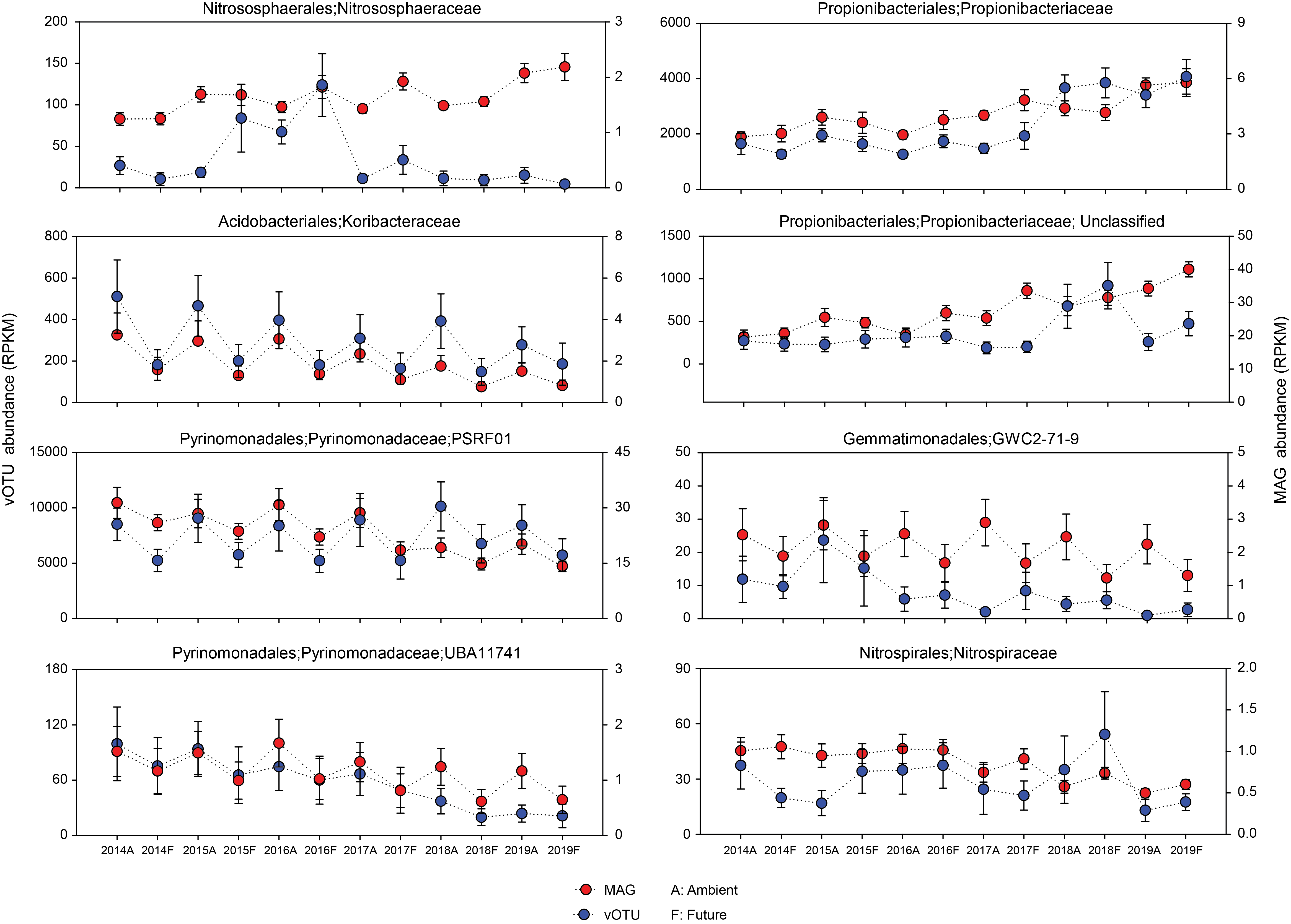


**Fig. S20:** vOTU–MAG abundance patterns across 6 years. Host abundance and the abundance of DNA viruses (both calculated as RPKM values) are shown based on the taxonomic level of MAGs according to the GTDB classification.


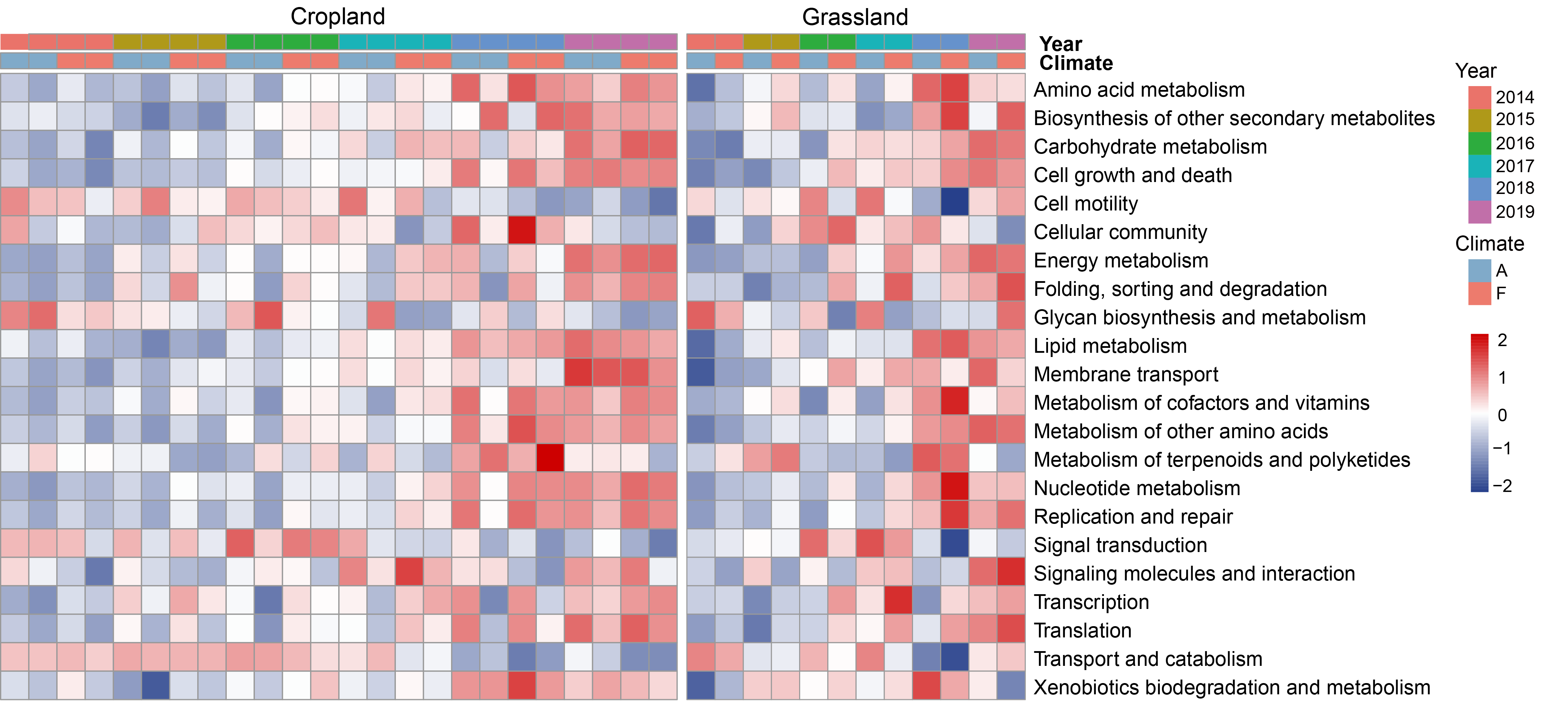


**Fig. S21:** Heatmap of microbial functional profiles based on the main KEGG pathways in cropland and grassland. The RPKM value of KEGG pathway (Level 2) was log_10_ transformed before row scaling. The row scaling was performed separately for cropland and grassland.


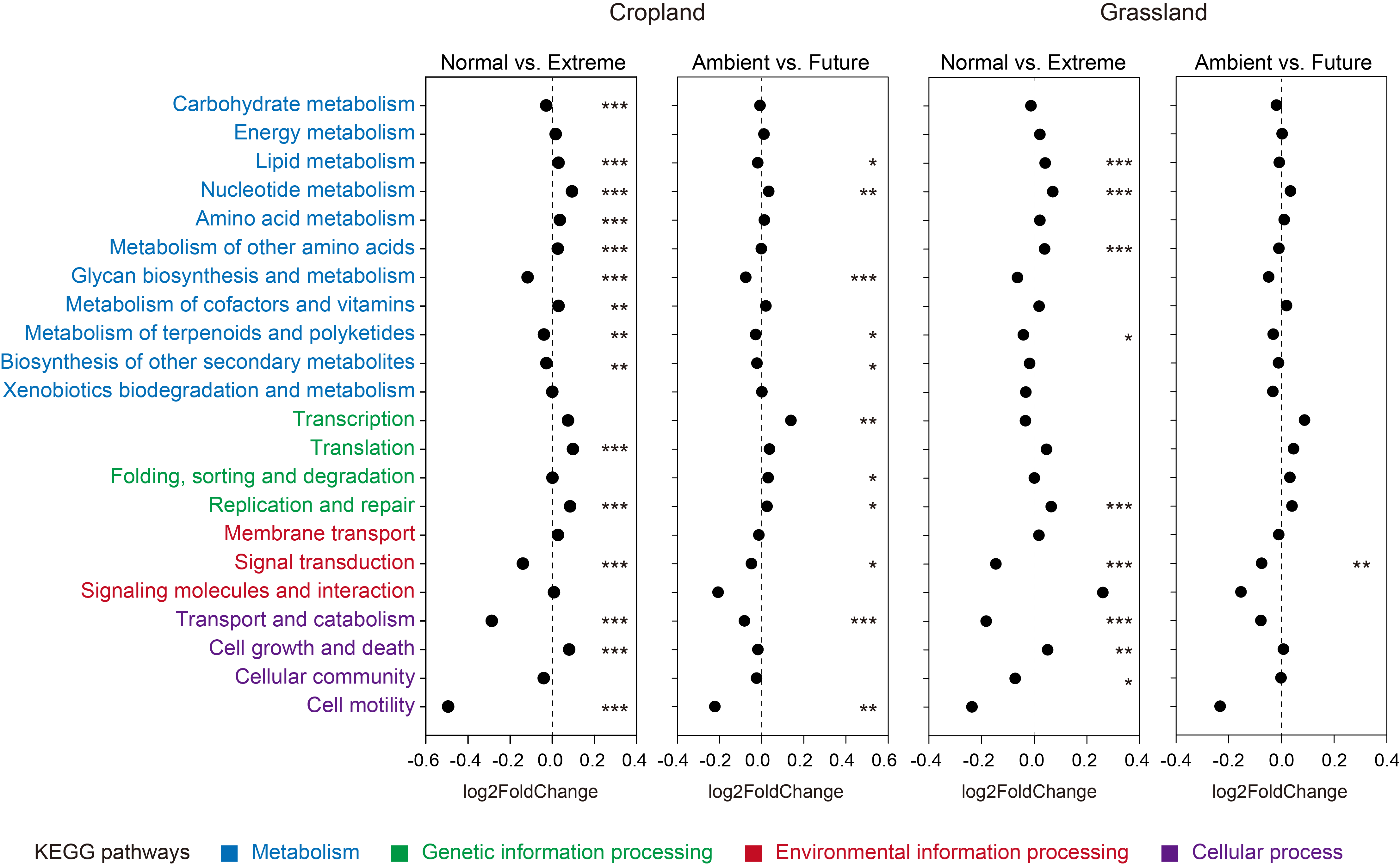


**Fig. S22:** Impact of climate change on the main KEGG pathways of soil microbial communities in cropland and grassland. Significantly altered pathways are marked with asterisks based on DESeq2 BH-adjusted *p* value, significance levels: **p* < 0.05; ***p* < 0.01; ****p* < 0.001.


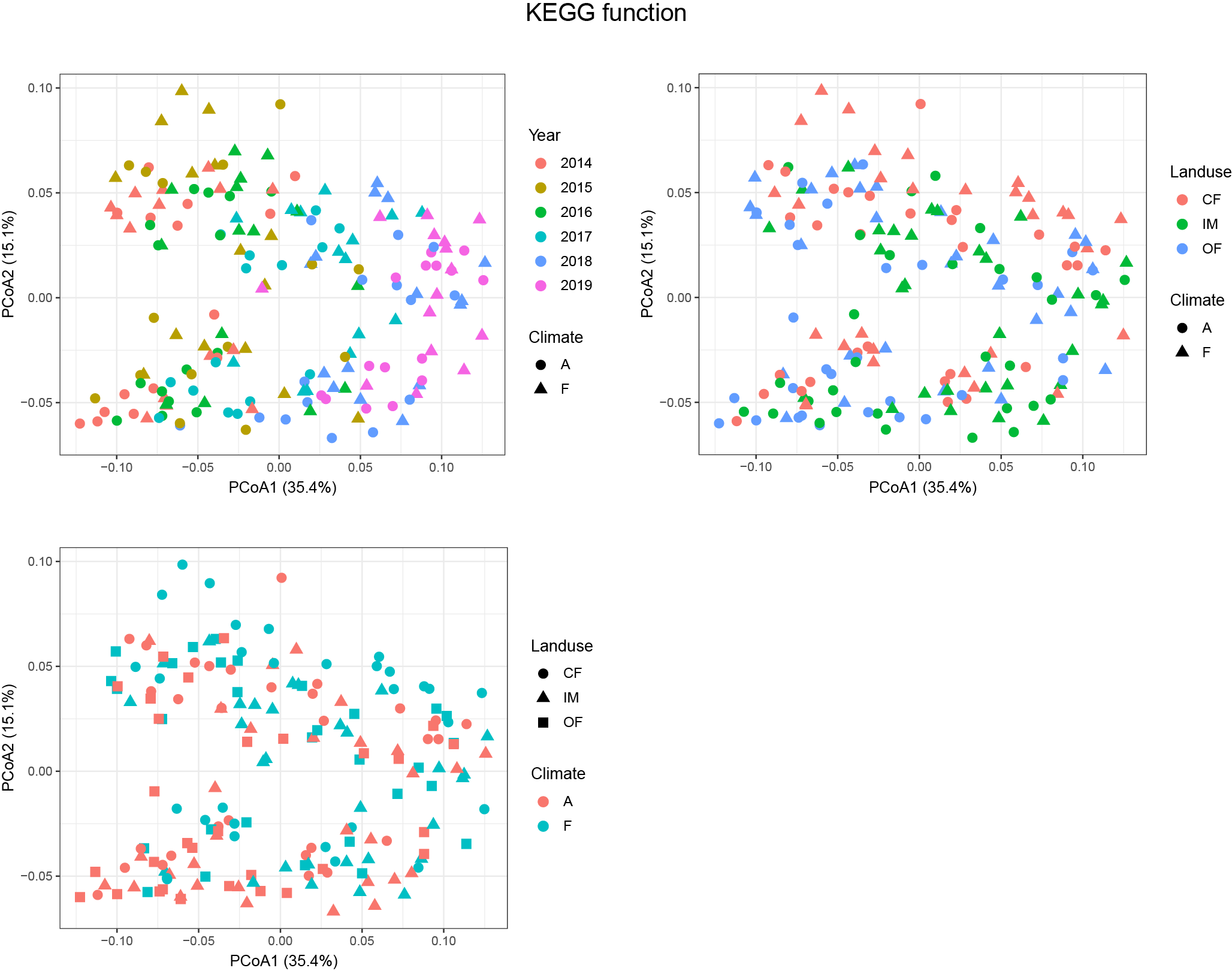


**Fig. S23:** PCoA plots based on the Bray–Curtis distances of soil microbial functions (KO level). CF: conventional farming; OF: organic farming; IG: intensive grassland.


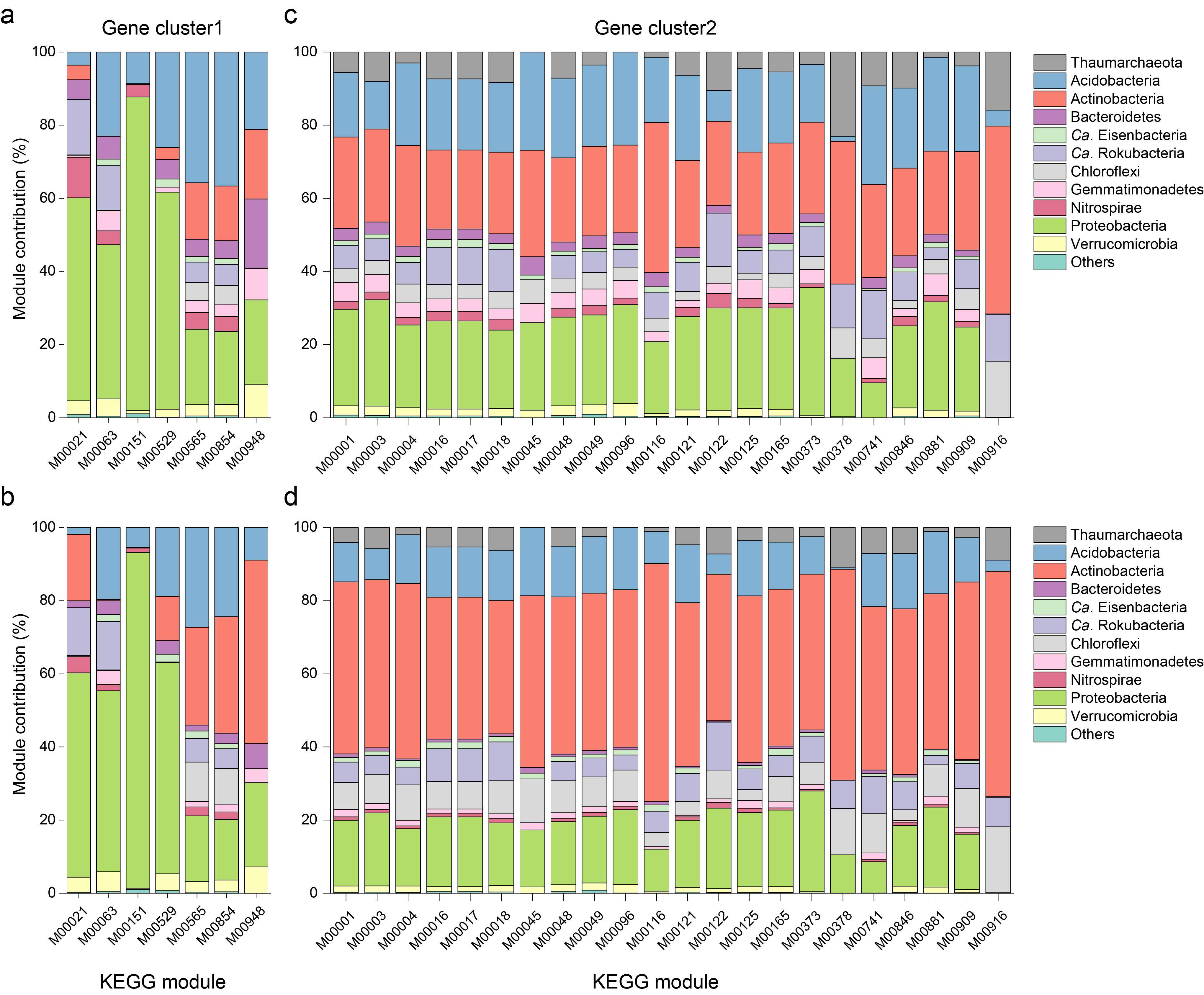


**Fig. S24:** Phylum-level assignment of KEGG modules in gene cluster 1 (a, b) and cluster 2 (c, d) under normal (a, c) and extreme summers (b, d). The relative abundance of each phylum was calculated by dividing the sum of RPKMs for a given module by the sum of RPKMs of all taxa.


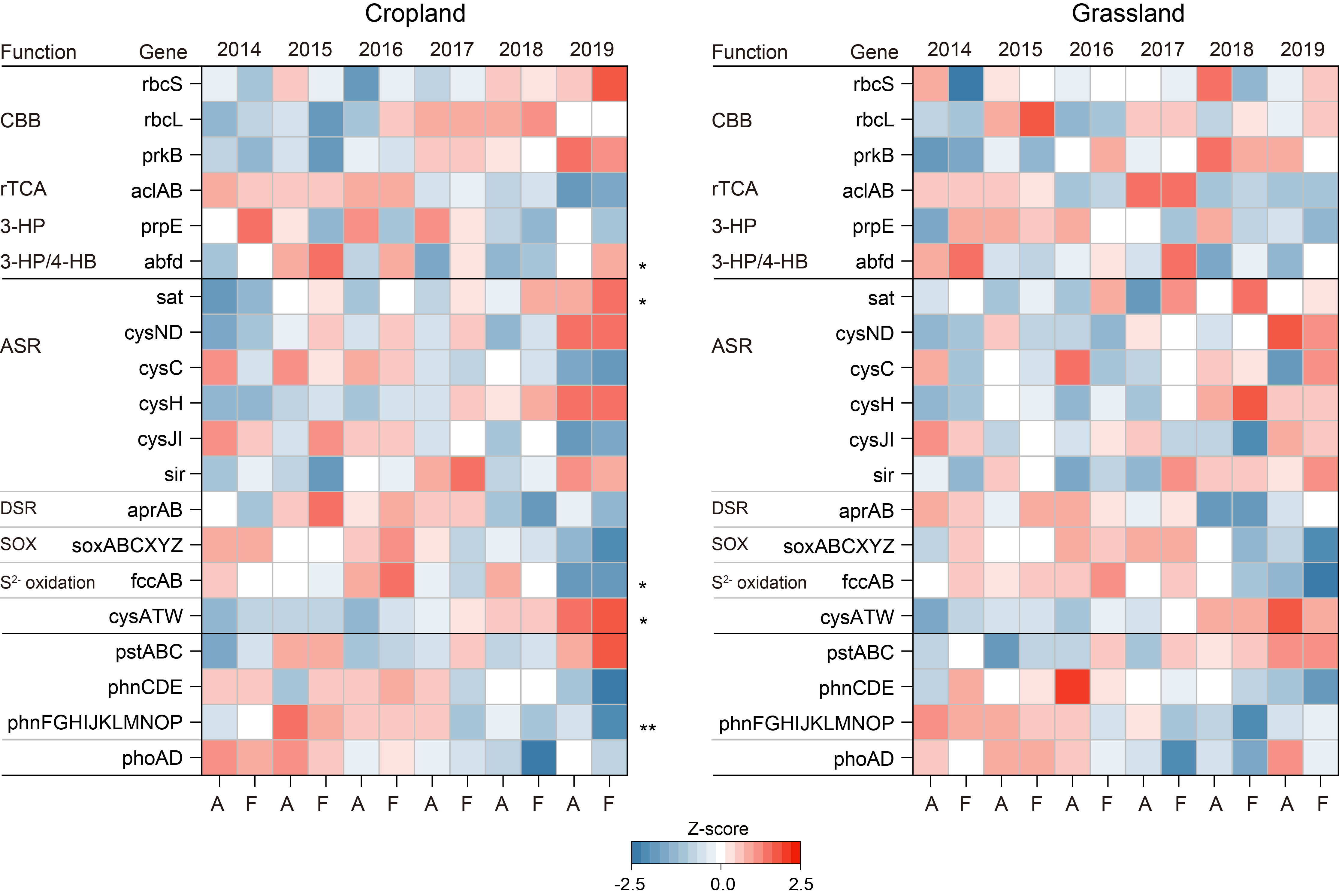


**Fig. S25:** Heatmap showing the abundance of key genes involved in microbial C, S, and P cycles as affected by climate change in cropland and grassland. Asterisks represent significant differences between A and F treatments based on the DESeq2 BH-adjusted *p* value (**p* < 0.05; ***p* < 0.01). The RPKM value of genes was log10 transformed before row scaling. See Appendix 1 for gene functions.


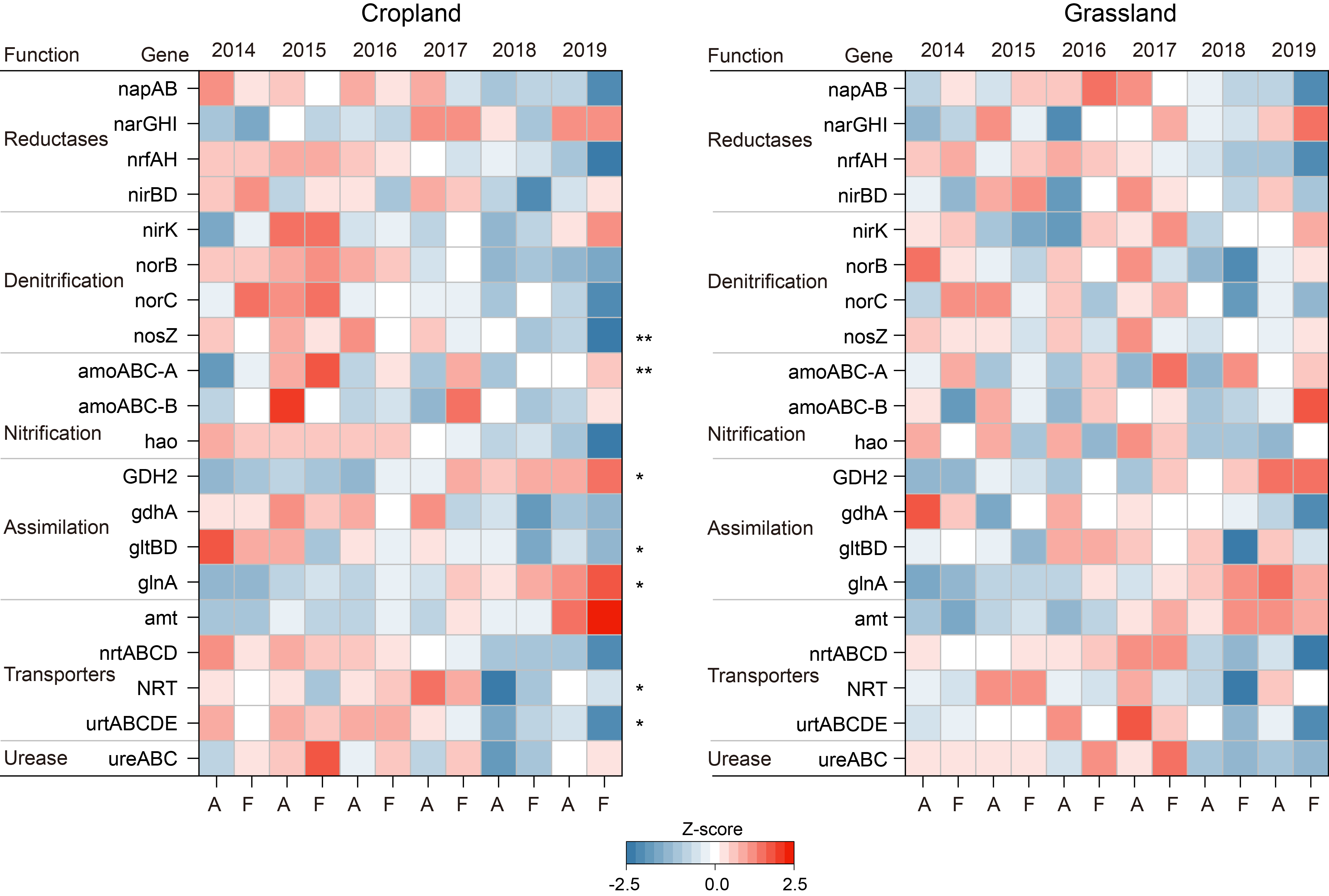


**Fig. S26:** Heatmap showing the key genes involved in microbial N cycle as affected by climate change in cropland and grassland. Asterisks represent significant differences between A and F treatments based on the DESeq2 BH-adjusted *p* value (**p* < 0.05; ***p* < 0.01). The RPKM value of genes was log10 transformed before row scaling. See Appendix 1 for gene functions.


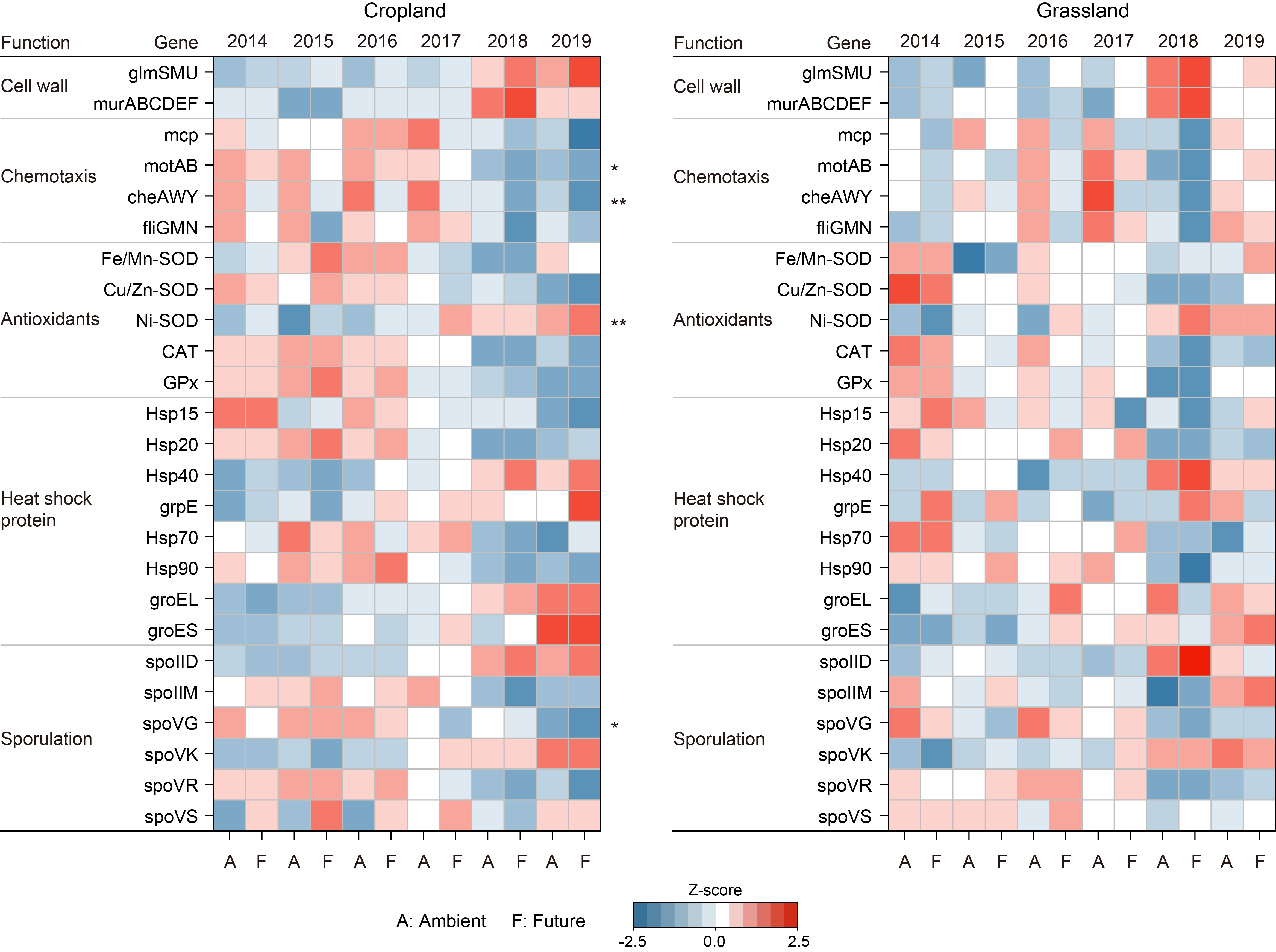


**Fig. S27:** Heatmap showing the key genes involved in microbial stress responses as affected by climate change in cropland and grassland. Asterisks represent significant differences between A and F treatments based on the DESeq2 BH-adjusted *p* value (**p* < 0.05; ***p* < 0.01). The RPKM value of genes was log10 transformed before row scaling. See Appendix 1 for gene functions.


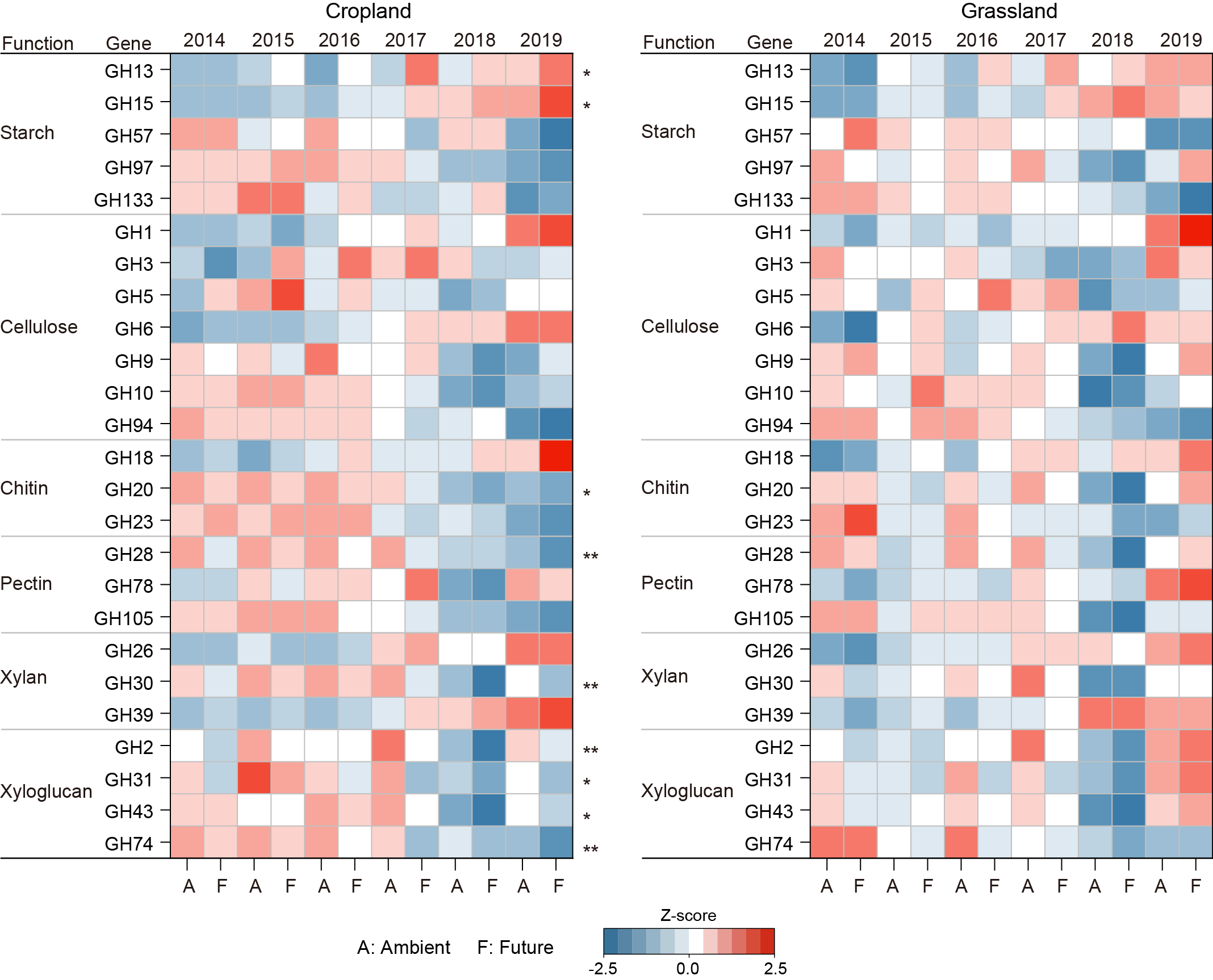


**Fig. S28:** Heatmap showing the key genes involved in CAZymes as affected by climate change in cropland and grassland. Asterisks represent significant differences between A and F treatments based on the DESeq2 BH-adjusted *p* value (**p* < 0.05; ***p* < 0.01). The RPKM value of genes was log10 transformed before row scaling (same for Fig. S28). See Appendix 2 for CAZyme GH functions.


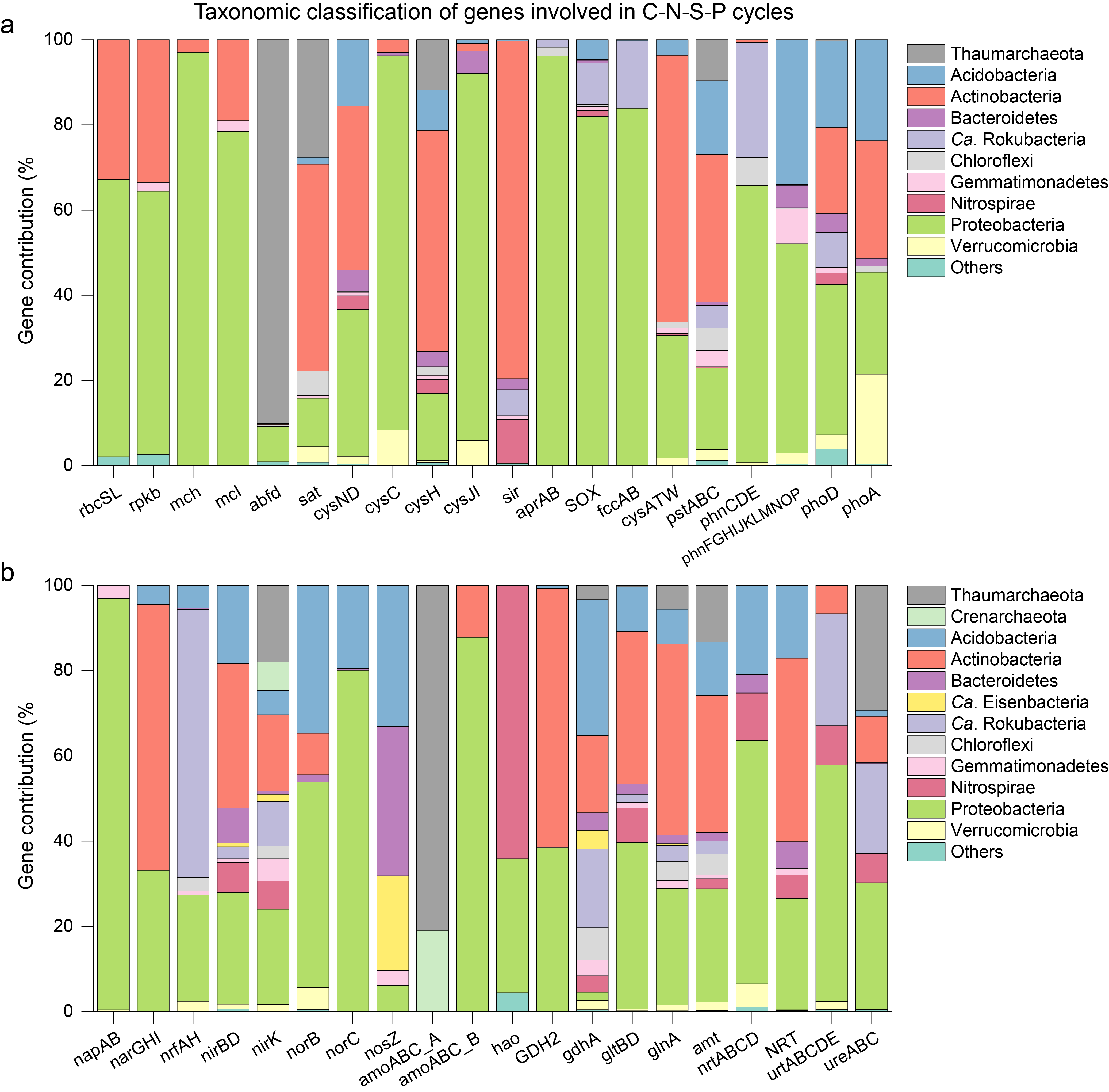


**Fig. S29:** Phylum-level assignment of genes involved in C, N, S, and P cycles. The relative abundance of each phylum was calculated by dividing the sum of RPKMs for a given taxon by the sum of RPKMs of all taxa (same for Fig. S30).


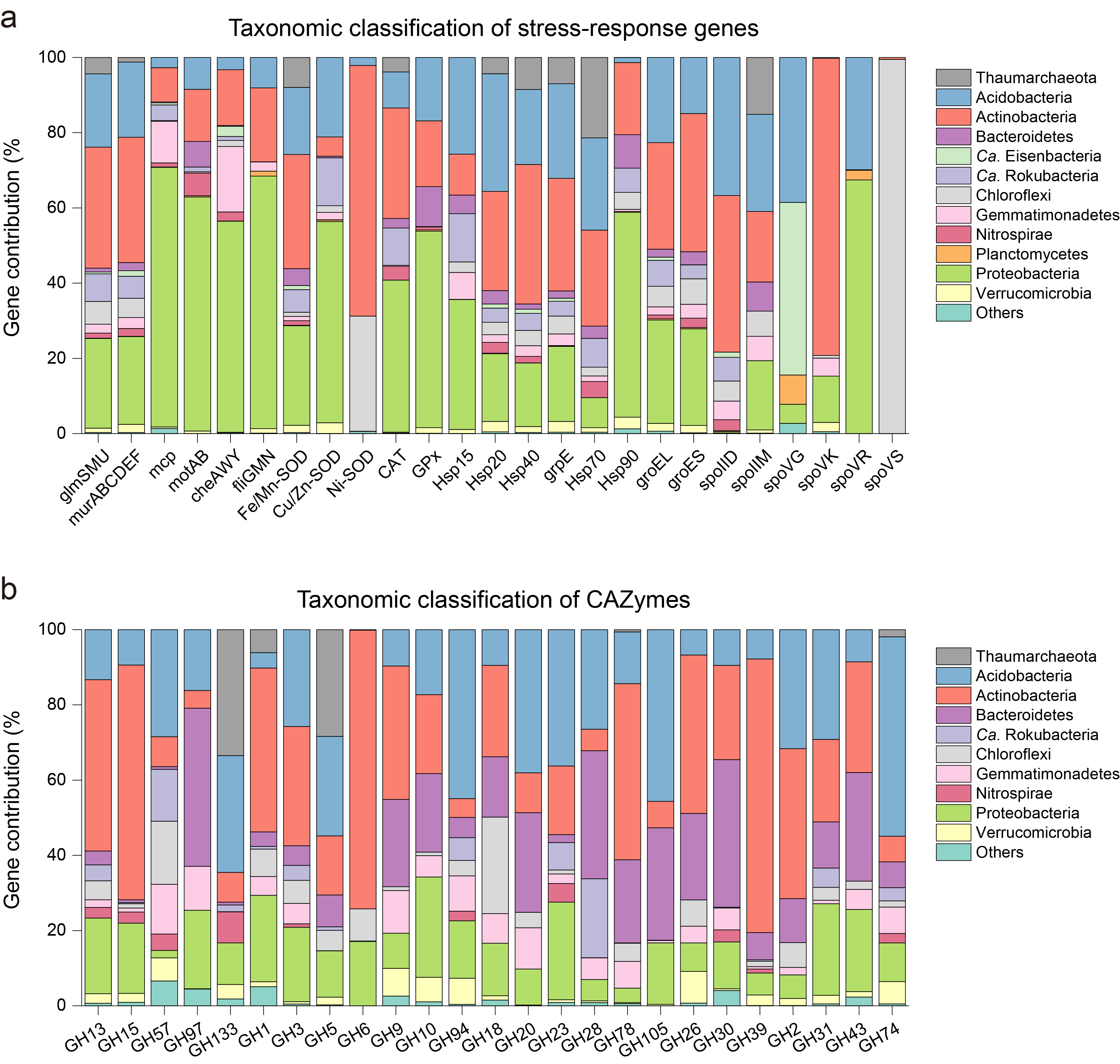


**Fig. S30:** Phylum-level assignment of stress-related genes and CAZymes.


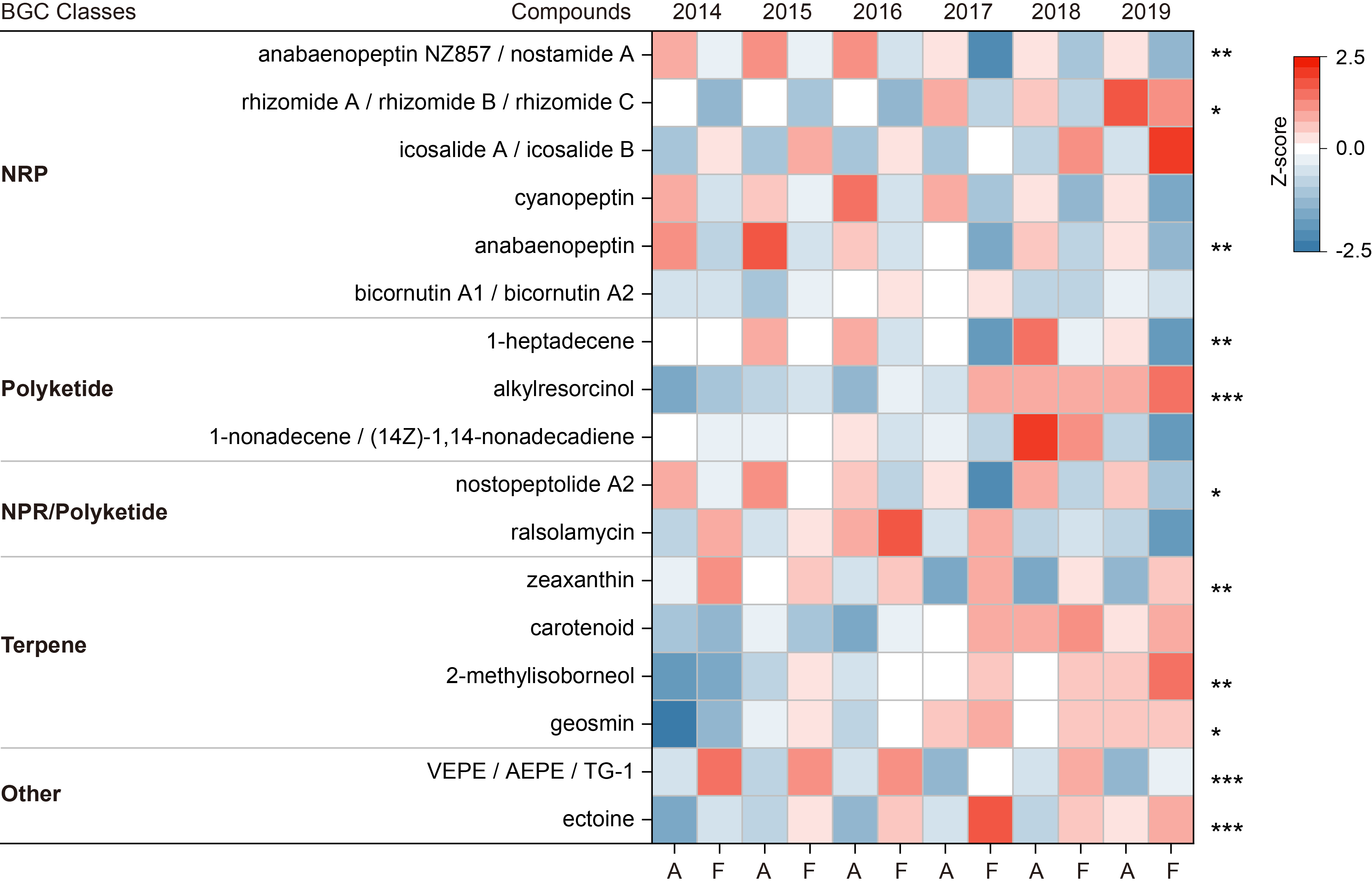


**Fig. S31:** Heatmap showing the abundance of secondary metabolite biosynthesis genes as affected by climate change during the summers of 2014–2019. Asterisks represent significant differences between A and F treatments based on the DESeq2 BH-adjusted *p* value (**p* < 0.05; ***p* < 0.01; ****p* < 0.001). The RPKM value of biosynthetic gene clusters (BGCs) was log10 transformed before row scaling.


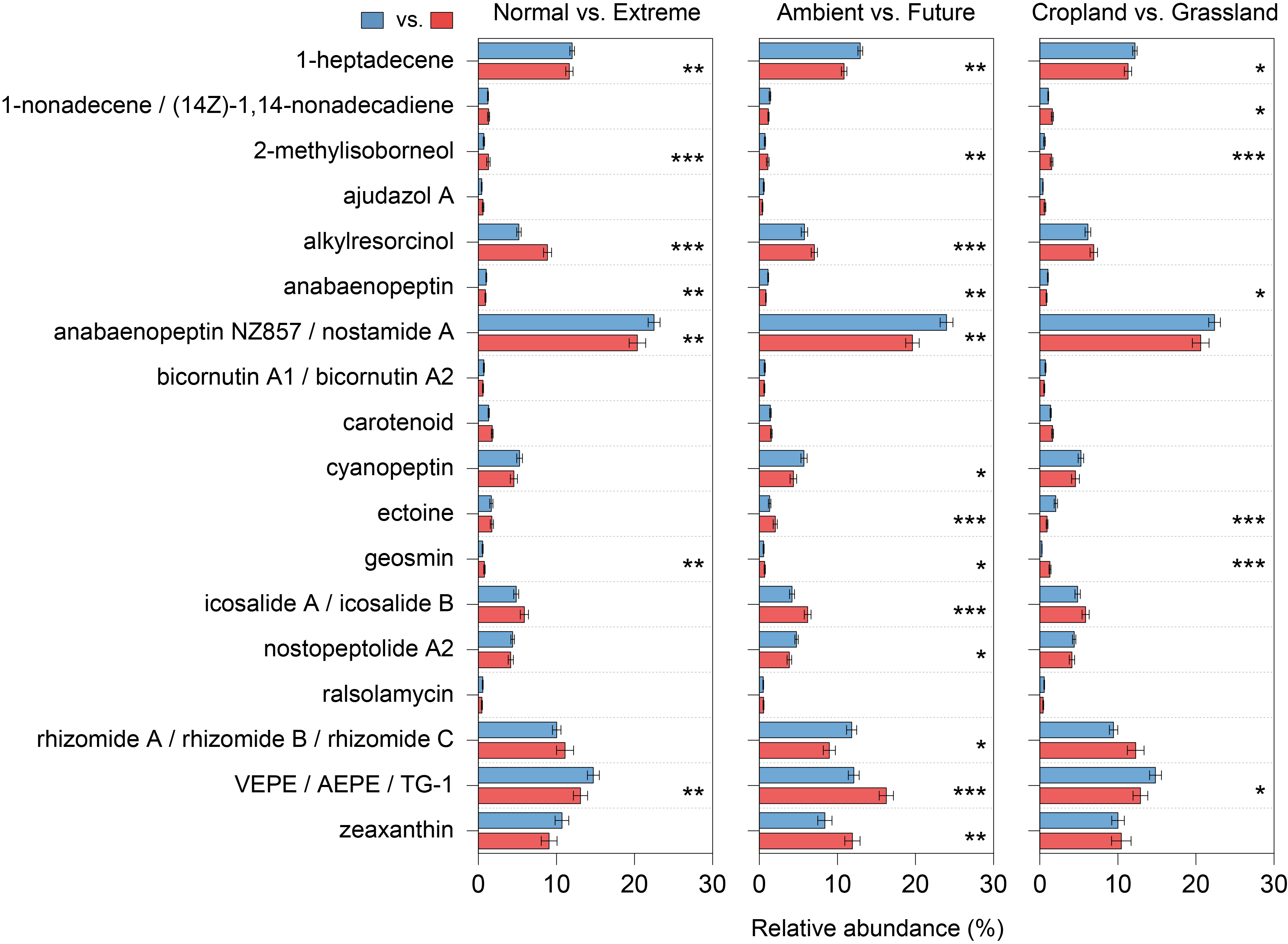


**Fig. S32:** Impact of climate and land-use changes on the abundance of secondary metabolite biosynthesis genes based on the contigs annotation with antiSMASH. The error bars show the standard error in each condition. Significantly altered phyla are marked with asterisks based on DESeq2 BH-adjusted *p* value, significance levels: **p* < 0.05; ***p* < 0.01; ****p* < 0.001.


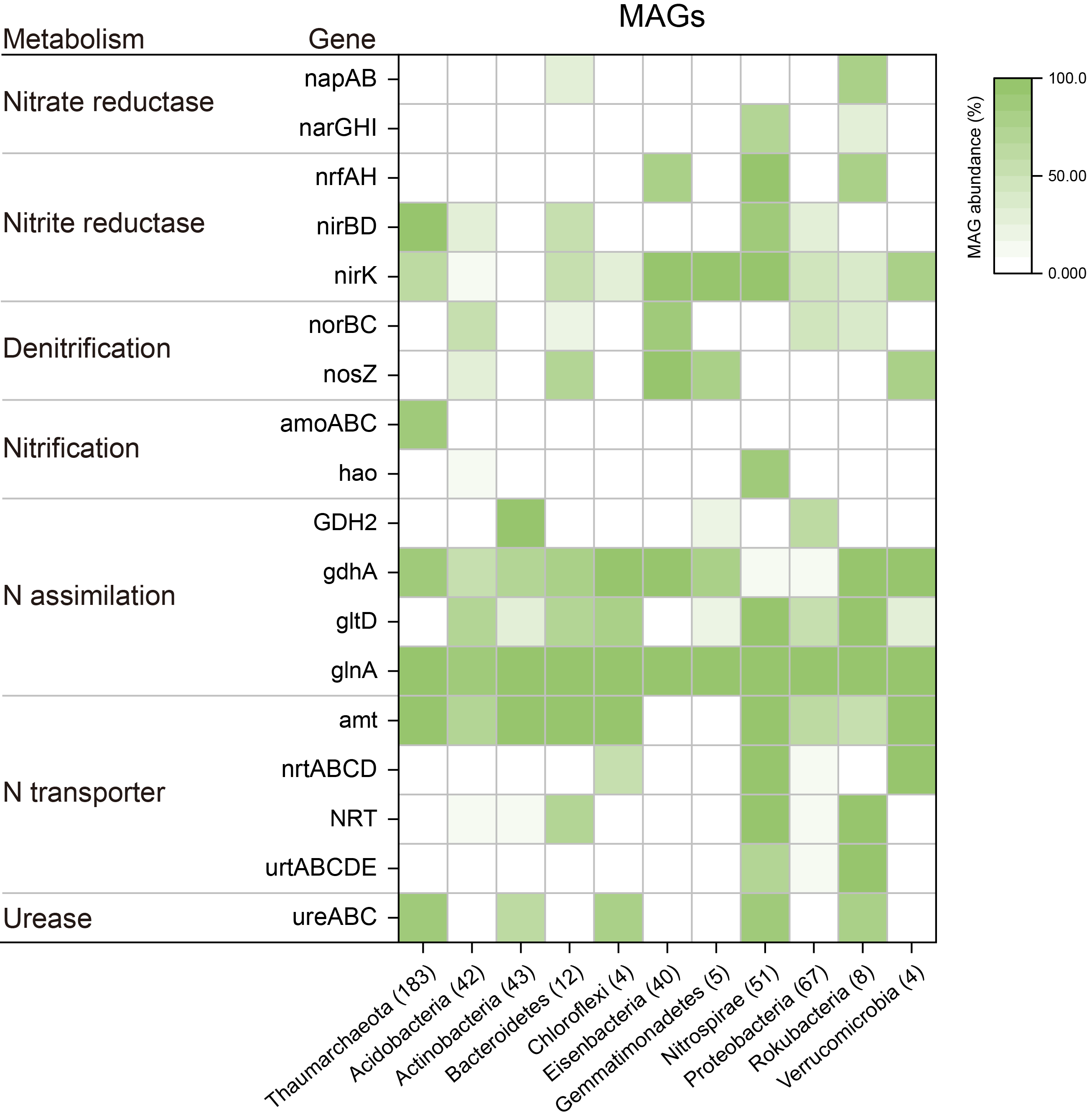


**Fig. S33:** The distribution of N cycling genes in metagenome assembled genomes (MAGs). Only the MAGs with > 70% completeness and < 10% contamination were selected (459 MAGs). Heatmap showing the presence of the N cycling genes across the MAGs spanning 11 phyla. Values in parentheses indicate the number of MAGs assigned to each phylum. For gene complexes, the highest gene frequency is shown.


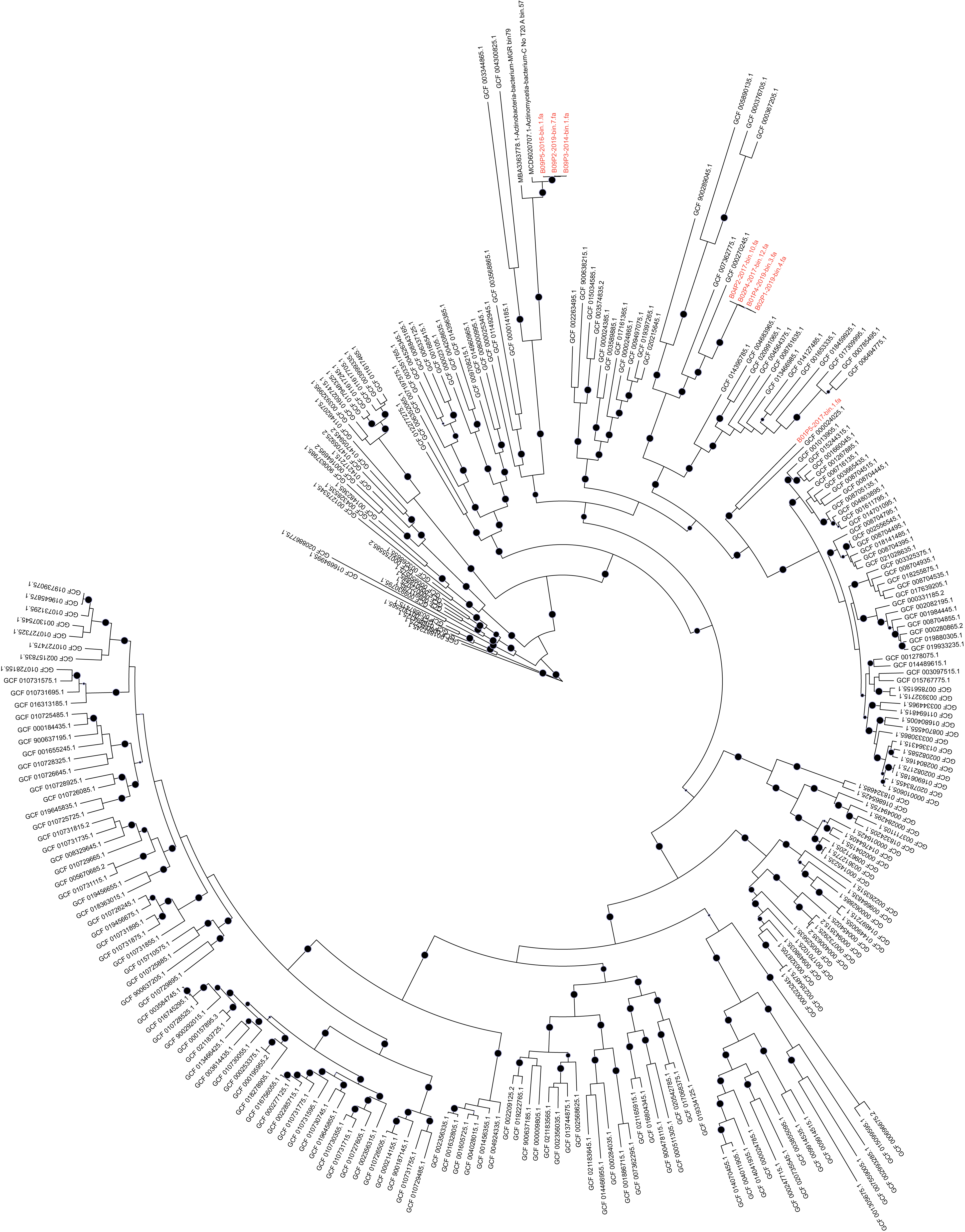


**Fig. S34:** Phylogenetic tree of *GDH2* genes identified in actinobacterial genomes from NCBI database and recovered MAGs. The tree was generated using a maximum likelihood-based approach in RAxML v8.2.12 with the PROTGAMMAWAG model [53]. Bootstrap values > 90 are shown. The accession number of actinobacterial genomes from the NCBI database is indicated in the tip. MAGs generated in our study were marked in red.

**Table S1.** Fertilization practices of three land-use types at the GCEF.

For more details please see Schädler et al. 2019 [5].

**Table S3.** Impact of future climate, extreme summers, land-use types, and their interactions on soil properties and microbial communities (LMMs analysis). *p* values < 0.05 in bold.

**Table S5.** Impact of future climate, extreme summers and land-use types on microbial communities (PERMANOVA analysis). *p* values < 0.05 in bold.

**References**

1. Rockel B, Will A, Hense A. The regional climate model COSMO-CLM (CCLM). Meteorol Z. 2008;17:347-348.

2. Jacob D, Podzun R. Sensitivity studies with the regional climate model REMO. Meteorol Atmos Phys. 1997;63:119-129.

3. Döscher R, Willén U, Jones C, Rutgersson A, Meier HM, Hansson U, et al. The development of the regional coupled ocean-atmosphere model RCAO. Boreal Environ Res. 2002;7:183.

4. Yin R, Siebert J, Eisenhauer N, Schädler M. Climate change and intensive land use reduce soil animal biomass via dissimilar pathways. Elife. 2020;9:e54749.

5. Schädler M, Buscot F, Klotz S, Reitz T, Durka W, Bumberger J, et al. Investigating the consequences of climate change under different land‐use regimes: a novel experimental infrastructure. Ecosphere. 2019;10:e02635.

6. Menzel P, Ng KL, Krogh A. Fast and sensitive taxonomic classification for metagenomics with Kaiju. Nat Commun. 2016;7:1-9.

7. Kopylova E, Noé L, Touzet H. SortMeRNA: fast and accurate filtering of ribosomal RNAs in metatranscriptomic data. Bioinformatics. 2012;28:3211-3217.

8. Quast C, Pruesse E, Yilmaz P, Gerken J, Schweer T, Yarza P, et al. The SILVA ribosomal RNA gene database project: improved data processing and web-based tools. Nucleic Acids Res. 2012;41:D590-D596.

9. Bay SK, Waite DW, Dong X, Gillor O, Chown SL, Hugenholtz P, et al. Chemosynthetic and photosynthetic bacteria contribute differentially to primary production across a steep desert aridity gradient. ISME J. 2021;15:3339-3356.

10. McMurdie PJ, Holmes S. phyloseq: an R package for reproducible interactive analysis and graphics of microbiome census data. PLoS One. 2013;8:e61217.

11. Guo J, Bolduc B, Zayed AA, Varsani A, Dominguez-Huerta G, Delmont TO, et al. VirSorter2: a multi-classifier, expert-guided approach to detect diverse DNA and RNA viruses. Microbiome. 2021;9:1-13.

12. Nayfach S, Camargo AP, Schulz F, Eloe-Fadrosh E, Roux S, Kyrpides NC. CheckV assesses the quality and completeness of metagenome-assembled viral genomes. Nat Biotechnol. 2021;39:578-585.

13. Nelson AR, Narrowe AB, Rhoades CC, Fegel TS, Daly RA, Roth HK, et al. Wildfire-dependent changes in soil microbiome diversity and function. Nat Microbiol. 2022;7:1419-1430.

14. Li Z, Pan D, Wei G, Pi W, Zhang C, Wang J-H, et al. Deep sea sediments associated with cold seeps are a subsurface reservoir of viral diversity. ISME J. 2021;15:2366-2378.

15. Roux S, Brum JR, Dutilh BE, Sunagawa S, Duhaime MB, Loy A, et al. Ecogenomics and potential biogeochemical impacts of globally abundant ocean viruses. Nature. 2016;537:689-693.

16. Fu L, Niu B, Zhu Z, Wu S, Li W. CD-HIT: accelerated for clustering the next-generation sequencing data. Bioinformatics. 2012;28:3150-3152.

17. Emerson JB, Roux S, Brum JR, Bolduc B, Woodcroft BJ, Jang HB, et al. Host-linked soil viral ecology along a permafrost thaw gradient. Nat Microbiol. 2018;3:870-880.

18. Buchfink B, Reuter K, Drost H-G. Sensitive protein alignments at tree-of-life scale using DIAMOND. Nat Methods. 2021;18:366-368.

19. Huson DH, Beier S, Flade I, Górska A, El-Hadidi M, Mitra S, et al. MEGAN community edition-interactive exploration and analysis of large-scale microbiome sequencing data. PLoS Comput Biol. 2016;12:e1004957.

20. Jian H, Yi Y, Wang J, Hao Y, Zhang M, Wang S, et al. Diversity and distribution of viruses inhabiting the deepest ocean on Earth. ISME J. 2021;15:3094-3110.

21. Shang J, Jiang J, Sun Y. Bacteriophage classification for assembled contigs using graph convolutional network. Bioinformatics. 2021;37:i25-i33.

22. Ji M, Fan X, Cornell CR, Zhang Y, Yuan MM, Tian Z, et al. Tundra soil viruses mediate responses of microbial communities to climate warming. Mbio. 2023:e03009-03022.

23. Trubl G, Kimbrel JA, Liquet-Gonzalez J, Nuccio EE, Weber PK, Pett-Ridge J, et al. Active virus-host interactions at sub-freezing temperatures in Arctic peat soil. Microbiome. 2021;9:1-15.

24. Johansen J, Plichta DR, Nissen JN, Jespersen ML, Shah SA, Deng L, et al. Genome binning of viral entities from bulk metagenomics data. Nat Commun. 2022;13:1-12.

25. Jain C, Rodriguez-R LM, Phillippy AM, Konstantinidis KT, Aluru S. High throughput ANI analysis of 90K prokaryotic genomes reveals clear species boundaries. Nat Commun. 2018;9:1-8.

26. Parada AE, Needham DM, Fuhrman JA. Every base matters: assessing small subunit rRNA primers for marine microbiomes with mock communities, time series and global field samples. Environ Microbiol. 2016;18:1403-1414.

27. Apprill A, McNally S, Parsons R, Weber L. Minor revision to V4 region SSU rRNA 806R gene primer greatly increases detection of SAR11 bacterioplankton. Aquat Microb Ecol. 2015;75:129-137.

28. White TJ, Bruns T, Lee S, Taylor J. Amplification and direct sequencing of fungal ribosomal RNA genes for phylogenetics. PCR protocols: a guide to methods and applications. 1990;18:315-322.

29. Amaral-Zettler LA, McCliment EA, Ducklow HW, Huse SM. A method for studying protistan diversity using massively parallel sequencing of V9 hypervariable regions of small-subunit ribosomal RNA genes. PLoS One. 2009;4:e6372.

30. Stoeck T, Bass D, Nebel M, Christen R, Jones MD, BREINER HW, et al. Multiple marker parallel tag environmental DNA sequencing reveals a highly complex eukaryotic community in marine anoxic water. Mol Ecol. 2010;19:21-31.

31. Gilbert JA, Jansson JK, Knight R. The Earth Microbiome project: successes and aspirations. BMC Biol. 2014;12:1-4.

32. Edgar RC. Search and clustering orders of magnitude faster than BLAST. Bioinformatics. 2010;26:2460-2461.

33. Bei Q, Moser G, Wu X, Müller C, Liesack W. Metatranscriptomics reveals climate change effects on the rhizosphere microbiomes in European grassland. Soil Biol Biochem. 2019;138:107604.

34. Bolger AM, Lohse M, Usadel B. Trimmomatic: a flexible trimmer for Illumina sequence data. Bioinformatics. 2014;30:2114-2120.

35. Bay SK, Dong X, Bradley JA, Leung PM, Grinter R, Jirapanjawat T, et al. Trace gas oxidizers are widespread and active members of soil microbial communities. Nat Microbiol. 2021;6:246-256.

36. Li D, Liu C-M, Luo R, Sadakane K, Lam T-W. MEGAHIT: an ultra-fast single-node solution for large and complex metagenomics assembly via succinct *de Bruijn* graph. Bioinformatics. 2015;31:1674-1676.

37. Uritskiy GV, DiRuggiero J, Taylor J. MetaWRAP—a flexible pipeline for genome-resolved metagenomic data analysis. Microbiome. 2018;6:1-13.

38. Wolf YI, Kazlauskas D, Iranzo J, Lucía-Sanz A, Kuhn JH, Krupovic M, et al. Origins and evolution of the global RNA virome. MBio. 2018;9:e02329-02318.

39. Ning D, Yuan M, Wu L, Zhang Y, Guo X, Zhou X, et al. A quantitative framework reveals ecological drivers of grassland microbial community assembly in response to warming. Nat Commun. 2020;11:4717.

40. Zhou J, Ning D. Stochastic community assembly: does it matter in microbial ecology? Microbiol Mol Biol Rev. 2017;81:e00002-00017.

41. Stegen JC, Lin X, Fredrickson JK, Chen X, Kennedy DW, Murray CJ, et al. Quantifying community assembly processes and identifying features that impose them. ISME J. 2013;7:2069-2079.

42. Kumar L, Futschik ME. Mfuzz: a software package for soft clustering of microarray data. Bioinformation. 2007;2:5.

43. Seitz VA, McGivern BB, Daly RA, Chaparro JM, Borton MA, Sheflin AM, et al. Variation in root exudate composition influences soil microbiome membership and function. Appl Environ Microbiol. 2021:e00226-00222.

44. Rogers TJ, Buongiorno J, Jessen GL, Schrenk MO, Fordyce JA, de Moor JM, et al. Chemolithoautotroph distributions across the subsurface of a convergent margin. ISME J. 2022:1-11.

45. Harth G, Zamecnik PC, Tang J-Y, Tabatadze D, Horwitz MA. Treatment of *Mycobacterium tuberculosis* with antisense oligonucleotides to glutamine synthetase mRNA inhibits glutamine synthetase activity, formation of the poly-L-glutamate/glutamine cell wall structure, and bacterial replication. Proc Natl Acad Sci USA. 2000;97:418-423.

46. Harth G, Masleša‐Galić S, Tullius MV, Horwitz MA. All four *Mycobacterium tuberculosis* *glnA* genes encode glutamine synthetase activities but only GlnA1 is abundantly expressed and essential for bacterial homeostasis. Mol Microbiol. 2005;58:1157-1172.

47. Nolden L, Farwick M, Krämer R, Burkovski A. Glutamine synthetases of *Corynebacterium glutamicum*: transcriptional control and regulation of activity. FEMS Microbiol Lett. 2001;201:91-98.

48. Rakovitsky N, Bar Oz M, Goldberg K, Gibbons S, Zimhony O, Barkan D. The unexpected essentiality of *glnA2* in *Mycobacterium smegmatis* is salvaged by overexpression of the global nitrogen regulator *glnR*, but not by L-, D-or iso-glutamine. Front Microbiol. 2018:2143.

49. Krysenko S, Matthews A, Okoniewski N, Kulik A, Girbas MG, Tsypik O, et al. Initial metabolic step of a novel ethanolamine utilization pathway and its regulation in *Streptomyces coelicolor* M145. Mbio. 2019;10:e00326-00319.

50. Krysenko S, Okoniewski N, Kulik A, Matthews A, Grimpo J, Wohlleben W, et al. Gamma-glutamylpolyamine synthetase GlnA3 is involved in the first step of polyamine degradation pathway in *Streptomyces coelicolor* M145. Front Microbiol. 2017;8:726.

51. Amon J, Titgemeyer F, Burkovski A. Common patterns–unique features: nitrogen metabolism and regulation in Gram-positive bacteria. FEMS Microbiol Rev. 2010;34:588-605.

52. Harper CJ, Hayward D, Kidd M, Wiid I, Van Helden P. Glutamate dehydrogenase and glutamine synthetase are regulated in response to nitrogen availability in *Myocbacterium smegmatis*. BMC Microbiol. 2010;10:1-12.

53. Stamatakis A. RAxML version 8: a tool for phylogenetic analysis and post-analysis of large phylogenies. Bioinformatics. 2014;30:1312-1313.

**Appendix 1**

|  | **KO** | **Gene** | **Function** |
| --- | --- | --- | --- |
| **C-cycle** | K01602 | rbcS | rbcS, cbbS; ribulose-bisphosphate carboxylase small chain [EC:4.1.1.39] |
|  | K01601 | rbcL | rbcL, cbbL; ribulose-bisphosphate carboxylase large chain [EC:4.1.1.39] |
|  | K00855 | prkB | PRK, prkB; phosphoribulokinase [EC:2.7.1.19] |
|  | K15230 | aclA | aclA; ATP-citrate lyase alpha-subunit [EC:2.3.3.8] |
|  | K15231 | aclB | aclB; ATP-citrate lyase beta-subunit [EC:2.3.3.8] |
|  | K01908 | prpE | ACSS3, prpE; propionyl-CoA synthetase [EC:6.2.1.17] |
|  | K14534 | abfD | abfD; 4-hydroxybutyryl-CoA dehydratase / vinylacetyl-CoA-Delta-isomerase [EC:4.2.1.120 5.3.3.3] |
| **S-cycle** | K00958 | sat | sat, met3; sulfate adenylyltransferase [EC:2.7.7.4] |
|  | K00956 | cysN | cysN; sulfate adenylyltransferase subunit 1 [EC:2.7.7.4] |
|  | K00957 | cysD | cysD; sulfate adenylyltransferase subunit 2 [EC:2.7.7.4] |
|  | K00860 | cysC | cysC; adenylylsulfate kinase [EC:2.7.1.25] |
|  | K00390 | cysH | cysH; phosphoadenosine phosphosulfate reductase [EC:1.8.4.8 1.8.4.10] |
|  | K00392 | sir | sir; sulfite reductase (ferredoxin) [EC:1.8.7.1] |
|  | K00394 | aprA | aprA; adenylylsulfate reductase, subunit A [EC:1.8.99.2] |
|  | K00395 | aprB | aprB; adenylylsulfate reductase, subunit B [EC:1.8.99.2] |
|  | K17222 | soxA | soxA; L-cysteine S-thiosulfotransferase [EC:2.8.5.2] |
|  | K17224 | soxB | soxB; S-sulfosulfanyl-L-cysteine sulfohydrolase [EC:3.1.6.20] |
|  | K17225 | soxC | soxC; sulfane dehydrogenase subunit SoxC |
|  | K17226 | soxY | soxY; sulfur-oxidizing protein SoxY |
|  | K17223 | soxX | soxX; L-cysteine S-thiosulfotransferase [EC:2.8.5.2] |
|  | K17227 | soxZ | soxZ; sulfur-oxidizing protein SoxZ |
|  | K17229 | fccB | fccB; sulfide dehydrogenase [flavocytochrome c] flavoprotein chain [EC:1.8.2.3] |
|  | K17230 | fccA | fccA; cytochrome subunit of sulfide dehydrogenase |
| **P-cycle** | K02045 | cysA | cysA; sulfate/thiosulfate transport system ATP-binding protein [EC:7.3.2.3] |
|  | K02046 | cysU | cysU; sulfate/thiosulfate transport system permease protein |
|  | K02047 | cysW | cysW; sulfate/thiosulfate transport system permease protein |
|  | K02036 | pstB | pstB; phosphate transport system ATP-binding protein [EC:7.3.2.1] |
|  | K02037 | pstC | pstC; phosphate transport system permease protein |
|  | K02038 | pstA | pstA; phosphate transport system permease protein |
|  | K02041 | phnC | phnC; phosphonate transport system ATP-binding protein [EC:7.3.2.2] |
|  | K02042 | phnE | phnE; phosphonate transport system permease protein |
|  | K02044 | phnD | phnD; phosphonate transport system substrate-binding protein |
|  | K02043 | phnF | phnF; GntR family transcriptional regulator, phosphonate transport system regulatory protein |
|  | K06166 | phnG | phnG; alpha-D-ribose 1-methylphosphonate 5-triphosphate synthase subunit PhnG [EC:2.7.8.37] |
|  | K06165 | phnH | phnH; alpha-D-ribose 1-methylphosphonate 5-triphosphate synthase subunit PhnH [EC:2.7.8.37] |
|  | K06164 | phnI | phnI; alpha-D-ribose 1-methylphosphonate 5-triphosphate synthase subunit PhnI [EC:2.7.8.37] |
|  | K06163 | phnJ | phnJ; alpha-D-ribose 1-methylphosphonate 5-phosphate C-P lyase [EC:4.7.1.1] |
|  | K05781 | phnK | phnK; putative phosphonate transport system ATP-binding protein |
|  | K05780 | phnL | phnL; alpha-D-ribose 1-methylphosphonate 5-triphosphate synthase subunit PhnL [EC:2.7.8.37] |
|  | K06162 | phnM | phnM; alpha-D-ribose 1-methylphosphonate 5-triphosphate diphosphatase [EC:3.6.1.63] |
|  | K05774 | phnN | phnN; ribose 1,5-bisphosphokinase [EC:2.7.4.23] |
|  | K09994 | phnO | phnO; (aminoalkyl)phosphonate N-acetyltransferase [EC:2.3.1.280] |
|  | K06167 | phnP | phnP; phosphoribosyl 1,2-cyclic phosphate phosphodiesterase [EC:3.1.4.55] |
|  | K01077 | phoA | phoA, phoB; alkaline phosphatase [EC:3.1.3.1] |
|  | K01113 | phoD | phoD; alkaline phosphatase D [EC:3.1.3.1] |
| **N-cycle** | K02567 | napA | napA; nitrate reductase (cytochrome) [EC:1.9.6.1] |
|  | K02568 | napB | napB; nitrate reductase (cytochrome), electron transfer subunit |
|  | K00370 | narG | narG, narZ, nxrA; nitrate reductase / nitrite oxidoreductase, alpha subunit [EC:1.7.5.1 1.7.99.-] |
|  | K00371 | narH | narH, narY, nxrB; nitrate reductase / nitrite oxidoreductase, beta subunit [EC:1.7.5.1 1.7.99.-] |
|  | K00374 | narI | narI, narV; nitrate reductase gamma subunit [EC:1.7.5.1 1.7.99.-] |
|  | K03385 | nrfA | nrfA; nitrite reductase (cytochrome c-552) [EC:1.7.2.2] |
|  | K15876 | nrfH | nrfH; cytochrome c nitrite reductase small subunit |
|  | K00362 | nirB | nirB; nitrite reductase (NADH) large subunit [EC:1.7.1.15] |
|  | K00363 | nirD | nirD; nitrite reductase (NADH) small subunit [EC:1.7.1.15] |
|  | K00368 | nirK | nirK; nitrite reductase (NO-forming) [EC:1.7.2.1] |
|  | K02305 | norC | norC; nitric oxide reductase subunit C |
|  | K04561 | norB | norB; nitric oxide reductase subunit B [EC:1.7.2.5] |
|  | K00376 | nosZ | nosZ; nitrous-oxide reductase [EC:1.7.2.4] |
|  | K10944 | amoA | pmoA-amoA; methane/ammonia monooxygenase subunit A [EC:1.14.18.3 1.14.99.39] |
|  | K10945 | amoB | pmoB-amoB; methane/ammonia monooxygenase subunit B |
|  | K10946 | amoC | pmoC-amoC; methane/ammonia monooxygenase subunit C |
|  | K10535 | hao | hao; hydroxylamine dehydrogenase [EC:1.7.2.6] |
|  | K15371 | GDH2 | GDH2; glutamate dehydrogenase [EC:1.4.1.2] |
|  | K00261 | gdhA | GLUD1_2, gdhA; glutamate dehydrogenase (NAD(P)+) [EC:1.4.1.3] |
|  | K00262 | gdhA | gdhA; glutamate dehydrogenase (NADP+) [EC:1.4.1.4] |
|  | K00265 | gltB | gltB; glutamate synthase (NADPH) large chain [EC:1.4.1.13] |
|  | K00266 | gltD | gltD; glutamate synthase (NADPH) small chain [EC:1.4.1.13] |
|  | K01915 | glnA | glnA, GLUL; glutamine synthetase [EC:6.3.1.2] |
|  | K03320 | amt | amt, AMT, MEP; ammonium transporter, Amt family |
|  | K15576 | nrtA | nrtA, nasF, cynA; nitrate/nitrite transport system substrate-binding protein |
|  | K15577 | nrtB | nrtB, nasE, cynB; nitrate/nitrite transport system permease protein |
|  | K15578 | nrtC | nrtC, nasD; nitrate/nitrite transport system ATP-binding protein [EC:7.3.2.4] |
|  | K15579 | nrtD | nrtD, cynD; nitrate/nitrite transport system ATP-binding protein |
|  | K02575 | NRT | NRT, narK, nrtP, nasA; MFS transporter, NNP family, nitrate/nitrite transporter |
|  | K11959 | urtA | urtA; urea transport system substrate-binding protein |
|  | K11960 | urtB | urtB; urea transport system permease protein |
|  | K11961 | urtC | urtC; urea transport system permease protein |
|  | K11962 | urtD | urtD; urea transport system ATP-binding protein |
|  | K11963 | urtE | urtE; urea transport system ATP-binding protein |
|  | K01428 | ureC | ureC; urease subunit alpha [EC:3.5.1.5] |
|  | K01429 | ureB | ureB; urease subunit beta [EC:3.5.1.5] |
|  | K01430 | ureA | ureA; urease subunit gamma [EC:3.5.1.5] |
| **Stress**  **related** | K00820 | glmS | glmS, GFPT; glutamine---fructose-6-phosphate transaminase (isomerizing) [EC:2.6.1.16] |
|  | K04042 | glmU | glmU; bifunctional UDP-N-acetylglucosamine pyrophosphorylase / glucosamine-1-phosphate N-acetyltransferase [EC:2.7.7.23 2.3.1.157] |
|  | K03431 | glmM | glmM; phosphoglucosamine mutase [EC:5.4.2.10] |
|  | K00790 | murA | murA; UDP-N-acetylglucosamine 1-carboxyvinyltransferase [EC:2.5.1.7] |
|  | K00075 | murB | murB; UDP-N-acetylmuramate dehydrogenase [EC:1.3.1.98] |
|  | K01924 | murC | murC; UDP-N-acetylmuramate--alanine ligase [EC:6.3.2.8] |
|  | K01925 | murD | murD; UDP-N-acetylmuramoylalanine--D-glutamate ligase [EC:6.3.2.9] |
|  | K01928 | murE | murE; UDP-N-acetylmuramoyl-L-alanyl-D-glutamate--2,6-diaminopimelate ligase [EC:6.3.2.13] |
|  | K01929 | murF | murF; UDP-N-acetylmuramoyl-tripeptide--D-alanyl-D-alanine ligase [EC:6.3.2.10] |
|  | K03406 | mcp | mcp; methyl-accepting chemotaxis protein |
|  | K02556 | motA | motA; chemotaxis protein MotA |
|  | K02557 | motB | motB; chemotaxis protein MotB |
|  | K03407 | cheA | cheA; two-component system, chemotaxis family, sensor kinase CheA [EC:2.7.13.3] |
|  | K03408 | cheW | cheW; purine-binding chemotaxis protein CheW |
|  | K03413 | cheY | cheY; two-component system, chemotaxis family, chemotaxis protein CheY |
|  | K02410 | fliG | fliG; flagellar motor switch protein FliG |
|  | K02416 | fliM | fliM; flagellar motor switch protein FliM |
|  | K02417 | fliN | fliN; flagellar motor switch protein FliN |
|  | K04564 | Fe-Mn-SOD | SOD2; superoxide dismutase, Fe-Mn family [EC:1.15.1.1] |
|  | K04565 | Cu-Zn-SOD | SOD1; superoxide dismutase, Cu-Zn family [EC:1.15.1.1] |
|  | K00518 | NiSOD | sodN; nickel superoxide dismutase [EC:1.15.1.1] |
|  | K03781 | CAT | katE, CAT, catB, srpA; catalase [EC:1.11.1.6] |
|  | K00432 | GPx | gpx, btuE, bsaA; glutathione peroxidase [EC:1.11.1.9] |
|  | K04762 | Hsp15 | hslR; ribosome-associated heat shock protein Hsp15 |
|  | K13993 | Hsp20 | HSP20; HSP20 family protein |
|  | K03686 | Hsp40 | dnaJ; molecular chaperone DnaJ |
|  | K03687 | GrpE | GRPE; molecular chaperone GrpE |
|  | K04043 | Hsp70 | dnaK, HSPA9; molecular chaperone DnaK |
|  | K04079 | Hsp90 | HSP90A, htpG; molecular chaperone HtpG |
|  | K04077 | groEL | groEL, HSPD1; chaperonin GroEL [EC:5.6.1.7] |
|  | K04078 | groES | groES, HSPE1; chaperonin GroES |
|  | K06381 | spoIID | spoIID; stage II sporulation protein D |
|  | K06384 | spoIIM | spoIIM; stage II sporulation protein M |
|  | K06412 | spoVG | spoVG; stage V sporulation protein G |
|  | K06413 | spoVK | spoVK; stage V sporulation protein K |
|  | K06415 | spoVR | spoVR; stage V sporulation protein R |
|  | K06416 | spoVS | spoVS; stage V sporulation protein S |

(https://www.kegg.jp/)

**Appendix 2**

| **GH family** | **Activities in Family** |
| --- | --- |
| **GH13** | alpha-amylase (EC 3.2.1.1); pullulanase (EC 3.2.1.41); cyclomaltodextrin glucanotransferase (EC 2.4.1.19); cyclomaltodextrinase (EC 3.2.1.54); trehalose-6-phosphate hydrolase (EC 3.2.1.93); oligo-alpha-glucosidase (EC 3.2.1.10); maltogenic amylase (EC 3.2.1.133); neopullulanase (EC 3.2.1.135); alpha-glucosidase (EC 3.2.1.20); maltotetraose-forming alpha-amylase (EC 3.2.1.60); isoamylase (EC 3.2.1.68); glucodextranase (EC 3.2.1.70); maltohexaose-forming alpha-amylase (EC 3.2.1.98); maltotriose-forming alpha-amylase (EC 3.2.1.116); branching enzyme (EC 2.4.1.18); trehalose synthase (EC 5.4.99.16); 4-alpha-glucanotransferase (EC 2.4.1.25); maltopentaose-forming alpha-amylase (EC 3.2.1.-) ; amylosucrase (EC 2.4.1.4) ; sucrose phosphorylase (EC 2.4.1.7); malto-oligosyltrehalose trehalohydrolase (EC 3.2.1.141); isomaltulose synthase (EC 5.4.99.11); malto-oligosyltrehalose synthase (EC 5.4.99.15); amylo-alpha-1,6-glucosidase (EC 3.2.1.33); alpha-1,4-glucan: phosphate alpha-maltosyltransferase (EC 2.4.99.16); amino acid transporter; [retaining] sucrose 6(F)-phosphate phosphorylase (EC 2.4.1.329); [retaining] glucosylglycerol phosphorylase (EC 2.4.1.359); ; Glucosylglycerate phosphorylase (EC 2.4.1.352); [retaining] sucrose alpha-glucosidase (EC 3.2.1.48); oligosaccharide alpha-4-glucosyltransferase (EC 2.4.1.161) |
| **GH15** | glucoamylase (EC 3.2.1.3); glucodextranase (EC 3.2.1.70); alpha,alpha-trehalase (EC 3.2.1.28); dextran dextrinase (EC 2.4.1.2) |
| **GH57** | alpha-amylase (EC 3.2.1.1); alpha-galactosidase (EC 3.2.1.22); amylopullulanase (EC 3.2.1.41); cyclomaltodextrinase (EC 3.2.1.54); branching enzyme (EC 2.4.1.18); 4-alpha-glucanotransferase (EC 2.4.1.25) |
| **GH97** | glucoamylase (EC 3.2.1.3); alpha-glucosidase (EC 3.2.1.20); alpha-galactosidase (EC 3.2.1.22) |
| **GH133** | amylo-alpha-1,6-glucosidase (EC 3.2.1.33); |
| **GH1** | beta-glucosidase (EC 3.2.1.21); beta-galactosidase (EC 3.2.1.23); beta-mannosidase (EC 3.2.1.25); beta-glucuronidase (EC 3.2.1.31); beta-xylosidase (EC 3.2.1.37); beta-D-fucosidase (EC 3.2.1.38); phlorizin hydrolase (EC 3.2.1.62); exo-beta-1,4-glucanase (EC 3.2.1.74); 6-phospho-beta-galactosidase (EC 3.2.1.85); 6-phospho-beta-glucosidase (EC 3.2.1.86); strictosidine beta-glucosidase (EC 3.2.1.105); lactase (EC 3.2.1.108); amygdalin beta-glucosidase (EC 3.2.1.117); prunasin beta-glucosidase (EC 3.2.1.118); vicianin hydrolase (EC 3.2.1.119); raucaffricine beta-glucosidase (EC 3.2.1.125); thioglucosidase (EC 3.2.1.147); beta-primeverosidase (EC 3.2.1.149); isoflavonoid 7-O-beta-apiosyl-beta-glucosidase (EC 3.2.1.161); ABA-specific beta-glucosidase (EC 3.2.1.175); DIMBOA beta-glucosidase (EC 3.2.1.182); beta-glycosidase (EC 3.2.1.-); hydroxyisourate hydrolase (EC 3.-.-.-); beta-rutinosidase /alpha-L-rhamnose-(1,6)-beta-D-glucosidase (EC 3.2.1.-) |
| **GH3** | beta-glucosidase (EC 3.2.1.21); xylan 1,4-beta-xylosidase (EC 3.2.1.37); beta-glucosylceramidase (EC 3.2.1.45); beta-N-acetylhexosaminidase (EC 3.2.1.52); alpha-L-arabinofuranosidase (EC 3.2.1.55); glucan 1,4-beta-glucosidase (EC 3.2.1.74); isoprimeverose-producing oligoxyloglucan hydrolase (EC 3.2.1.120); coniferin beta-glucosidase (EC 3.2.1.126); exo-1,3-1,4-glucanase (EC 3.2.1.-); beta-N-acetylglucosaminide phosphorylases (EC 2.4.1.-); beta-1,2-glucosidase (EC 3.2.1.-); beta-1,3-glucosidase (EC 3.2.1.-); xyloglucan-specific exo-beta-1,4-glucanase / exo-xyloglucanase (EC 3.2.1.155) |
| **GH5** | endo-beta-1,4-glucanase / cellulase (EC 3.2.1.4); endo-beta-1,4-xylanase (EC 3.2.1.8); beta-glucosidase (EC 3.2.1.21); beta-mannosidase (EC 3.2.1.25); beta-glucosylceramidase (EC 3.2.1.45); glucan beta-1,3-glucosidase (EC 3.2.1.58); exo-beta-1,4-glucanase / cellodextrinase (EC 3.2.1.74); glucan endo-1,6-beta-glucosidase (EC 3.2.1.75); mannan endo-beta-1,4-mannosidase (EC 3.2.1.78); cellulose beta-1,4-cellobiosidase (EC 3.2.1.91); steryl beta-glucosidase (EC 3.2.1.104); endoglycoceramidase (EC 3.2.1.123); chitosanase (EC 3.2.1.132); beta-primeverosidase (EC 3.2.1.149); xyloglucan-specific endo-beta-1,4-glucanase (EC 3.2.1.151); endo-beta-1,6-galactanase (EC 3.2.1.164); beta-1,3-mannanase (EC 3.2.1.-); arabinoxylan-specific endo-beta-1,4-xylanase (EC 3.2.1.-); mannan transglycosylase (EC 2.4.1.-); lichenase / endo-beta-1,3-1,4-glucanase (EC 3.2.1.73); beta-glycosidase (EC 3.2.1.-); endo-beta-1,3-glucanase / laminarinase (EC 3.2.1.39); beta-N-acetylhexosaminidase (EC 3.2.1.52); chitosanase (EC 3.2.1.132); beta-D-galactofuranosidase (EC 3.2.1.146); beta-galactosylceramidase (EC 3.2.1.46); ; beta-rutinosidase /alpha-L-rhamnose-(1,6)-beta-D-glucosidase (EC 3.2.1.-); alpha-L-arabinofuranosidase (EC 3.2.1.55) |
| **GH6** | endoglucanase (EC 3.2.1.4); cellobiohydrolase (EC 3.2.1.91); lichenase / endo-beta-1,3-1,4-glucanase (EC 3.2.1.73); lichenase / endo-beta-1,3-1,4-glucanase (EC 3.2.1.73); |
| **GH9** | endoglucanase (EC 3.2.1.4); endo-beta-1,3(4)-glucanase / lichenase-laminarinase (EC 3.2.1.6); beta-glucosidase (EC 3.2.1.21); lichenase / endo-beta-1,3-1,4-glucanase (EC 3.2.1.73); exo-beta-1,4-glucanase / cellodextrinase (EC 3.2.1.74); cellobiohydrolase (EC 3.2.1.91); xyloglucan-specific endo-beta-1,4-glucanase / endo-xyloglucanase (EC 3.2.1.151); exo-beta-glucosaminidase (EC 3.2.1.165); endo-beta-1,4-glucanase (xanthanase) (EC 3.2.1.-) |
| **GH10** | endo-1,4-beta-xylanase (EC 3.2.1.8); endo-1,3-beta-xylanase (EC 3.2.1.32); tomatinase (EC 3.2.1.-); xylan endotransglycosylase (EC 2.4.2.-); endo-beta-1,4-glucanase (EC 3.2.1.4) |
| **GH94** | cellobiose phosphorylase (EC 2.4.1.20); laminaribiose phosphorylase (EC 2.4.1.31); cellodextrin phosphorylase (EC 2.4.1.49); chitobiose phosphorylase (EC 2.4.1.-); cyclic beta-1,2-glucan synthase (EC 2.4.1.-); cellobionic acid phosphorylase (EC 2.4.1.321); beta-1,2-oligoglucan phosphorylase (EC 2.4.1.-) |
| **GH18** | chitinase (EC 3.2.1.14); lysozyme (EC 3.2.1.17); endo-beta-N-acetylglucosaminidase (EC 3.2.1.96); peptidoglycan hydrolase with endo-beta-N-acetylglucosaminidase specificity (EC 3.2.1.-); Nod factor hydrolase (EC 3.2.1.-); xylanase inhibitor; concanavalin B; narbonin |
| **GH20** | beta-hexosaminidase (EC 3.2.1.52); lacto-N-biosidase (EC 3.2.1.140); beta-1,6-N-acetylglucosaminidase (EC 3.2.1.-); beta-6-SO3-N-acetylglucosaminidase (EC 3.2.1.-) |
| **GH23** | lysozyme type G (EC 3.2.1.17); peptidoglycan lyase (EC 4.2.2.n1) also known in the literature as peptidoglycan lytic transglycosylase; chitinase (EC 3.2.1.14) |
| **GH28** | polygalacturonase (EC 3.2.1.15); exo-polygalacturonase (EC 3.2.1.67); exo-polygalacturonosidase (EC 3.2.1.82); rhamnogalacturonase (EC 3.2.1.171); rhamnogalacturonan alpha-1,2-galacturonohydrolase (EC 3.2.1.173); xylogalacturonan hydrolase (EC 3.2.1.-) |
| **GH78** | alpha-L-rhamnosidase (EC 3.2.1.40); rhamnogalacturonan alpha-L-rhamnohydrolase (EC 3.2.1.174); L-Rhap-alpha-1,3-D-Apif -specific alpha-1,3-L-rhamnosidase (EC 3.2.1.-) |
| **GH105** | unsaturated rhamnogalacturonyl hydrolase (EC 3.2.1.172); d-4,5-unsaturated beta-glucuronyl hydrolase (EC 3.2.1.-); d-4,5-unsaturated alpha-galacturonidase (EC 3.2.1.-) |
| **GH26** | beta-mannanase (EC 3.2.1.78); exo-beta-1,4-mannobiohydrolase (EC 3.2.1.100); beta-1,3-xylanase (EC 3.2.1.32); lichenase / endo-beta-1,3-1,4-glucanase (EC 3.2.1.73); mannobiose-producing exo-beta-mannanase (EC 3.2.1.-) |
| **GH30** | endo-beta-1,4-xylanase (EC 3.2.1.8); beta-glucosidase (3.2.1.21); beta-glucuronidase (EC 3.2.1.31); beta-xylosidase (EC 3.2.1.37); beta-fucosidase (EC 3.2.1.38); glucosylceramidase (EC 3.2.1.45); beta-1,6-glucanase (EC 3.2.1.75); glucuronoarabinoxylan endo-beta-1,4-xylanase (EC 3.2.1.136); endo-beta-1,6-galactanase (EC:3.2.1.164); [reducing end] beta-xylosidase (EC 3.2.1.-) |
| **GH39** | alpha-L-iduronidase (EC 3.2.1.76); beta-xylosidase (EC 3.2.1.37); alpha-L-arabinofuranosidase (EC 3.2.1.55); beta-glucosidase (EC 3.2.1.21); beta-galactosidase (EC 3.2.1.23) |
| **GH2** | beta-galactosidase (EC 3.2.1.23) ; beta-mannosidase (EC 3.2.1.25); beta-glucuronidase (EC 3.2.1.31); alpha-L-arabinofuranosidase (EC 3.2.1.55); mannosylglycoprotein endo-beta-mannosidase (EC 3.2.1.152); exo-beta-glucosaminidase (EC 3.2.1.165); alpha-L-arabinopyranosidase (EC 3.2.1.-); beta-galacturonidase (EC 3.2.1.-); beta-xylosidase (EC 3.2.1.37); beta-D-galactofuranosidase (EC 3.2.1.146); |
| **GH31** | alpha-glucosidase (EC 3.2.1.20); alpha-galactosidase (EC 3.2.1.22); alpha-mannosidase (EC 3.2.1.24); alpha-1,3-glucosidase (EC 3.2.1.84); sucrase-isomaltase (EC 3.2.1.48) (EC 3.2.1.10); alpha-xylosidase (EC 3.2.1.177); alpha-glucan lyase (EC 4.2.2.13); isomaltosyltransferase (EC 2.4.1.-); oligosaccharide alpha-1,4-glucosyltransferase (EC 2.4.1.161); sulfoquinovosidase (EC 3.2.1.-); alpha-N-acetylgalactosaminidase (EC 3.2.1.49); ; sulfoquinovosidase (EC 3.2.1.199) |
| **GH43** | beta-xylosidase (EC 3.2.1.37); alpha-L-arabinofuranosidase (EC 3.2.1.55); xylanase (EC 3.2.1.8); alpha-1,2-L-arabinofuranosidase (EC 3.2.1.-); exo-alpha-1,5-L-arabinofuranosidase (EC 3.2.1.-); [inverting] exo-alpha-1,5-L-arabinanase (EC 3.2.1.-); beta-1,3-xylosidase (EC 3.2.1.-); [inverting] exo-alpha-1,5-L-arabinanase (EC 3.2.1.-); [inverting] endo-alpha-1,5-L-arabinanase (EC 3.2.1.99); exo-beta-1,3-galactanase (EC 3.2.1.145); beta-D-galactofuranosidase (EC 3.2.1.146) |
| **GH74** | endoglucanase (EC 3.2.1.4); oligoxyloglucan reducing end-specific cellobiohydrolase (EC 3.2.1.150); xyloglucanase (EC 3.2.1.151) |

(http://www.cazy.org/)

**Appendix 3**


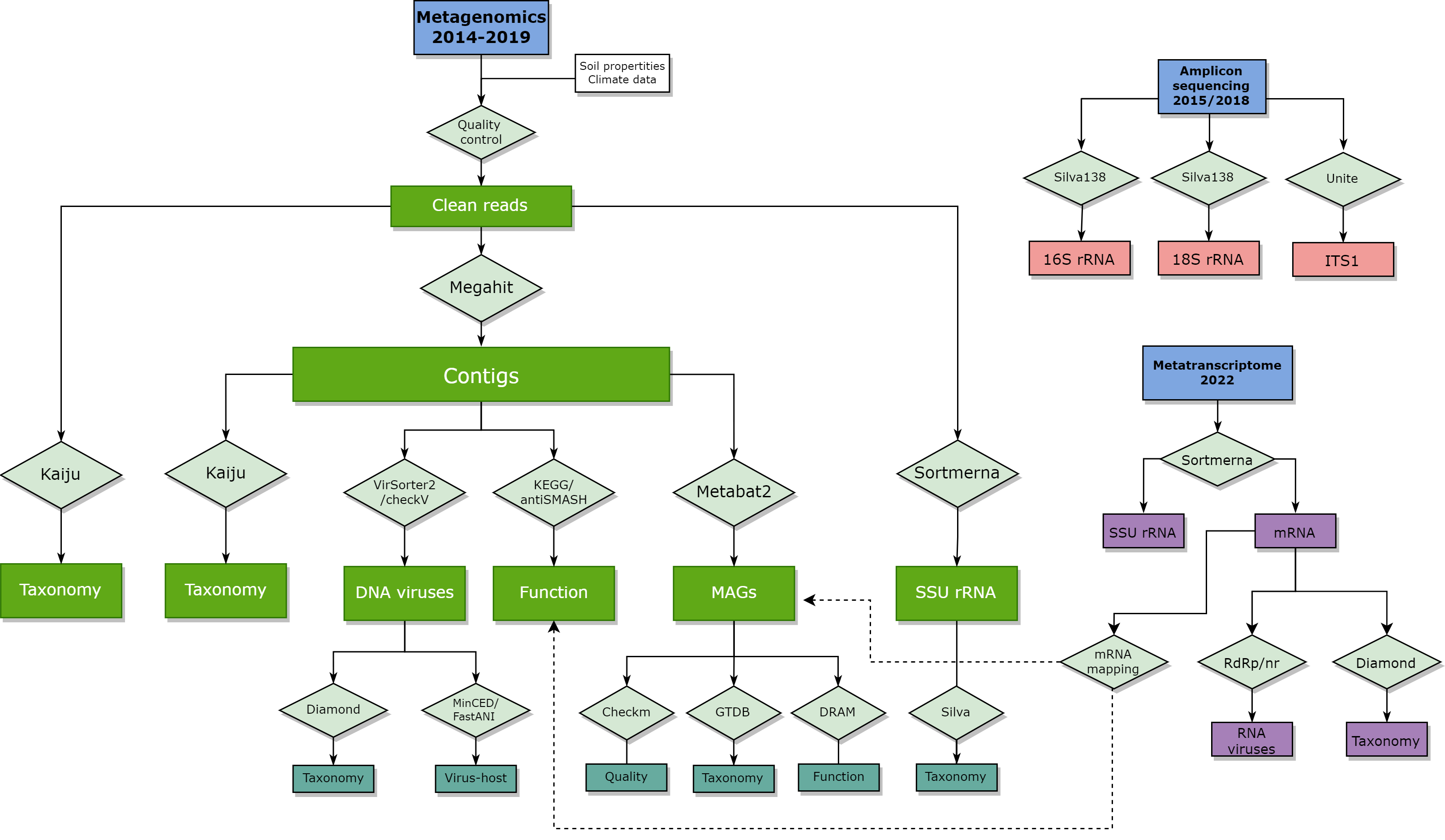

Supplement: Supplementary file 1 — Supplemental_Figures [file 41396_2023_1470_MOESM1_ESM.docx]
